# Chemical Toolkit for PARK7: Potent, Selective, and High-Throughput

**DOI:** 10.1021/acs.jmedchem.2c01113

**Published:** 2022-09-23

**Authors:** Yuqing Jia, Robbert Q. Kim, Raymond Kooij, Huib Ovaa, Aysegul Sapmaz, Paul P. Geurink

**Affiliations:** Oncode Institute & Department of Cell and Chemical Biology, Leiden University Medical Center, Einthovenweg 20, Leiden 2333 ZC, The Netherlands

## Abstract

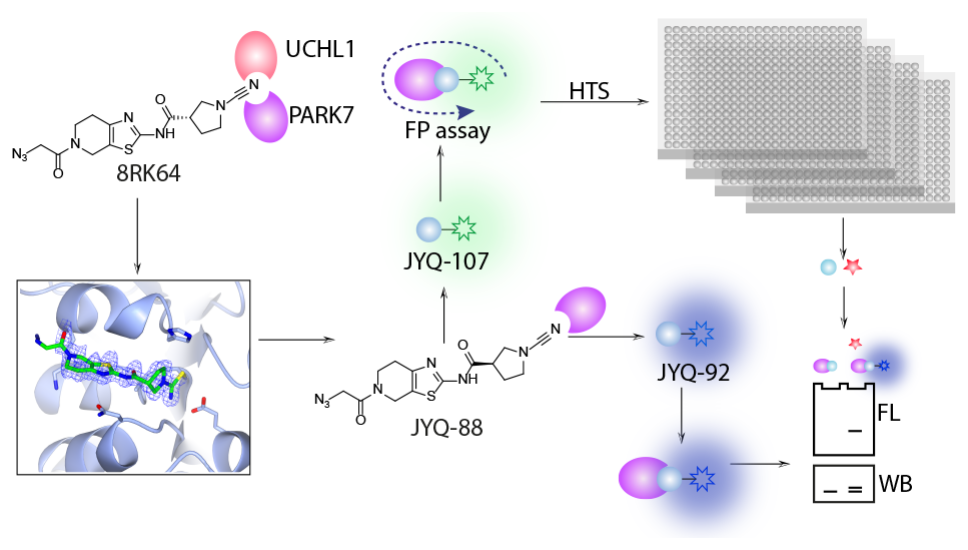

The multifunctional human Parkinson’s disease protein 7 (PARK7/DJ1) is an attractive therapeutic target due to its link with early-onset Parkinson’s disease, upregulation in various cancers, and contribution to chemoresistance. However, only a few compounds have been identified to bind PARK7 due to the lack of a dedicated chemical toolbox. We report the creation of such a toolbox and showcase the application of each of its components. The selective PARK7 submicromolar inhibitor with a cyanimide reactive group covalently modifies the active site Cys106. Installment of different dyes onto the inhibitor delivered two PARK7 probes. The Rhodamine110 probe provides a high-throughput screening compatible FP assay, showcased by screening a compound library (8000 molecules). The SulfoCy5-equipped probe is a valuable tool to assess the effect of PARK7 inhibitors in a cell lysate. Our work creates new possibilities to explore PARK7 function in a physiologically relevant setting and develop new and improved PARK7 inhibitors.

## Introduction

The human Parkinson disease protein 7 (PARK7) was first identified as a mitogen-dependent oncogene (DJ-1) in association with the Ras-related transduction pathway and later discovered to be involved in Parkinson’s disease as a causative autosomal gene.^1−3^ It is a small multifunctional protein (20 kDa) containing 189 amino acids, which participates in transcriptional regulation^4^ and mitochondrial regulation,^5^ and acts as a molecular chaperone,^6^ oxidative stress sensor,^7^ and glyoxalase.^8^ It has also been described as a protein and nucleotide deglycase;^9^ however, it was recently reported that this function could be attributed to its glyoxalase activity.^10,11^ The deletion and missense mutations in the *PARK7* gene, affecting its stability and function, are associated with Parkinson’s disease.^12−15^ The upregulation of PARK7 is involved with various types of cancer, and the high serum level of PARK7 is positively correlated with tumorigenesis, metastasis, and prognosis.^16^ Moreover, the protective function of PARK7 against oxidative stress-induced apoptosis has been related to chemoresistance of cancer cells, and a recent study has suggested that PARK7 possesses antiferroptosis function,^17−21^ suggesting that impeding PARK7 function in these pathways could be a new strategy to reduce chemoresistance by combination drug therapy. Altogether, PARK7 could be the candidate drug target to improve therapies toward cancer and neurodegenerative diseases and serve as a potential diagnostic and prognostic biomarker.

**Figure 1:**
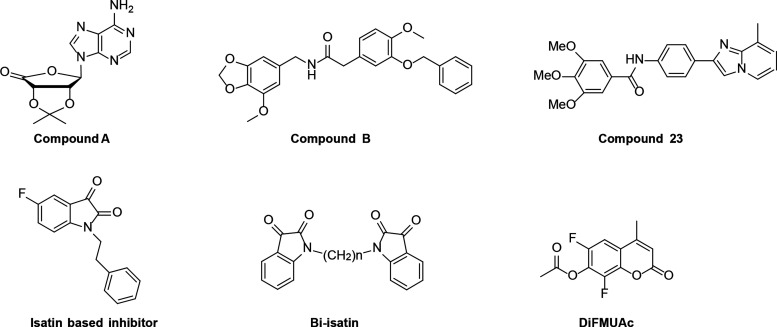
Representative PARK7 inhibitors and the DiFMUAc assay reagent.

Although PARK7 has been widely studied for more than two decades because of its multifunctional roles and its link to both cancer and neurodegenerative diseases, potent and selective PARK7 inhibitors are remarkably scarce.^22−25^ Recently, the level of PARK7 has been shown to be downregulated by several anticancer drug candidates.^26−28^ However, it is still obscure whether the effect of PARK7 downregulation by these drugs is due to the scaffolding function of the full-length protein or its catalytic activity regulated by the highly conserved cysteine residue Cys106. Therefore, finding compounds specifically binding to active site Cys106 that can serve as tools to differentiate scaffolding and enzymatic function of PARK7 contributing to disease pathogenesis or as therapeutic drugs is currently emerging. The first compounds identified to bind with PARK7 using virtual screening were compound A (UCP0045037) and compound B (UCP0054278), followed by compound 23, which was more potent in preventing oxidative stress-induced neuronal cell death (Figure 1).^23,24^ One decade later, a family of PARK7 inhibitors based on isatin, an endogenous metabolite, was identified in an SPR screen on a 100 compound fragment library.^22^ A series of bis-isatin compounds were further developed to target homodimer DJ-1.^25^ Recently, the development of a PARK7 activity assay that relies on the deacetylation of the fluorogenic substrate 6,8-difluoro-4-methylumbelliferyl (DiFMUAc) was reported.^29^ The fluorescence read-out at ex 358/em 455 nm allows for continuous monitoring of PARK7 activity, thereby providing a substantial improvement compared to conventional methods that rely on indirect colorimetric read-out.^30^ This assay was used to optimize earlier reported isatin-based PARK7 inhibitors, which resulted in, among other covalent inhibitors, compound 26.

Despite these studies, the attempts to establish PARK7 inhibitors do not meet the demand for them to understand PARK7 biology and to further use as therapeutic agents for diseases yet, implying a significant challenge in the field.

The key challenge to identify PARK7 inhibitors is the lack of appropriate chemical tools to study PARK7, such as reagents for a high-throughput-compatible biochemical assay or chemical probes to assess PARK7 activity in vitro or in a cellular context. Therefore, we reasoned that developing a chemical probe that potently and selectively binds to PARK7 would serve a dual purpose: (1) as a high-throughput assay reagent to screen for inhibitors; (2) as a chemical tool to visualize PARK7 in cellular context. We here report the development of a selective small-molecule PARK7 inhibitor (**JYQ-88**) and show how it covalently modifies the active site cysteine residue as evidenced from biochemical experiments as well as X-ray crystallography. Equipping the inhibitor with different fluorescent groups resulted in two chemical probes: The Rhodamine probe **JYQ-107** was used in a fluorescence polarization-based assay (FP assay) for fast generating PARK7 inhibitors, which we showcase by the high-throughput screening of a covalent fragment library containing ∼8000 molecules. The SulfoCy5 probe **JYQ-92** was used to label and visualize PARK7 in an HEK293T cell lysate, showing its potential use as an orthogonal assay to validate the hit compounds in a cell lysate.

## Results and Discussion

### Development and Characterization of PARK7 Inhibitors

**Figure 2:**
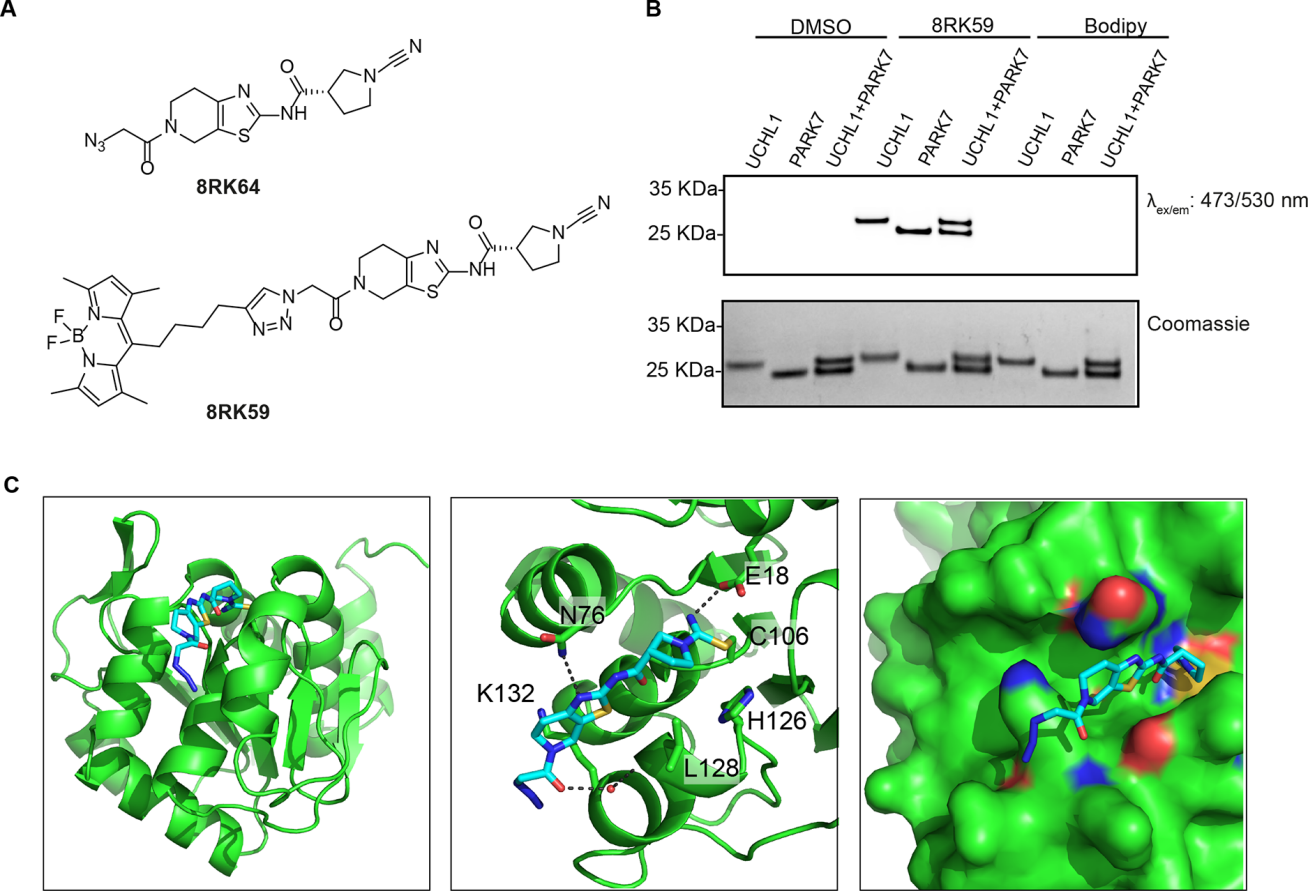
Selectivity analysis of **8RK59** toward UCHL1 and PARK7 and structural characterization of **8RK64** binding to PARK7. (A) Structures of **8RK64** and **8RK59**. (B) Labeling of PARK7 and UCHL1 with **8RK59**. UCHL1 and PARK7 were incubated with **8RK59**, resolved by SDS-PAGE, and analyzed by fluorescence scanning (top) and Coomassie staining (bottom). DMSO and BodipyFL were used as negative controls. (C) Co-crystal structure of the **8RK64**-PARK7 complex (PDB: 7PA2). PARK7 in green and **8RK64** in cyan.

We recently reported a small-molecule cyanimide inhibitor (**8RK64**) and activity-based probe (**8RK59**) for the deubiquitinase (DUB) UCHL1 (Figure 2A) and showed PARK7 to be the major off-target.^31^ To determine the labeling efficiency of **8RK59** for both UCHL1 and PARK7, a mixture of equal amounts of purified recombinant UCHL1 and PARK7 (1 μM each) is labeled with **8RK59** (2 μM). Sodium dodecyl sulfate–polyacrylamide gel electrophoresis (SDS-PAGE) followed by fluorescence scanning revealed that PARK7 and UCHL1 were labeled to a nearly equal extent (Figure 2B). We reasoned that the structure of **8RK64** could serve as a starting point for developing more selective inhibitors for PARK7. To gain more insight into the inhibitor binding mode and to guide the design of novel PARK7 inhibitors, the crystal structure of the PARK7–**8RK64** complex was solved with 1.21 Å resolution (Figure 2C, Table S1, PDB 7PA2). The structure is obtained as a monomer and compared to its apo structure (PDB 1J42). The overall PARK7 structure has not changed,^32^ indicating that **8RK64** binding does not affect PARK7 folding. Globally, the inhibitor fits into a groove between two α-helices formed by residues 76–84 and 126–134 (Figure 2C, left panel). The covalent bond between **8RK64** and PARK7 is clearly visible (Figure 2C, middle panel). The cyanimide moiety of the inhibitor reacted to active site Cys106, thereby forming an isothiourea linkage, which is similar to what has been observed for the reaction of cyanamide-based and azanitrile inhibitors with cathepsins.^33,34^ The isothiourea moiety is stabilized by an H-bond between the NH and the carboxylate side chain of the nearby Glu18. Another interaction is found between the thiazole ring amine in the inhibitor and the amide NH_2_ of the Asn76 side chain. Hydrogen bonding between the carbonyl of the azidoacetyl moiety and the Leu128 backbone carbonyl via a water molecule is also observed.

**Scheme 1:**
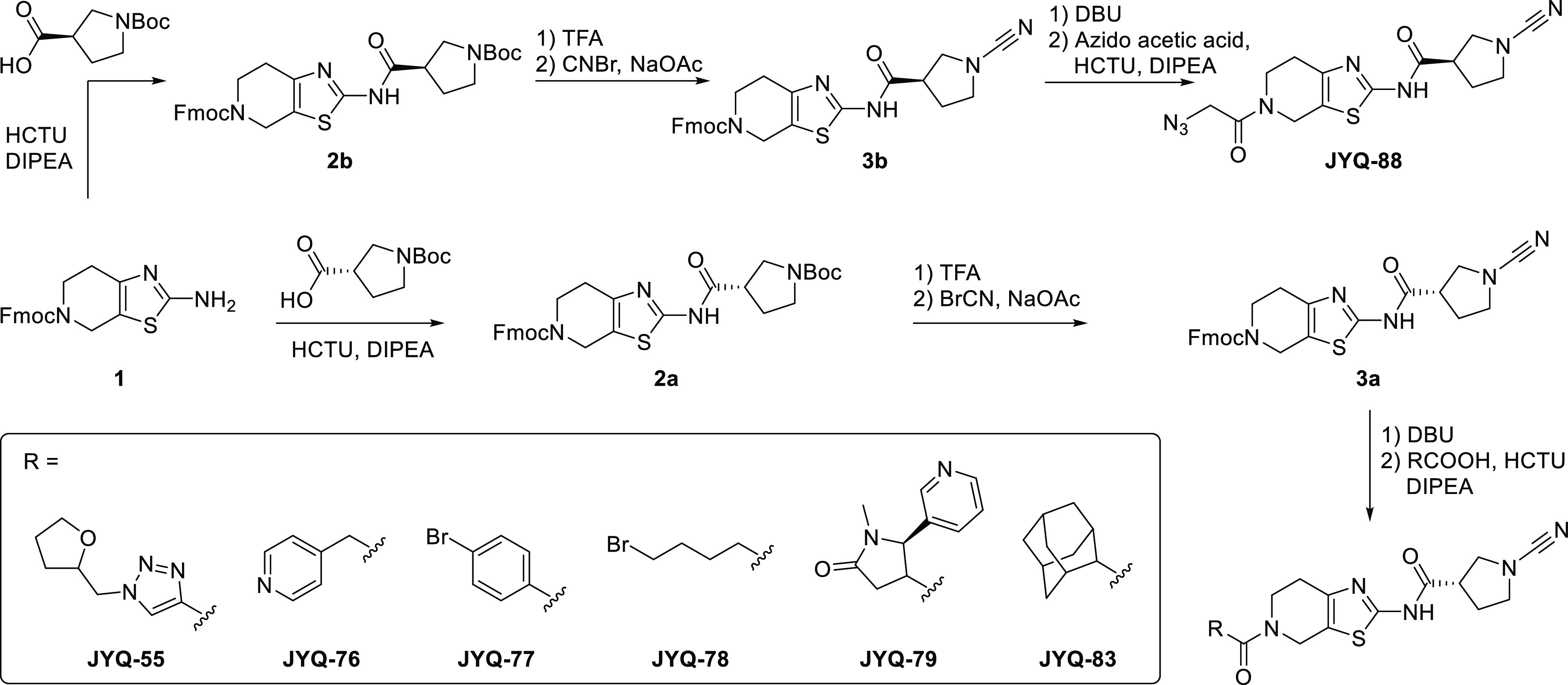
Synthetic Route toward PARK7 Inhibitors

To improve the inhibitor, we first opted to keep the above-mentioned interactions intact as much as possible. From the structure, we observed an unoccupied space in the area surrounding the azide moiety of **8RK64** (Figure 2C, right panel). We decided to investigate whether introducing a more bulky group at this site could enhance the binding between PARK7 and **8RK64** and**/**or lead to a better selectivity for PARK7 over UCHL1. A small panel of variations was made by replacing the azide moiety for larger groups using an altered synthetic procedure to synthesize **8RK64** (Scheme 1).^35^ Compound **2a** was formed in an amide coupling between compound **1** and (*S*)-1-(*tert*-butoxycarbonyl)pyrrolidine-3-carboxylic acid. The cyanimide moiety was introduced by first removing the Boc protecting group, followed by a reaction with cyanogen bromide to form **3a**. After Fmoc deprotection, different carboxylic acids were coupled, which yielded compounds **JYQ-55**, **JYQ-76**, **JYQ-77**, **JYQ-78**, **JYQ-79**, and **JYQ-83** (Scheme 1). From the crystal structure, it was unclear whether the stereochemistry of the inhibitor substantially contributed to the interaction with PARK7, so we also opted to investigate the role of the chiral center in **8RK64**. Its enantiomer was synthesized following the same synthesis route but instead coupling (*R*)-1-(*tert*-butoxycarbonyl)pyrrolidine-3-carboxylic acid in the first step, leading to compound **2b**, then **3b**, and finally **JYQ-88** after coupling azidoacetic acid in the final step.

**Figure 3:**
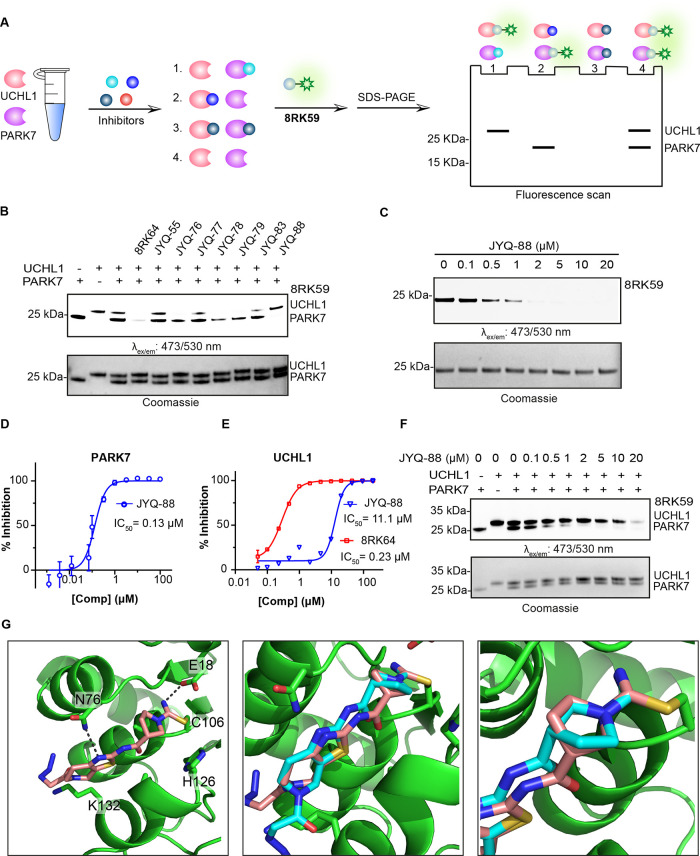
Selectivity analysis of the inhibitors and structural characterization of **JYQ-88** binding to PARK7. (A) Schematic representation of the gel-based competition assay to identify selective PARK7 inhibitors. (B) Activity and selectivity evaluation of PARK7 inhibitors. PARK7 and UCHL1 (1 μM) were incubated with indicated compounds (2 μM), followed by incubation with **8RK59**. (C) Activity evaluation of **JYQ-88** for PARK7. PARK7 (1 μM) was incubated with increasing concentrations of **JYQ-88**, followed by incubation with **8RK59**. (D) IC_50_ determination of **JYQ-88** for PARK7 by the DiFMUAc assay. (E) IC_50_ determination of **JYQ-88** and **8RK64** for UCHL1. (F) Selectivity determination of inhibitor **JYQ-88** between UCHL1 and PARK7. PARK7 and UCHL1 were incubated with a serial dilution of **JYQ-88**, followed by incubation with **8RK59**. (G) Co-crystal structure of the PARK7–**JYQ-88** complex (PDB:7PA3). PARK7 in green, **JYQ-88** in pink, and **8RK64** in cyan. Overlay between **8RK64** and **JYQ-88** is shown in the middle panel with a zoom-in shown on the right. The cyanopyrrolidine moiety has rotated 180°.

To assess the inhibitory potency and selectivity of these compounds toward PARK7 and UCHL1, we performed a gel-based competition assay (Figure 3A).^36^ A mixture of equal amounts of PARK7 and UCHL1 (1 μM) was treated with each of the synthesized compounds (2 μM) for 1 h, followed by incubation with fluorescent probe **8RK59** (2 μM) for 30 min. Samples were resolved by SDS-PAGE, and the gel was scanned for fluorescence. Inhibition of PARK7 or UCHL1 is reflected by the disappearance of their corresponding bands (Figure 3B). This revealed that compounds **JYQ-76**, **JYQ-78**, and **JYQ-79** fully inhibit UCHL1 similar to **8RK64** while affecting PARK7 activity less efficiently. On the other hand, **JYQ-55**, **JYQ-77**, and **JYQ-83** hardly show any inhibition. Interestingly, **JYQ-88** is the only compound that shows almost complete inhibition of PARK7 activity without inhibiting UCHL1. The inhibition potential of **JYQ-88** against PARK7 was assessed in a similar assay where PARK7 was incubated with a serial dilution of **JYQ-88** before treatment with probe **8RK59** (Figure 3C). A clear inhibition is visible at 0.5 μM, and full inhibition is achieved between 1 and 2 μM. The IC_50_ value was determined to be 0.13 μM using the reported DiFMUAc PARK7 activity assay (Figure 3D).^29^ In contrast, **JYQ-88** inhibits UCHL1 with an IC_50_ of 11.1 μM, as determined in a UCHL1 activity assay (Figure 3E),^31^ thus showing a 85-fold higher potency toward PARK7. Compared to its enantiomer (**8RK64**), **JYQ-88** shows a >50-fold potency drop toward UCHL1. The selectivity of **JYQ-88** between UCHL1 and PARK7 was further confirmed in a direct competition assay between equal amounts of PARK7 and UCHL1 (Figure 3F). PARK7 inhibition was observed starting from 0.5 μM **JYQ-88**, whereas inhibition of UCHL1 was only detected at 20 μM. Since **JYQ-88** and **8RK64** are enantiomers, we assumed that the interactions with PARK7 should be similar. To investigate how the chiral center influences the selectivity, we solved the crystal structure of the PARK7−**JYQ-88** complex with 1.42 Å resolution (Figure 3G, Table S1, PDB 7PA3). The global positioning of the inhibitor in between the two α-helices was similar to that for **8RK64**. Interestingly, the cyanopyrrolidine moiety has rotated 180° around the single bond that connects the carbonyl and pyrrolidine ring to accommodate for the inversion of the stereocenter. This way, the thiazolo-pyridine part of **JYQ-88** is bound in a similar manner as **8RK64** and the earlier observed interactions are largely the same. The formation of the isothiourea moiety, stabilized by the interaction with Glu18, and the interaction between the thiazole amine and the amide NH_2_ of the Asn76 side chain are still present. However, the interaction between the azidoacetyl carbonyl with the Leu128 backbone carbonyl can no longer be observed due to the rotation of the azidoacetyl moiety. The Lys132 residue also seems to have rotated (compared to Figure 2C).

The interactions between PARK7 and both compounds, deduced from the X-ray structures, do not directly indicate which of the two compounds would possess a better binding capacity. Nevertheless, the PARK7 inhibition assays revealed that the inhibitory potency of both compounds toward PARK7 is similar, with **JYQ-88** being slightly more potent (Figures 3C and S2). The superiority of **JYQ-88** over **8RK64** thus originates from the large gain in specificity toward PARK7 because of the loss of UCHL1 inhibition (Figure 3E). Due to the above described 180° rotation of the cyanopyrrolidine moiety, the inversion of the stereocenter is not detrimental to PARK7 binding, and it is possible that this cannot occur for UCHL1. Unfortunately, despite several attempts, we were unable to obtain structural information of the compound binding to UCHL1 to validate our hypothesis.

### From Inhibitor to Activity-Based Probes

**Figure 4:**
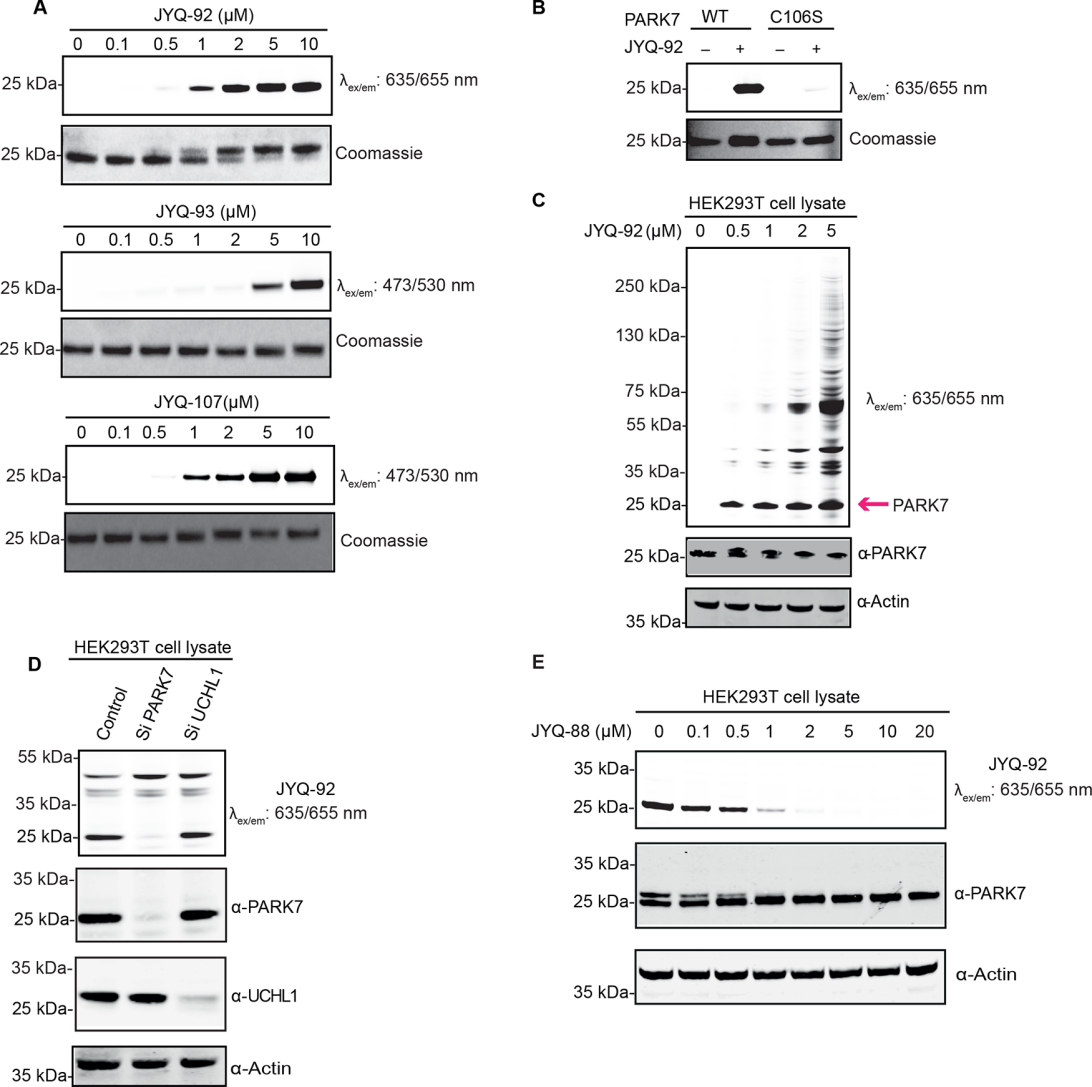
Biochemical characterization of the fluorescent PARK7 probes. (A) Labeling efficiency of **JYQ-92**, **JYQ-93**, and **JYQ-107** with purified PARK7. PARK7 was incubated with increasing concentrations of the probes. (B) **JYQ-92** labels purified recombinant PARK7 WT but not Cys106Ser mutant. (C) Fluorescence labeling of PARK7 activity in the HEK293T cell lysate. The pink arrow indicates the band for PARK7. (D) Fluorescence labeling by **JYQ-92** in the HEK293T cell lysate with/without depletion of PARK7 or UCHL1.The prepared cell lysate was incubated with **JYQ-92** (1 μM final concentration) for 1 h. (E) Fluorescence labeling of PARK7 remaining activity by **JYQ-92** after treatment with inhibitor **JYQ-88**. The prepared cell lysate was incubated with a dilution series of **JYQ-88**, followed by incubation with **JYQ-92** (1 μM). HEK293T cell lysates treated as indicated above were analyzed by immunoblotting against total UCHL1 (rabbit anti-UCHL1 antibody, 1:1000) and PARK7 (rabbit anti-PARK7 antibody, 1:1000), with actin (mouse anti-actin antibody, 1:10,000) as the loading control. Relevant antibodies used in each gel are indicated using the “α” symbol in front of the protein name.

**Scheme 2:**
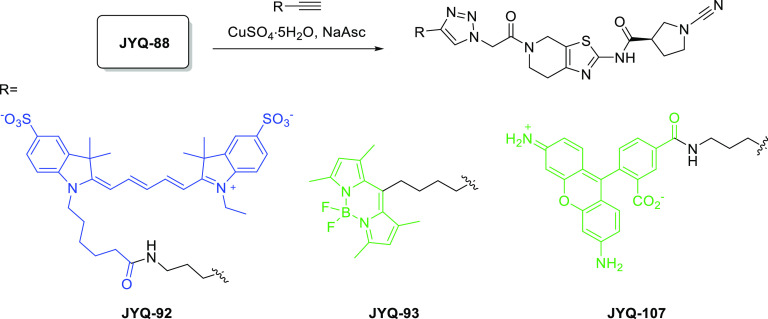
Construction of Fluorescent PARK7 Probes

PARK7 inhibitor **JYQ-88** was further converted into activity-based probes by installing SulfoCy5, Rhodamine110, or BodipyFL. We took advantage of the azide moiety in **JYQ-88** and conjugated alkyne versions of these dyes using the copper(I)-catalyzed azide alkyne cycloaddition (CuAAC) to obtain compounds **JYQ-92**, **JYQ-93**, and **JYQ-107** (Scheme 2). We next investigated the ability of the probes to label and visualize PARK7. Purified PARK7 (1 μM) was treated with a concentration series of each probe and incubated for 1 h at 37 °C, followed by SDS-PAGE analysis. Fluorescence scanning and Coomassie staining of the gels revealed that all three probes could label PARK7 (Figure 4A). The SulfoCy5 probe **JYQ-92** and Rhodamine110 probe **JYQ-107** labeled PARK7 already at 1 μM, while the BodipyFL probe **JYQ-93** was less efficient than the other probes as PARK7 labeling was only observed from 5 μM. Even a clear band shift was observed with probe **JYQ-92** (1080 Da) but not for **JYQ-107** and **JYQ-93**, which can likely be attributed to the difference in the molecular weight of the probes (Figure 4A). Determination of the IC_50_ values toward UCHL1 (∼20 μM for all probes) revealed that the selectivity for PARK7 is retained (Figure S4).

### **JYQ-92** Selectively Binds PARK7 in a Cell Lysate

Based on the potency and convenience of visualizing PARK7, we chose to continue with SulfoCy5 probe **JYQ-92**. To confirm that **JYQ-92** binds only to active PARK7, wild-type PARK7 and its catalytic inactive C106S mutant were incubated with 2 μM **JYQ-92** for 1 h. **JYQ-92** only binds to the active wild-type PARK7 but not to the inactive C106S mutant, confirming that the probe binds to the active site cysteine (Figure 4B). We next investigated the ability of **JYQ-92** to label PARK7 activity in HEK293T cell lysates by treating with the increasing concentration of **JYQ-92** (0–5 μM) for 1 h. A clear band was observed at 20 kDa already at 0.5 μM after fluorescence scanning, which corresponds to the molecular weight of PARK7, and labeling of PARK7 was further confirmed by western blotting as evidenced from the appearance of a slightly higher running band (Figure 4C). In addition to this band, other bands (higher *M*_w_) gradually increased as the concentration of **JYQ-92** increased above 1 μM, likely due to the aspecific labeling of other proteins. This provides a concentration window between 0.5 and 1 μM in which only PARK7 is labeled in the presence of other proteins. To study its specificity toward PARK7 compared to UCHL1 in more detail, HEK293T cell lysates where we knockdown PARK7 or UCHL1 were treated with 1 μM final concentration of **JYQ-92**. A clear PARK7 labeling band appeared at the expected height in the control sample, while this band is virtually absent in the PARK7 knockdown sample (Figure 4D). Moreover, we did not observe the disappearance of any band in the fluorescence labeling of the UCHL1 knockdown cell lysate. These data show that **JYQ-92** binds to PARK7 but not to UCHL1 in the cell lysate.

We next assessed whether we could measure the inhibitory activity of an inhibitor against PARK7 in a cell lysate using **JYQ-92** to label the remaining activity of PARK7. The HEK293T cell lysate was incubated with a serial dilution of inhibitor **JYQ-88** for 1 h at 37 °C, followed by incubation with **JYQ-92** for 30 min at 37 °C and analysis by fluorescence scanning after SDS-PAGE (Figure 4E). The disappearance of the labeling band is a measure for inhibition. These results clearly show a dose-dependent inhibition of PARK7 activity by **JYQ-88**. The band intensity is already decreased at 0.1 and 0.5 μM and has nearly disappeared from 1 μM, proving that **JYQ-88** potently inhibits PARK7 in a cell lysate.

Due to the structural similarity of PARK7 inhibitor **JYQ-88** and UCHL1 inhibitor **8RK64** and the fact that many cyanimide-containing compounds have been identified as DUB inhibitors,^35,37^ we investigated the inhibitory potential of **JYQ-88** and **JYQ-92** toward DUBs. After treating the HEK293T cell lysate with **JYQ-88** and **JYQ-92**, along with UCHL1 inhibitors, **6RK73** and **8RK64** as controls,^38^ the remaining activity of DUBs was determined using the general DUB probe Rho-Ub-PA (Figure S5).^39^ As expected, we only observed disappearance of the UCHL1 band with UCHL1 inhibitors **6RK73** and **8RK64**. In contrast, no band disappeared after treatment with **JYQ-88** and **JYQ-92**, indicating no influence on DUB activity in the HEK293T cell lysate.

Altogether, SulfoCy5 probe **JYQ-92** labels PARK7 selectively at concentrations below 1 μM and can be used to monitor PARK7 inhibition in a cell lysate. Our results showed that **JYQ-88** can inhibit PARK7 in a cell lysate without showing any effect on the activity of cysteine DUBs that are visualized by activity-based profiling.

### **JYQ-107** Can Be Applied in the High-Throughput Fluorescence Polarization Assay

One of the main challenges for PARK7 inhibitor identification is the development of an appropriate biochemical assay that is suited for the high-throughput screening (HTS) of thousands of molecules. Ideally, such an assay has simple, automatable handlings, requires small volumes, can be performed in 384 or 1536 well plates, and has a fast, direct, and easy read-out with little interference from the screening compounds.^40^ As our probes potently and selectively bind active PARK7, we reasoned that these tools could be used to set up a FP assay based on fluorescent probe binding. This assay principle is also known as Fluopol-ABPP and has successfully been applied in multiple HTS campaigns on different targets.^38,41−43^

**Figure 5:**
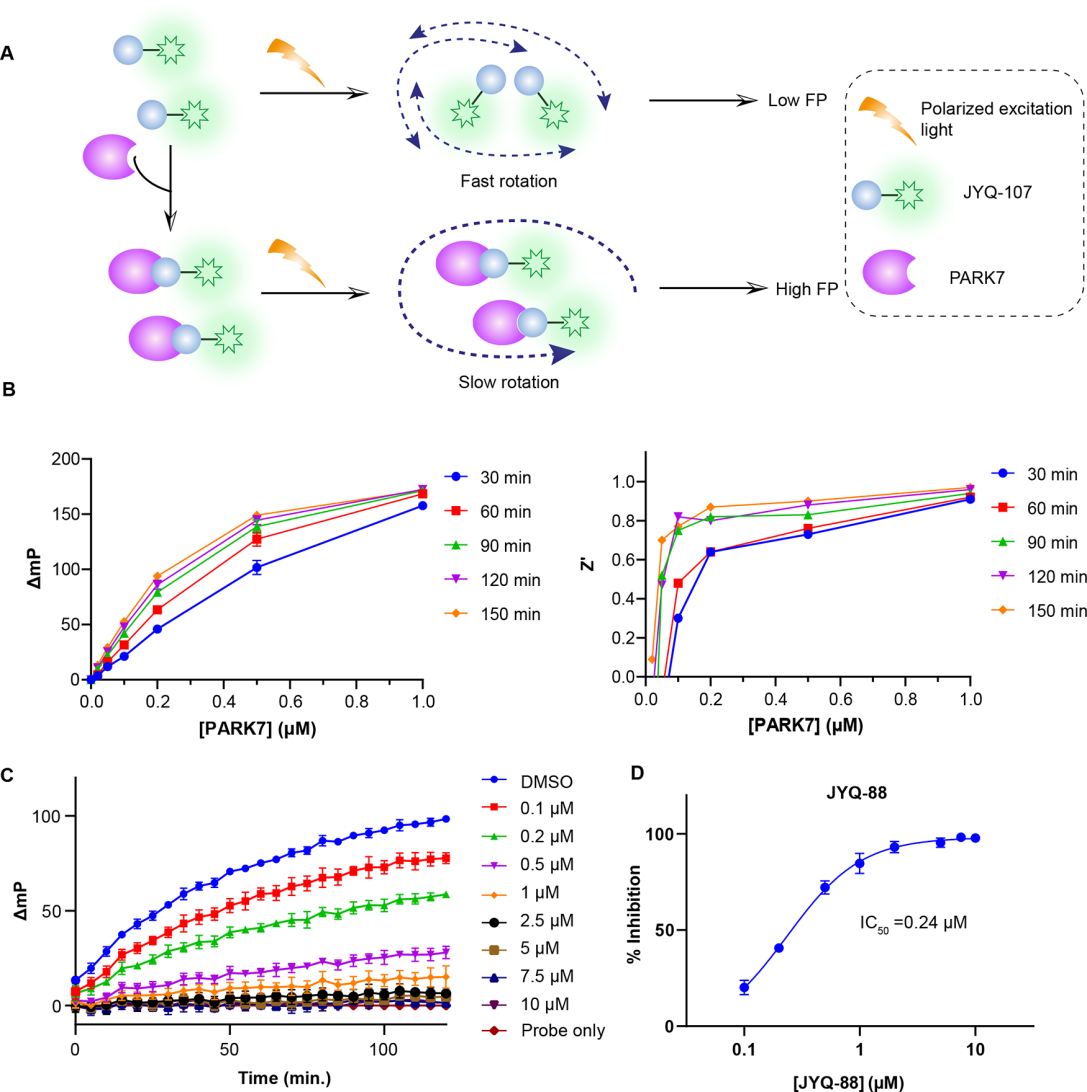
FP assay to identify PARK7 inhibitors. (A) Schematic illustration of the FP assay. (B) Relation between the FP value of **JYQ-107** and PARK7 concentration at different incubation times (left) and corresponding *Z*’ values (right). The full data set up to 5 μM PARK7 is shown in the Supporting Information (see Figure S6). (C) Change in the FP value over time at different concentrations of inhibitor **JYQ-88**. (D) Dose-dependent inhibition of PARK7 by **JYQ-88** determined from the FP data in panel C.

When the fluorescent probe is excited by polarized light, it will emit largely depolarized light if it is in a free, unbound state. When bound to a high molecular weight molecule, such as a protein, the emitted light remains polarized. The change in polarization is a quantitative measure of the probe binding to PARK7, and this can be disrupted by inhibitor binding (Figure 5A).^44^ We chose to use Rhodamine110 probe **JYQ-107** for the FP assay since it shows equal labeling efficiency with **JYQ-92**, and its excitation and emission wavelengths are compatible with the FP filter set in our plate reader.

First, the relation between the change in FP values for **JYQ-107** binding to PARK7 was determined. We used a fixed concentration (20 nM) of **JYQ-107**, incubated it with different amounts of PARK7 (0 to 5 μM), and monitored the FP signal (λ_ex/em_ 485/520 nm) over time (Supporting Information Figure S6A). The change in FP (Δ*mP*) was plotted against PARK7 concentration after different incubation times (Figure 5B, left, Supporting Information Figure S6B). This revealed a positive correlation between the change in FP values and both PARK7 concentration and incubation time. The latter is expected, as covalent complex formation has a time-dependency, and thus, equilibrium binding values could not be determined for the FP reagent.^45^ Instead we calculated *k*_inact_/*K*_I_ for **JYQ-107** to be 1175 M^–1^ s^–1^ (Supporting Information Figure S6D) and we set out to determine optimal assay conditions for HTS. To this end, we calculated the *Z*’ value, a critical parameter used to assess the quality and HTS suitability of a given assay, for the used PARK7 concentrations and incubation times (Figure 5B, right, Supporting Information Figure S6C).^46^ Based on these findings, we decided that a concentration of 0.2 μM PARK7 in combination with 120 min incubation time would be the best choice since it corresponds to a good trade-off between a low amount of required enzyme versus a proper assay window (Δ*mP* ∼ 80) with an excellent *Z*’ value (0.8). To prove that the assay can be used to screen for inhibitors, we applied it to determine the PARK7 inhibition efficiency of **JYQ-88**. A concentration series of **JYQ-88** was incubated with 0.2 μM PARK7 for 1 h, followed by the addition of **JYQ-107** and monitoring the FP signal over time. The percentage inhibition of PARK7 was calculated from the FP values, normalized to probe-only (positive control, 100% inhibition) and dimethyl sulfoxide (DMSO)-treated (negative control, 0% inhibition) samples. These values were plotted against **JYQ-88** concentration to obtain an IC_50_ value of 0.24 μM (Figure 5C,D). As a negative control, inactive compound, **JYQ-55** was taken along, and as expected, no inhibition of PARK7 was observed (Supporting Information Figure S7). This is well in line with the results from the gel-based assays (Figure 3C,F) and DiFMUAc activity assay (Figure 3D) and demonstrates the suitability of our FP assay to screen for inhibitors.

**Figure 6:**
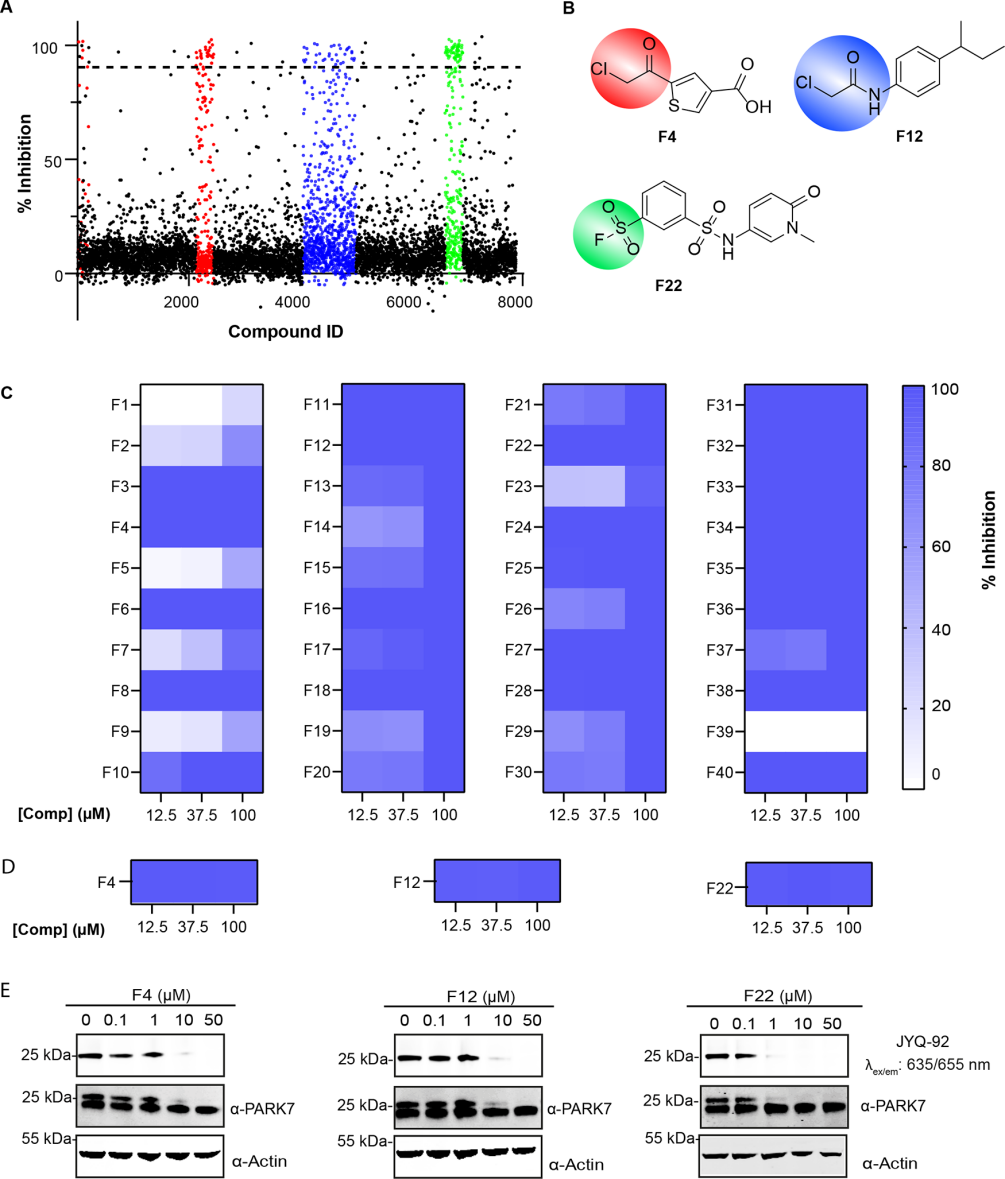
High-throughput PARK7 inhibitor screen on a covalent fragment library using the FP assay. (A) Screening results at 100 μM compound concentration. Colors represent three different compound groups with the highest hit rate. (B) Structures of representative hits. Colors correspond to those in Figure A. (C) Heatmap displaying the validation of the screening hits at 12.5, 37.5, and 100 μM using the FP assay. (D) Heatmap displaying the validation of the representative hits at 12.5, 37.5, and 100 μM using the DiFMUAc assay. (E) Validation of representative hits in a cell lysate. The prepared cell lysate was incubated with a dilution series of each compound, followed by incubation with **JYQ-92** (1 μM).

To showcase the usefulness of our FP assay in a high-throughput screen, we opted to screen a commercially available covalent fragment library containing 7887 small molecules. The screen was performed in a 1536 well plate format with a total volume of 8 μL per well at 100 μM final compound concentration. A total of 150 compounds comprising various types of reactive groups showed over 90% inhibition (Figure 6A, see the Supporting Information for full screening data). Since the majority of these hits contain a sulfonyl fluoride, chloromethylketone, or chloroacetamide warhead (see colored clusters in Figure 6A,B), we selected 40 compounds from these categories for hit validation. Exemplary structures representative for the most potent screening hits are shown in Figure 6B. The compounds were cherry-picked and tested at three different concentrations, 100, 37.5, and 12.5 μM in triplicate (Figure 6C, compound structures are shown in Table S3). Five compounds could not be validated. From the remaining compounds, 22 showed complete inhibition already at 12.5 μM. The hits were also successfully validated at the same concentrations using the DiFMUAc assay as an orthogonal read-out (Figure 6D and Supporting Information Figure S8). Finally, three of the best hits were selected (F4, F12, and F22) and their ability to inhibit PARK7 in a cell lysate was confirmed using the above described (see Figure 4E) SulfoCy5 probe **JYQ-92**-based assay. All three compounds proved to be also inhibiting PARK7 in a cell lysate with compound F22 already showing (almost) full inhibition at 1 μM (Figure 6E). These findings clearly demonstrate the applicability of our probes in HTS of thousands of molecules and validation of screening hits on isolated PARK7 and in the cell lysate. The validated hits can provide new starting points for future inhibitor development.

## Conclusions

PARK7 is a potential therapeutic target due to its involvement in various diseases. Yet, compared to many other therapeutically interesting enzymes such as kinases, DUBs, and cathepsins, the chemical toolbox required to study PARK7 is nearly empty.^47−49^ Potent inhibitors, assay reagents, and activity-based probes, which are considered indispensable tools to understand the functions of proteins, have largely been lacking for PARK7 so far. Here, we report the development of a selective small-molecule PARK7 inhibitor equipped with a cyanimide reactive group that targets the active site Cys106. The compound was based on our recently reported UCHL1 inhibitor that has PARK7 as an off-target.^31^ We attempted to optimize the potency and selectivity for PARK7 over UCHL1 by designing new analogues based on X-ray crystallography and surprisingly found our best compound, **JYQ-88**, to be the enantiomer of the original compound. The co-crystal structure of **JYQ-88** in complex with PARK7 revealed that a 180° rotation of the cyanopyrroldine moiety likely compensates for the inverted strereocenter, and we hypothesize that this cannot occur for UCHL1, which could be the reason behind the selectivity of **JYQ-88** for PARK7 over UCHL1. Nearly all of the published PARK7 inhibitors are based on isatin or an indole structural motif.^22,25,29^ With an IC_50_ of 0.13 μM, **JYQ-88** is of similar potency compared to the most potent reported PARK7 inhibitors (Figure S3), yet it represents a novel class of PARK7 inhibitors with distinct structural features and irreversible mode of action. Notably, it is the first PARK7 inhibitor that shows target engagement in a cell lysate. So far, we have not been able to show this effect in cells, which leaves room for further improvement. Fortunately, the development of the here described PARK7 reagents will enable the identification of future PARK7 inhibitors with improved features.

Often, the identification of new inhibitors starts with the HTS of several thousands of molecules. The existence of a suitable assay is a key requirement for this. Such an assay needs to fulfill certain criteria: (1) it should have an easy read-out, (2) requires low concentrations of protein and substrate, and (3) should be automatable and possible in a multiwell plate. Commonly used activity assays for PARK7 rely on its glyoxalase activity and require methylglyoxal (MGO) or phenylglyoxal as the substrate.^11,22^ The consumption of MGO (and hence PARK7 activity) is indirectly quantified after treatment with 2,4-dinitrophenylhydrazine (DNPH) by monitoring the DNPH absorbance at 550 nm. As MGO is converted to l-lactate by PARK7, another option to indirectly monitor its activity is by determining l-lactate levels.^50^ As these methods rely on indirect, multistep read-outs and require high concentrations of all components and large volumes, their application in HTS campaigns is not so straightforward. The consumption of phenylglyoxal can directly be measured via its absorbance at 250 nm, but this will in many cases result in interference of UV-active small molecules and also requires large volumes and high enzyme and substrate concentrations for a proper read-out.

To address these shortcomings, we equipped inhibitor **JYQ-88** with a Rhodamine110 dye, resulting in PARK7 probe **JYQ-107**, and used this to set up an FP assay for rapidly identifying PARK7 inhibitors. This assay has multiple advantages compared to reported PARK7 (glyoxalase activity) assays, such as easy detection, low protein and substrate concentrations, and practical application in HTS, as we showcased by the successful screening of a 7887 member compound library. We identified several competitive fragment hits with various warheads which by themselves could serve as starting points for inhibitor development.

In our study, we also made use of the recently reported DiFMUAc fluorescence assay,^29^ which relies on the deacetylation of the fluorogenic substrate 6,8-difluoro-4-methylumbelliferyl, as an orthogonal assay to successfully validate our inhibitors. This assay has the advantage that it continuously monitors PARK7 activity, but it remains questionable whether the deacetylation reaction is a correct representation of PARK7 activity. The authors claim that their assay has the potential to be used in HTS, although they do not demonstrate this. We observed a substantial background hydrolysis of the DiFMUAc substrate in phosphate-buffered saline (PBS) in the absence of PARK7, which may bring in additional challenges in the translation of the assay to HTS. Also, the 358/455 nm read-out can result in interference by UV-active small molecules that are highly present in large compound libraries. Nevertheless, we believe that both our FP assay and the DiFMUAc assay will become mutually beneficial and can successfully be applied as orthogonal read-outs in future HTS campaigns.

A potential drawback of our assay is the irreversible nature of the binding between the probe and PARK7 because the probe can displace reversibly bound inhibitors. As a result, the (noncovalent) reversible interactions of any potential PARK7 inhibitor may be missed. On the other hand, there are several examples of similar applications of an irreversible probe in FP assays (often referred to as Fluopol-ABPP) and HTS. These include irreversible probes for serine hydrolase RBBP9, nonlysosomal glucosylceramidase GBA2,^42^ and golgi mannosidase GMII.^43^ A critical parameter is the incubation time because the amount of probe-bound enzyme increases overtime. This is indeed the case in our assay as is revealed by the time dependency of the FP signal (Figure 5 and Supporting Information Figure S6). A solution to this is to choose the conditions as such that upon read-out the enzyme is only partially labeled by the probe.^41^ Based on our data, the selected incubation time of 120 min at 0.2 μM PARK7 concentration is within the kinetically controlled window (while retaining a good assay window and *Z*’ value) and should allow for the identification of both reversible and irreversible inhibitors. Additionally, the IC_50_ value is a suitable indicator of reversible inhibitors for their potential of enzyme inhibition, but for irreversible inhibitors, the inhibition constant (*K*_I_) and the rate of enzyme inactivation (*k*_inact_) values are more suitable parameters. Although it is a complicated issue to calculate *k*_inact_ and *K*_I_ values from a covalent FP assay, a solution to this has been reported by Pettinger et al.^51,52^ for a heat shock protein HSP72 covalent FP assay. Indeed, by using their method, we were able to determine the *k*_inact_/*K*_I_ value for our FP probe **JYQ-107**.

With the installment of a SulfoCy5 dye onto inhibitor **JYQ-88**, we created the first active site-targeting probe for PARK7 (**JYQ-92**) that can be used to label active PARK7 in a cell lysate. This labeling is selective as the probe almost exclusively labels PARK7 at concentrations up to 1 μM. As such, this probe will be a valuable tool to assess the effect of inhibitors on PARK7 activity or to study PARK7 function in a physiologically relevant setting. In principle, Rho110 probe **JYQ-107** could also be used for this as both probes show equal labeling in the in vitro experiment. Here, we focused primarily on SulfoCy5 probe **JYQ-92** because of its better water solubility and the more favorable higher wavelength. Depending on the application, one can choose between either of the probes.

In conclusion, we have developed three novel tools for PARK7: a selective and potent covalent PARK7 inhibitor (**JYQ-88**), an activity-based probe (**JYQ-92**) to monitor PARK7 activity in cell lysates, and a FP assay reagent (**JYQ-107**) to allow for HTS of PARK7 inhibitors. Together, these tools open new avenues to study the biological role of PARK7 activity served by catalytic cysteine rather than its scaffolding function by monitoring PARK7 activity. Future applications of our tools will also pave the way to obtain and validate new and improved PARK7 inhibitors, which could potentially serve as new anticancer drugs. Moreover, with the further innovative improvements, our probes can also serve as a diagnostic tool, as PARK7 is a well-known biomarker for various cancers.^16,53−55^

## Experimental Section

### Chemistry

All reagents and solvents were purchased from commercially available sources and used as received unless indicated otherwise. All reaction progress was monitored by thin-layer chromatography under UV light or by using a solution of KMnO_4_ (7.5 g L^–1^) and K_2_CO_3_ (50 g L^–1^) in H_2_O and liquid chromatography-mass spectrometry (LC–MS). Compounds were purified by Büchi flash column chromatography (unless indicated otherwise) using GraceResolv Davisil silica with indicated eluents. ^1^H and ^13^C nuclear magnetic resonance (NMR) spectra were recorded on a Bruker Avance 300 (300 MHz for ^1^H, 75.00 MHz for ^13^C) using the residual solvents (CDCl_3_ and DMSO-d_6_) as internal references. High-resolution mass spectra were recorded on a Waters Acquity H-class UPLC with a UPLC BEH C18 column (1.7 μm, 2.1 × 50 mm) coupled to a Xevo G2-XS Qtof mass spectrometer with ESI. Optical rotations [α]_D_^25^ of indicated compounds (10 mg/mL in CHCl_3_, *c* = 1) were measured using an Antoton Paar MCP 100 with a 10 cm sample cell. Preparative high-performance liquid chromatography (HPLC) was performed using a Waters preparative automated HPLC instrument with mass detection. Samples were run on a Xbridge PREP C18 column (5 μm, 19 × 150 mm) using base (NH_4_OH) modified CH_3_CN/H_2_O gradients. Gradient: 0–2.5 min: 95% H_2_O, 5% CH_3_CN; 2.5–17.5 min: 5 to 40% CH_3_CN; 17.5–20.90 min: 40 to 95% CH_3_CN; 20.90–21.00 min: 95 to 5% CH_3_CN; 1 mL min^–1^ CH_3_CN (with 1% 4 M NH_4_OH) mixed through the run. All final compounds had a purity ≥95% confirmed by LC–MS and NMR.

#### (9*H*-Fluoren-9-yl)methyl (*S*)-2-(1-(*tert*-Butoxycarbonyl)pyrrolidine-3-carboxamido)-6,7-dihydrothiazolo[5,4-*c*]pyridine-5(4*H*)-carboxylate (**2a**)

The synthesis and characterization of this compound were described previously.^32^ The same sample of compound was used for the current synthesis.

#### (9*H*-Fluoren-9-yl)methyl (*R*)-2-(1-(*tert*-Butoxycarbonyl)pyrrolidine-3-carboxamido)-6,7-dihydrothiazolo[5,4-*c*]pyridine-5(4*H*)-carboxylate (**2b**)

To a solution of (*R*)-1-Boc-1-pyrrolidine-3-carboxylic acid (0.34 g, 1.58 mmol, 1.2 equiv) in dichloromethane (DCM) (20 mL) were added HCTU (0.65 g, 1.58 mmol, 1.2 equiv) and *N,N-*diisopropylethylamine (DiPEA) (0.73 mL, 4.74 mmol, 3.6 equiv), and the reaction mixture was stirred for 10 min. Compound **1** (0.50 g, 1.2 mmol, 1.0 equiv) was then added, and stirring was continued for 2 h at room temperature. The solvents were evaporated under reduced pressure, and the resulting residue was taken up in EtOAc (100 mL). The organic layer was washed with 1 M HCl (2 × 50 mL), sat. aq. NaHCO_3_ (3 × 50 mL), and brine (50 mL), followed by drying with Na_2_SO_4_ and evaporating under reduced pressure. The resulting residue was purified by Büchi flash chromatography (DCM to 4.5% MeOH/DCM) to yield **2b** as a white solid (380 mg, 0.66 mmol, 50%). ^1^H NMR (300 MHz, CDCl_3_) δ 7.69 (d, *J* = 7.2 Hz, 2H), 7.47 (d, *J* = 6.6 Hz, 2H), 7.32 (t, *J* = 7.4 Hz, 2H), 7.23 (t, *J* = 7.5 Hz, 2H), 4.55 (s, 1H), 4.43 (d, *J* = 6.4 Hz, 3H), 4.23–4.14 (m, 1H), 3.74–3.44 (m, 5H), 3.32 (q, *J* = 9.2, 8.6 Hz, 1H), 3.06 (s, 1H), 2.59 (s, 2H), 2.19–2.04 (m, 2H), 1.39 (s, 9H). ^13^C NMR (75 MHz, CDCl_3_) δ 156.4, 155.3, 154.1, 143.8, 141.4, 127.8, 120.0, 79.8, 67.6, 48.3, 47.3, 45.5, 41.6, 28.5. HR-MS calculated for C_31_H_34_N_4_O_5_S [M + H]^+^ 575.2328, found 575.2332.

#### (9*H*-Fluoren-9-yl)methyl (*S*)-2-(1-Cyanopyrrolidine-3-carboxamido)-6,7-dihydrothiazolo[5,4-*c*]pyridine-5(4*H*)-carboxylate (**3a**)

To a solution of **2a** (0.6 g, 1.04 mmol, 1.0 equiv) in DCM (5.0 mL) was added trifluoroacetic acid (TFA) (5.0 mL), and the reaction mixture was stirred for 2 h at room temperature. The solvents were co-evaporated with toluene (3 × 5.0 mL) under reduced pressure. The resulting TFA-salt was used as such. The TFA-salt was dissolved in MeOH (20.0 mL), and NaOAc (0.43 g, 5.2 mmol, 5.0 equiv) and cyanogen bromide (0.44 g, 4.2 mmol, 4.0 equiv) were added. The reaction mixture was stirred overnight at room temperature. The solvents were evaporated under reduced pressure, and the resulting residue was taken up in EtOAc (50 mL). The organic layer was washed with sat. aq. NaHCO_3_ (2 × 25 mL) and brine (25 mL), followed by drying with Na_2_SO_4_ and evaporating under reduced pressure. The resulting residue was purified by Büchi flash chromatography (DCM to 5% MeOH/DCM) to yield **3a** as a white solid (0.40 g, 0.83 mmol, 80%). ^1^H NMR (300 MHz, CDCl_3_) δ 7.76 (s, 2H), 7.56 (s, 2H), 7.40 (t, *J* = 7.5 Hz, 2H), 7.30 (t, *J* = 7.4 Hz, 2H), 4.64 (s, 1H), 4.50 (d, *J* = 6.6 Hz, 3H), 4.27 (t, *J* = 6.5 Hz, 1H), 3.81–3.59 (m, 5H), 3.51 (dt, *J* = 9.4, 7.3 Hz, 1H), 3.21–3.11 (m, 1H), 2.69 (s, 2H), 2.33–2.19 (m, 2H). ^13^C NMR (75 MHz, DMSO-*d*_6_) δ 168.6, 155.3, 155.0, 143.6, 141.1, 127.5, 126.8, 124.6, 119.8, 116.2, 67.4, 52.2, 49.9, 47.1, 44.0, 41.4, 29.3. HR-MS calculated for C_27_H_25_N_5_O_3_S [M + H]^+^ 500.1756, found 500.1770.

#### (9*H*-Fluoren-9-yl)methyl (*R*)-2-(1-Cyanopyrrolidine-3-carboxamido)-6,7-dihydrothiazolo[5,4-*c*]pyridine-5(4*H*)-carboxylate (**3b**)

This compound was prepared according to the procedure for **3a** using compound **2b** (0.37 g, 0.64 mmol, 1.0 equiv) as the starting material. Purification after Büchi flash chromatography (DCM to 5% MeOH/DCM) yielded **3b** as a white solid (0.28 g, 0.56 mmol, 87%). ^1^H NMR (300 MHz, CDCl_3_) δ 7.68 (s, 2H), 7.49 (s, 2H), 7.33 (t, *J* = 7.3 Hz, 2H), 7.23 (t, *J* = 7.3 Hz, 2H), 4.56 (s, 1H), 4.44 (d, *J* = 6.4 Hz, 3H), 4.19 (t, *J* = 6.5 Hz, 1H), 3.74–3.60 (m, 4H), 3.60–3.51 (m, 1H), 3.49–3.38 (m, 1H), 3.20–3.06 (m, 1H), 2.61 (s, 2H), 2.21 (q, *J* = 7.1 Hz, 2H). ^13^C NMR (75 MHz, CDCl_3_) δ 169.0, 156.0, 155.3, 143.8, 141.4, 127.8, 127.1, 124.9, 120.1, 116.6, 67.7, 52.5, 50.2, 47.3, 44.3, 41.5, 29.5. HR-MS calculated for C_27_H_25_N_5_O_3_S [M + H]^+^ 500.1756, found 500.1770.

#### (3*S*)-1-Cyano-*N*-(5-(1-((tetrahydrofuran-2-yl)methyl)-1*H*-1,2,3-triazole-4-carbonyl)-4,5,6,7-tetrahydrothiazolo[5,4-*c*]pyridin-2-yl)pyrrolidine-3-carboxamide (**JYQ-55**)

To a solution of **3a** (50 mg, 100 μmol, 1.0 equiv) in DCM (5.0 mL) was added DBU (6.9 μL, 0.44 mmol, 0.5 equiv), and the reaction mixture was stirred at room temperature for 2 h. After complete removal of the Fmoc group, 1-((tetrahydrofuran-2-yl)methyl)-1*H*-1,2,3-triazole-4-carboxylic acid (23.7 mg, 120 μmol, 1.2 equiv), HCTU (49.6 mg, 120 μmol, 1.2 equiv), and DiPEA (59.4 μL, 360 μmol, 3.6 equiv) were added. The resulting reaction mixture was stirred at room temperature for 2 h, followed by removal of the solvents under reduced pressure. The crude material was taken up in EtOAc (20 mL), and the organic layer was washed with 1 M HCl (2 × 10 mL), sat. aq. NaHCO_3_ (3 × 10 mL), and brine (10 mL). The organic layer was dried over Na_2_SO_4_ and evaporated under reduced pressure. The resulting residue was purified by Büchi flash chromatography (DCM to 4.5% MeOH/DCM) to yield **2a** as a white solid (18.25 mg, 40 μmol, 40%). ^1^H NMR (300 MHz, DMSO-*d*_6_) δ 12.24 (s, 1H), 8.52 (s, 1H), 5.22 (s, 1H), 4.78 (s, 1H), 4.52 (s, 1H), 4.44 (s, 1H), 4.32–4.18 (m, 2H), 3.96 (s, 1H), 3.79–3.71 (m, 1H), 3.70–3.57 (m, 2H), 3.56–3.38 (m, 4H), 2.85–2.70 (m, 2H), 2.23–2.14 (m, 1H), 2.02 (ddd, *J* = 13.1, 9.8, 5.9 Hz, 2H), 1.79 (dq, *J* = 13.2, 6.0 Hz, 2H), 1.63 (dt, *J* = 12.5, 7.3 Hz, 1H). ^13^C NMR (75 MHz, DMSO-*d*_6_) δ 170.8, 160.6, 156.5, 143.6, 143.1, 129.7, 118.9, 118.8, 77.0, 67.9, 53.8, 52.6, 50.4, 44.5, 43.5, 29.7, 28.7, 27.8, 26.4, 25.4. HR-MS calculated for C_20_H_24_N_8_O_3_S [M + H]^+^ 457.1770, found 457.1777.

#### (*S*)-1-Cyano-*N*-(5-(2-(pyridin-4-yl)acetyl)-4,5,6,7-tetrahydrothiazolo[5,4-*c*]pyridin-2-yl)pyrrolidine-3-carboxamide (**JYQ-76**)

This compound was prepared according to the procedure for **JYQ-55** using 2-(pyridin-4-yl)acetic acid (16.5 mg, 120 μmol, 1.2 equiv) as the starting material. Purification after Büchi flash chromatography (DCM to 5% MeOH/DCM) yielded **JYQ-76** as a white solid (19.8 mg, 41 μmol, 41%). ^1^H NMR (300 MHz, DMSO-*d*_6_) δ 12.23 (s, 1H), 8.44 (dd, *J* = 7.9, 2.5 Hz, 2H), 7.63 (d, *J* = 2.2 Hz, 1H), 7.40–7.29 (m, 1H), 4.77 (s, 1H), 4.64 (s, 1H), 3.93–3.79 (m, 4H), 3.65–3.48 (m, 4H), 2.69 (t, *J* = 15.0 Hz, 3H), 2.20–2.00 (m, 2H).^13^C NMR (75 MHz, DMSO-*d*_6_) δ 170.9, 169.3, 156.6, 150.9, 148.0, 143.2, 137.5, 132.1, 123.7, 119.1, 117.5, 52.6, 50.4, 43.6, 43.3, 40.6, 37.3, 36.9, 29.7, 27.3. HR-MS calculated for C_19_H_20_N_6_O_2_S [M + H]^+^ 397.1447, found 397.1443.

#### (*S*)-*N*-(5-(4-Bromobenzoyl)-4,5,6,7-tetrahydrothiazolo[5,4-*c*]pyridin-2-yl)-1-cyanopyrrolidine-3-carboxamide (**JYQ-77**)

This compound was prepared according to the procedure for **JYQ-55** using 4-bromobenzoic acid (14.5 mg, 72 μmol, 1.2 equiv) as the starting material. Purification after Büchi flash chromatography (DCM to 5% MeOH/DCM) yielded **JYQ-77** as a white solid (9.8 mg, 21.6 μmol, 36%). ^1^H NMR (300 MHz, DMSO-*d*_6_) δ 12.26 (s, 1H), 7.68 (dd, *J* = 8.4, 4.1 Hz, 2H), 7.49–7.34 (m, 2H), 4.75 (s, 1H), 4.57 (s, 1H), 3.93 (s, 1H), 3.62 (t, *J* = 8.8 Hz, 2H), 3.56–3.37 (m, 4H), 2.79–2.67 (m, 2H), 2.22–2.02 (m, 2H).^13^C NMR (75 MHz, DMSO-*d*_6_) δ 170.8, 169.1, 156.7, 143.3, 135.5, 132.0, 129.6, 123.7, 118.6, 117.5, 52.6, 50.4, 45.0, 43.5, 42.3, 29.6, 27.1. HR-MS calculated for C_19_H_18_BrN_5_O_2_S [M + H]^+^ 460.0443 found 460.0440.

#### *(S)*-*N*-(5-(5-Bromopentanoyl)-4,5,6,7-tetrahydrothiazolo[5,4-*c*]pyridin-2-yl)-1-cyanopyrrolidine-3-carboxamide (**JYQ-78**)

This compound was prepared according to the procedure for **JYQ-55** using 5-bromopentanoic acid (13.0 mg, 72 μmol, 1.2 equiv) as the starting material. Purification after Büchi flash chromatography (DCM to 5% MeOH/DCM) yielded **JYQ-78** as a white solid (9.2 mg, 21 μmol, 35%). ^1^H NMR (300 MHz, DMSO-*d*_6_) δ 12.23 (d, *J* = 5.2 Hz, 1H), 4.63 (d, *J* = 13.4 Hz, 2H), 3.75 (s, 2H), 3.65–3.50 (m, 4H), 3.42 (td, *J* = 6.9, 2.3 Hz, 3H), 2.80–2.54 (m, 3H), 2.25–1.99 (m, 2H), 1.90–1.58 (m, 5H). ^13^C NMR (75 MHz, DMSO-*d*_6_) δ 13C NMR (75 MHz, DMSO-d6) δ 171.20, 157.53, 141.98, 117.53, 114.94, 52.54, 50.38, 42.90, 41.64, 35.44, 29.88, 29.61, 23.84, 22.15. HR-MS calculated for C_17_H_22_BrN_5_O_2_S [M + H]^+^ 440.0756, found 440.0752.

#### (3*S*)-1-Cyano-*N*-(5-((2*R*)-1-methyl-5-oxo-2-(pyridin-3-yl)pyrrolidine-3-carbonyl)-4,5,6,7-tetrahydrothiazolo[5,4-*c*]pyridin-2-yl)pyrrolidine-3-carboxamide (**JYQ-79**)

This compound was prepared according to the procedure for **JYQ-55** using (2*R*)-1-methyl-5-oxo-2-(pyridin-3-yl)pyrrolidine-3-carboxylic acid (15.8 mg, 72 μmol, 1.2 equiv) as the starting material. Purification after Büchi flash chromatography (DCM to 5% MeOH/DCM) yielded **JYQ-79** as a white solid (13.6 mg, 23.4 μmol, 39%). ^1^H NMR (300 MHz, DMSO-*d*_6_) δ 12.22 (d, *J* = 6.2 Hz, 1H), 8.62–8.47 (m, 2H), 7.75 (ddt, *J* = 21.4, 8.1, 2.1 Hz, 1H), 7.46 (td, *J* = 7.6, 3.9 Hz, 1H), 4.92 (t, *J* = 5.7 Hz, 1H), 4.63 (s, 1H), 4.52 (d, *J* = 14.9 Hz, 1H), 3.75–3.55 (m, 4H), 3.54–3.39 (m, 4H), 2.88 (ddd, *J* = 15.0, 9.7, 4.8 Hz, 1H), 2.75–2.56 (m, 3H), 2.41–2.27 (m, 2H), 2.18 (dd, *J* = 13.0, 6.5 Hz, 1H), 2.03 (dd, *J* = 13.0, 6.8 Hz, 1H).^13^C NMR (75 MHz, DMSO-*d*_6_) δ 172.5, 172.3, 170.8, 170.6, 156.4, 150.0, 149.9, 149.4, 149.2, 143.8, 143.0, 135.8, 135.5, 135.4, 124.6, 124.5, 118.8, 118.4, 117.5, 64.1, 63.9, 54.1, 52.5, 50.4, 43.5, 43.3, 43.1, 43.0, 42.9, 34.8, 34.4, 29.6, 28.2, 27.4, 26.3. HR-MS calculated for C_23_H_25_N_7_O_3_S [M + H]^+^ 480.1818, found 480.1810.

#### (3*S*)-*N*-(5-((1*S*,2*R*,5*R*)-Adamantane-2-carbonyl)-4,5,6,7-tetrahydrothiazolo[5,4-*c*]pyridin-2-yl)-1-cyanopyrrolidine-3-carboxamide (**JYQ-83**)

This compound was prepared according to the procedure for **JYQ-55** using 2-adamantanecarboxylic acid (21.6 mg, 120 μmol, 1.2 equiv) as the starting material. Purification after Büchi flash chromatography (DCM to 5% MeOH/DCM) yielded **JYQ-83** as a white solid (19.4 mg, 44 μmol, 44%). ^1^H NMR (300 MHz, DMSO-*d*_6_) δ 12.21 (s, 1H), 4.71 (s, 2H), 3.94–3.82 (m, 2H), 3.66–3.56 (m, 1H), 3.56–3.47 (m, 1H), 3.47–3.38 (m, 3H), 2.74–2.64 (m, 2H), 2.28 (q, *J* = 1.8 Hz, 1H), 2.19–2.05 (m, 2H), 1.96 (d, *J* = 19.0 Hz, 8H), 1.70 (d, *J* = 8.3 Hz, 6H). ^13^C NMR (75 MHz, DMSO-*d*_6_) δ 175.5, 170.9, 156.7, 143.6, 119.4, 117.6, 52.6, 50.4, 43.6, 43.5, 43.0, 41.7, 38.9, 36.5, 29.7, 28.4, 27.2. HR-MS calculated for C_23_H_29_N_5_O_2_S [M + H]^+^ 440.2120, found 440.2127.

#### (*R*)-*N*-(5-(2-Azidoacetyl)-4,5,6,7-tetrahydrothiazolo[5,4-*c*]pyridin-2-yl)-1-cyanopyrrolidine-3carboxamide (**JYQ-88**)

This compound was prepared according to the procedure for **JYQ-55** using **3b** (100 mg, 200 μmol, 1.0 equiv) and azidoacetic acid (21.6 mg, 240 μmol, 1.2 equiv) as the starting materials. Purification after Büchi flash chromatography (DCM to 5% MeOH/DCM) yielded **JYQ-88** as a white solid (18.8 mg, 52 μmol, 26%). [α]_D_^25^-10.13°. (**8RK64**: [α]_D_^25^+10.13°). ^1^H NMR (300 MHz, CDCl_3_) δ 4.85–4.77 (m, 1H), 4.58 (s, 1H), 4.09 (d, *J* = 11.6 Hz, 2H), 3.98 (t, *J* = 5.6 Hz, 1H), 3.73 (td, *J* = 8.1, 7.6, 2.2 Hz, 3H), 3.69–3.61 (m, 1H), 3.60–3.49 (m, 1H), 3.38–3.24 (m, 1H), 2.82 (d, *J* = 23.0 Hz, 2H), 2.31 (q, *J* = 7.1 Hz, 2H). ^13^C NMR (75 MHz, CDCl_3_) δ 169.2, 166.3, 156.5, 141.3, 119.8, 116.7, 52.6, 51.2, 50.2, 44.2, 42.7, 40.2, 29.4, 27.0. HR-MS calculated for C_14_H_16_N_8_O_2_S [M + H]^+^ 361.1195, found 361.1190.

#### SulfoCy5 Probe **JYQ-92**

A solution of compound **JYQ-88** (10.0 mg, 27.28 μmol, 1.0 equiv) and SulfoCy5 alkyne (24.0 mg, 33.33 μmol, 1.2 equiv) in dry DMF (2.0 mL) was degassed by argon for 30 min. Aqueous solutions of sodium ascorbate (0.5 M) and CuSO_4_·5H_2_O (0.5 M) were prepared in 1.0 mL volume and degassed for 30 min with argon bubbling. The degassed sodium ascorbate (83 μL, 41.66 μmol, 1.2 equiv) and CuSO_4_·5H_2_O (69 μL, 34.7 μmol, 1.0 equiv) solutions were added to the reaction mixture, followed by stirring for 2 h. The resulting crude material was purified by preparative HPLC to yield **JYQ-92** as a blue solid (5.9 mg, 5.5 μmol, 20%). ^1^H NMR (300 MHz, DMSO-*d*_6_) δ 12.26 (d, *J* = 9.6 Hz, 1H), 8.35 (t, *J* = 13.1 Hz, 2H), 7.85 (t, *J* = 5.6 Hz, 1H), 7.80 (t, *J* = 1.9 Hz, 2H), 7.77 (d, *J* = 3.1 Hz, 1H), 7.64 (ddd, *J* = 7.6, 6.0, 1.5 Hz, 2H), 7.32 (dd, *J* = 8.4, 2.7 Hz, 2H), 7.29 (s, 1H), 7.12 (s, 1H), 6.95 (s, 1H), 6.58 (t, *J* = 12.3 Hz, 1H), 6.36–6.25 (m, 2H), 5.54 (s, 1H), 5.47 (s, 1H), 4.75 (s, 1H), 4.63 (s, 1H), 4.19–4.02 (m, 4H), 3.81 (d, *J* = 6.7 Hz, 2H), 3.65–3.54 (m, 3H), 3.38–3.29 (m, 2H), 3.06 (q, *J* = 6.6 Hz, 2H), 2.81 (s, 1H), 2.68–2.53 (m, 3H), 2.26–1.97 (m, 4H), 1.68 (s, 12H), 1.54 (t, *J* = 7.3 Hz, 2H), 1.32 (s, 2H), 1.25 (t, *J* = 7.1 Hz, 3H). ^13^C NMR (75 MHz, DMSO-*d*_6_) δ 173.5, 173.1, 172.4, 171.0, 170.9, 165.5, 165.4, 156.2, 154.8, 154.7, 145.5, 145.4, 143.2, 142.5, 142.0, 141.1, 141.0, 139.0, 126.6, 126.3, 120.4, 118.6, 118.3, 117.6, 110.7, 110.4, 103.9, 103.6, 52.5, 51.0, 50.4, 49.4, 43.5, 42.3, 38.4, 35.6, 29.7, 29.5, 27.5, 27.1, 26.1, 25.4, 23.0, 12.5. HR-MS calculated for C_52_H_62_N_11_O_9_S_3_ [M + H]^2+^ 541.7064, found 541.7066.

#### Bodipy Probe **JYQ-93**

This compound was prepared according to the procedure of **JYQ-92** using Bodipy alkyne (13.7 mg, 41.6 μmol, 1.2 equiv) as the starting material. Purification by preparative HPLC yielded **JYQ-93** as a red solid (4.3 mg, 6.24 μmol, 18%). ^1^H NMR (300 MHz, DMSO-*d*_6_) δ 12.26 (s, 1H), 7.77 (d, *J* = 1.6 Hz, 1H), 6.23 (s, 2H), 5.77 (s, 1H), 5.50 (d, *J* = 20.0 Hz, 2H), 4.70 (d, *J* = 36.3 Hz, 2H), 3.82 (d, *J* = 5.5 Hz, 2H), 3.67–3.47 (m, 3H), 2.98 (t, *J* = 8.5 Hz, 2H), 2.74–2.66 (m, 4H), 2.40 (s, 6H), 2.25 (d, *J* = 1.4 Hz, 3H), 2.17 (d, *J* = 1.5 Hz, 3H), 1.83 (t, *J* = 7.6 Hz, 2H), 1.68–1.59 (m, 3H), 1.50 (dd, *J* = 8.2, 6.3 Hz, 2H).^13^C NMR (75 MHz, DMSO-*d*_6_) δ 170.9, 165.3, 153.5, 147.2, 146.6, 143.2, 141.4, 134.0, 131.2, 128.0, 124.2, 122.1, 118.6, 117.5, 112.3, 71.7, 52.6, 51.1, 50.4, 43.5, 42.6,42.3, 38.4, 31.3, 29.9, 29.7, 28.2, 28.1, 25.1, 23.8, 18.1, 16.3, 14.5, 13.0. HR-MS calculated for C_33_H_39_BF_2_N_10_O_2_S [M + H]^+^ 689.3123, found 689.3123.

#### Rhodamine110 Probe **JYQ-107**

This compound was prepared according to the procedure of **JYQ-92** using Rhodamine alkyne (13.03 mg, 72 μmol, 1.2 equiv).^31^ Purification by preparative HPLC yielded **JYQ-107** as a red solid (9.8 mg, 12.3 μmol, 36%). ^1^H NMR (300 MHz, DMSO-*d*_6_) δ 12.27 (d, *J* = 10.3 Hz, 1H), 8.88 (t, *J* = 5.6 Hz, 1H), 8.44 (s, 1H), 8.24 (dd, *J* = 8.0, 1.6 Hz, 1H), 7.82 (d, *J* = 2.9 Hz, 1H), 7.33 (d, *J* = 8.0 Hz, 1H), 6.44–6.22 (m, 6H), 5.73–5.49 (m, 5H), 4.71 (d, *J* = 36.9 Hz, 2H), 3.83 (d, *J* = 5.0 Hz, 2H), 3.66–3.58 (m, 1H), 3.58–3.39 (m, 5H), 2.82 (s, 1H), 2.78–2.65 (m, 3H), 2.25–2.12 (m, 1H), 2.12–1.96 (m, 1H), 1.96–1.81 (m, 2H). HR-MS calculated for C_40_H_37_N_11_O_6_S [M + H]^+^ 800.2727, found 800.2723.

#### STK793590

To a solution of 5-fluoroindoline-2,3-dione (50 mg, 0.30 mmol, 1.0 equiv) in DMF (3 mL) were added 1-phenyl-2-bromoethane (67.25 mg, 0.36 mmol, 1.2 equiv) and K_2_CO_3_ (50.22 mg, 0.36 mmol, 1.2 equiv). The reaction mixture was stirred overnight at room temperature. The solvents were evaporated under reduced pressure, and the resulting residue was taken up in EtOAc (20 mL). The organic layer was washed with water, followed by drying with Na_2_SO_4_ and evaporating under reduced pressure. The resulting residue was purified by Büchi flash chromatography (heptane to 25% heptane/EtOAc) to obtain **STK793590** as a red solid (73.38 mg, 0.27 mmol, 90%). ^1^H NMR (300 MHz, DMSO-*d*_6_) δ 7.54–7.42 (m, 2H), 7.28 (d, *J* = 4.4 Hz, 4H), 7.20 (td, *J* = 8.7, 3.7 Hz, 2H), 3.94–3.86 (m, 2H), 2.96–2.87 (m, 2H). ^13^C NMR (75 MHz, DMSO-*d*_6_) δ 183.26, 160.45, 157.26, 147.27, 138.63, 129.35, 128.89, 126.96, 124.72, 124.40, 118.59, 112.83, 112.09,111.77 41.55, 33.11.

#### DiFMUAc Assay Reagent

To a solution of 2,4-difluorobenzene-1,3-diol (300 mg, 2.05 mmol, 1.0 equiv) in CH_3_SO_3_H at 0 °C was added ethyl 3-oxobutanoate (280 mg, 2.05 mmol, 1 equiv). The reaction was stirred for 4 h at room temperature. The reaction was diluted with water and extracted with EtOAc (3×). The combined organic layer was evaporated under reduced pressure. The resulting residue was directly used in the next step.

To a solution of 6,8-difluoro-7-hydroxy-4-methyl-chromen-2-one (220 mg, 1.03 mmol, 1 equiv) in pyridine (5 mL) was added Ac_2_O (158 mg, 1.55 mmol, 1.5 equiv). The reaction was heated to 60 °C and stirred overnight. The reaction was concentrated in vacuo, and the resulting residue was purified by Büchi flash chromatography to obtain DiFMUAc (heptane to 25% heptane/EtOAc) as a white solid (128.2 mg, 0.50 mmol, 49%). ^1^H NMR (300 MHz, CDCl_3_) δ 7.22 (dd, *J* = 9.7, 2.3 Hz, 1H), 6.42–6.35 (m, 1H), 2.45 (s, 3H), 2.44 (d, *J* = 1.3 Hz, 3H). ^13^C NMR (75 MHz, CDCl_3_) δ 166.79, 158.47, 152.62, 150.94, 149.29, 144.78, 116.41, 105.81, 105.47, 20.07, 18.83.

### Protein Expression and Purification

The coding DNA for PARK7 (or DJ-1; Uniprot: Q99497) was a kind gift from Koraljka Husnjak and was cloned into the in-house vector containing a 3C protease-cleavable N-terminal GST-tag, using LIC cloning.^56^ The active site mutant (Cys106Ser, site-directed mutagenesis) was subsequently generated using IVA cloning.^57^ All expression constructs were sequence-verified and are available upon request.

The PARK7 constructs were expressed using BL21(DE3) Rosetta2 cells, with ampicillin (100 μg mL^–1^) and chloramphenicol (37 μg mL^–1^) as antibiotic selection. An overnight culture was diluted into 1 L LB and grown at 37 °C until the OD_600_ reached 0.6. Expression was then induced using 0.2 mM IPTG for 3 h at 20 °C. Cells were harvested by centrifugation (20′ at 4000 G), resuspended in GST buffer (50 mM HEPES pH 7.5, 250 mM NaCl, 1 mM EDTA, 1 mM DTT), and frozen at −20 °C until further processing.

All used PARK7 proteins were purified in a similar manner and at 4 °C. The expressing cells were lysed using sonication, and cell debris was removed using 40 min of centrifugation at 21,000 G. The supernatant was loaded on Glutathione Sepharose 4B beads pre-equilibrated with GST buffer. After extensive washing with GST buffer, the protein was eluted with this buffer, supplemented with 25 mM glutathione. Elution fractions were analyzed on SDS-PAGE, and PARK7-containing fractions were combined for overnight cleavage with GST-tagged 3C protease under dialysis against SEC buffer (20 mM HEPES pH 7.5, 150 mM NaCl 1 mM DTT). The next day, the dialysate was concentrated to <5 mL and injected on a Superdex75 16/60 column (Cytiva) connected to a GSTrap (Cytiva) and equilibrated in SEC buffer using an NGC FPLC (Bio-rad). Peak fractions were analyzed on gel, and pure fractions were combined, concentrated, and flash frozen in liquid nitrogen for storage at −80 °C.

### Crystallization of PARK7 and Data Collection

Prior to crystallization, a covalent complex of PARK7 WT and an inhibitor was generated through incubation of the protein with the inhibitor in 1.5 molar excess. The reaction was done in xTal buffer (20 mM HEPES pH 7.0, 50 mM NaCl), supplemented with 10 mM TCEP using ∼250 μM protein. After 2 h, conversion was checked for >90% conversion using mass spectrometry and the mix was injected on the Superdex75 10/300 column using xTal buffer to remove the unreacted inhibitor. The elution fractions corresponding to the single protein peak were concentrated to 20 mg mL^–1^ and set up for crystallization with 0.1 μL of protein solution and 0.1 μL of mother liquor using nanoliter dispensing robot NT8 (Formulatrix). Specific crystallization conditions for each PARK7 complex are reported below.

Crystals appeared within days in several conditions of the JCSG screen (molecular dimensions) and were left until they stopped growing. Single crystals were harvested in mother liquor supplemented with 30% ethylene glycol as a cryoprotectant and flash frozen in liquid nitrogen. Crystals were shipped to Diamond Light Source (DLS; United Kingdom), and diffraction data were recorded on beamlines i24 and i04.

### Structure Determination

All crystal datasets were processed using Dials on the DLS computing grid. A selection was made by ranking their diffraction resolution and completeness using AIMLESS within the CCP4 program suite,^58−60^ before solving the structures of the top five by molecular replacement with PDB 6M8Z using PHASER.^61^ Of these, the datasets containing proper electron density for an inhibitor in the binding pocket were selected for refinement. In Table S1, we only report the statistics for the one structure with most details for each inhibitor.

After solving with PHASER, each structure model was refined by rounds of rebuilding with COOT and restrained refinement in REFMAC,^62,63^ before being passed through the PDB REDO pipeline.^64^ Restraints for the inhibitors were generated using the GRADE webserver (Global Phasing Ltd.), where possible placed inside the resulting density and passed through further (water) building steps in COOT and anisotropic refinement using REFMAC. The resulting statistics are presented in Table S1, and the final models are deposited in the Protein Data Bank under accession codes 7PA2 and 7PA3 (**8RK64** and **JYQ-88**, respectively). Both PyMol and CCP4MG were used to generate structure model figures.

### Probe Labeling of Purified Recombinant PARK7 and UCHL1

All the labeling assays were performed in Tris buffer (50 mM Tris–HCl, 150 mM NaCl, 2 mM TCEP, pH 7.5). For labeling with the **8RK59** probe, 1 μM final concentrations of UCHL1 and PARK7 were individually or together incubated with 2 μM final concentrations of **8RK59** for 30 min at 37 °C, followed by protein visualization. For labeling with **JYQ-92**, **JYQ-93**, and **JYQ-107**, 1 μM final concentration of PARK7 was incubated with different concentrations (0, 0.1, 0.5, 1, 2, 5, and 10 μM) of the indicated probes for 1 h at 37 °C. After completing the incubation time, all of the reactions were stopped by adding NuPAGE LDS sample buffer (4×). These samples were resolved by SDS-PAGE using precast Bis-Tris gels (Invitrogen, NuPAGE) with MES SDS running buffer (Novex, NuPAGE). The gels were visualized by Typhoon FLA 9500 (GE Healthcare Life Sciences) fluorescence scanning: PARK7–**8RK59**, UCHL1–**8RK59** and PARK7–**JYQ-93**, PARK7**–JYQ-107** adducts were visualized with a Rhodamine channel (λ_ex/em_ 473/530 nm); PARK7–**JYQ-92** adducts were visualized with a Cy5 channel (λ_ex/em_ 635/655 nm), followed by staining with InstantBlue Coomassie protein stain (Expedeon) and scanning on an Amersham Imager 600 (GE Healthcare Life Sciences).

### Gel-Based Competition Assay

The assay was performed in Tris buffer as described above. The mixture of 1 μM final concentrations of UCHL1 and PARK7 was treated with 2 μM final concentration of indicated compounds and incubated for 1 h at 37 °C, followed by incubation with 2 μM final concentration of **8RK59** for 30 min at 37 °C, and protein labeling was visualized as described above.

### IC_50_ Determination of **JYQ-88** and **STK793590** for PARK7 by the DiFMUAc Assay

The assay was performed in the PBS buffer and conducted in a 384-well plate (Corning 3820) with a reaction volume of 20 μL in triplicate. Stock solutions of compounds of 0.01, 0.1, and 1 mM were prepared. Compounds were transferred using a Labcyte Echo550 acoustic dispenser to obtain a 12-point serial dilution of 0.001 to 100 μM. Next, PARK7 (15 μL, final concentration of 0.1 μM) was dispensed with a Biotek MultiFlowFX liquid dispenser and incubated for 1 h, followed by dispensing the substrate DiFMUAc (5 μL, final concentration of 300 μM). The fluorescence intensity (FI) signal was monitored with a BMG Labtech PHERAstar plate reader (λ_ex/em_ 350/450 nm) for 1 h. All samples were normalized to the positive and negative controls and plotted against the inhibitor concentrations (in μM) using the built-in equation “[inhibitor] vs response – variable slope (four parameters), least-squares fit” with constraints “Bottom = 0” and “Top = 100” in GraphPad Prism 9.0.1 software to obtain the IC_50_ values.

### IC_50_ Determination for UCHL1

The assay was performed in a buffer containing 50 mM Tris·HCl, 100 mM NaCl at pH 7.5, 2.0 mM cysteine, 1 mg/mL 3-[(3-cholamidopropyl)dimethylammonio]propanesulfonic acid (CHAPS), and 0.5 mg/mL bovine serum albumin and conducted in a 384-well plate (Corning 3820) with a reaction volume 20 μL in triplicate. The compounds were dissolved in DMSO as 10, 1, and 0.1 mM stock solutions and were transferred to obtain a 12-point serial dilution of 0.05 to 200 μM. Next, UCHL1 (15 μL, final concentration of 1 nM) was dispensed with a Biotek MultiFlowFX liquid dispenser and incubated for 1 h, followed by dispensing the substrate Ub-Rho-Morpholine or Ub-AMC (5 μL, final concentration of 400 nM). The FI signal was monitored with a BMG Labtech PHERAstar plate reader (λ_ex/em_ 487/535 nm or λ_ex/em_ 350/450 nm) for 1 h. N-Ethylmaleimide (NEM, 10 mM) was used as a positive control (100% inhibition), and DMSO was used as a negative control (0% inhibition). All samples were normalized to the positive and negative controls and plotted against the inhibitor concentrations (in μM) using the built-in equation “[inhibitor] vs response – variable slope (four parameters), least-squares fit” with constraints “Bottom = 0” and “Top = 100” in GraphPad Prism 9.0.1 software to obtain the IC_50_ values.

### Cell Lines and Cell Culture

HEK293T cells were originally obtained from the American Type Culture Collection (Cat# ATCC CRL-3216) and cultured in Dulbecco’s modified Eagles’medium (Gibco) supplemented with 10% fetal bovine serum at 37 °C and 5% CO_2_.

### siRNA Transfection

For siRNA transfections, oligos used to knockdown PARK7 were purchased from Dharmacon (Cat#: MQ-005984-00-0002). Silencing was performed in HEK293T cells as follows: for a 6-well plate format, 200 μL of siRNA (500 nM stock) was incubated with 4 μL of Dharmafect reagent 1 (Dharmacon) diluted in 200 μL of medium without supplements (total volume of 400 μL of transfection mix) with gentle shaking for 20 min at room temperature (RT). A total of 96 × 10^3^ Hek293T cells resuspended in 1.6 mL of growth medium without antibiotics from 60 × 10^3^ cells per mL suspension were added to transfection mixes to a total volume of 2 mL per well and cultured for 3 days prior to further analysis.

### Antibodies

The following antibodies were used for detection of endogenous protein by Western blot analysis in a 1:1000 dilution: rabbit anti-PARK7 (Abcam, Cat# ab18257) and rabbit anti-UCHL1(Abcam, Cat# ab27053). Mouse anti-β-actin (Sigma-Aldrich, Cat# A5441) was used as a loading control in a 1:10,000 dilution for the Western blot analysis. Secondary IRDye 800CW goat anti-rabbit IgG (H + L) (Li-COR, Cat# 926-32211, 1:5000) and IRDye 680LT goat anti-mouse IgG (H + L) (Li-COR, Cat# 926-68020, 1:20,000) were used for detection using an Odyssey Classic imager (LI-COR).

### Immunoblotting

After proteins were transferred to a nitrocellulose membrane at 300 mA for 2.5 h, the membranes were blocked with 5% milk in PBS and incubated with primary antibody diluted in 5% milk in 0.1% PBS-Tween 20 (PBST) for 1 h at RT. After washing with 0.1% PBST three times for 10 min, proteins were incubated with secondary antibodies diluted in 0.1% PBST for 30 min and washed three times again in 0.1% PBST. The signal was detected using direct imaging using an Odyssey Classic imager (LI-COR).

### Probe **JYQ-92** Labeling of Endogenous PARK7 in the Cell Lysate

HEK293T cell pellets were suspended in the lysis buffer (50 mM Tris,150 mM NaCl, 0.5% Triton X-100, and 2 mM TCEP at pH 7.5) supplemented with protease inhibitor cocktail (11836145001, Roche). The samples were kept on ice and sonicated using 10 cycles of 30 s pulse on and 30 s pulse off (Bioruptor, Diagenode). The cell lysate was centrifuged at 14,000 rpm with an Eppendorf Centrifuge 5430 R for 20 min at 4 °C, and supernatant fractions were collected. For the labeling experiments, the prepared cell lysate was incubated with an indicated concentration of **JYQ-92** in each experiment for 1 h at 37 °C. The reactions were stopped by adding NuPAGE LDS sample buffer (4×). Samples were resolved by SDS-PAGE using a 12% Bis-Tris gel with MES SDS running buffer and visualized by fluorescence scanning with a Typhoon FLA 9500 using a Cy5 channel (λ_ex/em_ 635/655 nm), followed by transferring to nitrocellulose membranes and Western blot analysis. For the gel-based competition assay in the cell lysate, HEK293T cells were harvested and lysed as described above. The prepared cell lysate was incubated with a dilution series of **JYQ-88** (0, 0.1, 0.5, 1, 2, 5, 10, and 20 μM) for 1 h at 37 °C, followed by incubation with 1 μM final concentration of **JYQ-92** for 30 min at 37 °C. Cell lysate labeling was visualized as described above.

### FP Assay Binding Saturation Experiment

This assay was performed in the buffer containing 50 mM Tris–HCl, 150 mM NaCl, 2 mM TCEP, pH 7.5, and 1 mg/mL CHAPS and conducted in a 384-well plate (Corning 3820) with a reaction volume 20 μL in triplicate. A serial dilution of PARK7 from 0 to 5 μM (final concentration) was mixed with constant concentration **JYQ-107** (0.2 μM), and the increasing FP signal over time was measured at λ_ex/em_ 480/520 nm using a PHERAstar (BMG Labtech) for 150 min. The FP signal at 30, 60, 90, 120, and 150 min was plotted against concentration.

### IC_50_ Determination of **JYQ-88** for PARK7 by the FP Assay

The assay was performed using the conditions as described above with a reaction volume of 20 μL per well in triplicate. Stock solutions of **JYQ-88** of 1 and 0.1 mM were prepared. **JYQ-88** was transferred to an empty 384-well plate (Corning 3820) using a Labcyte Echo550 acoustic dispenser to obtain an 8-point serial dilution of 0.1 to 10 μM. Next, PARK7 (15 μL, final concentration of 0.2 μM) was dispensed with a Biotek MultiFlowFX liquid dispenser and incubated for 1 h, followed by dispensing the probe **JYQ-107** (5 μL, final concentration of 20 nM). The FP signal was monitored with a BMG Labtech PHERAstar plate reader (λ_ex/em_ 480/520 nm) for 2 h. All samples were normalized to the positive and negative controls and plotted against the inhibitor concentrations (in μM) using the built-in equation “[inhibitor] vs response – variable slope (four parameters), least-squares fit” with constraints “Bottom = 0” and “Top = 100” in GraphPad Prism 9.0.1 software to obtain the IC_50_ values.

### High-Throughput Screening

The screen was conducted using the conditions as described above in 1536-well plates (Corning 3724) with a reaction volume of 8 μL per well. The compound library containing 7887 covalent fragments was purchased at Enamine Ltd. and received as 100 mM DMSO stocks in 384 well LDV Echo plates (Labcyte LP-0200). Stock solutions of 0.26 μM PARK7 and 80 nM **JYQ-107** were prepared. Using a Labcyte Echo550 acoustic dispenser, 8 nL of the DMSO stock solution of the library compounds was transferred from the source plates into the empty 1536-well screening plates to obtain a 100 μM final compound concentration. Next, PARK7 (6 μL, final concentration 0.2 μM) was dispensed using a Biotek MultiflowFX liquid dispenser and incubated for 2 h, followed by dispensing the probe **JYQ-107** (2 μL, final concentration 20 nM). After 2 h incubation, the FP signal was recorded on a BMG Labtech PHERAstar plate reader (λ_ex/em_ 480/520 nm). The relative loss of FP signal compared with reference controls was used to calculate the remaining enzyme activity.

### Hit Picking and Validation

The validation was performed using the conditions as described above with a reaction volume of 20 μL per well in triplicate. Forty compounds were cherry-picked from 150 hits and tested at three different concentrations. Using a Labcyte Echo550 acoustic dispenser, 20,7.5, and 2.5 nL of the 100 mM DMSO stock solution of the compounds were transferred from the source plates into the empty 384-well plate to obtain 100, 37.5, and 12.5 μM final concentrations. Next, PARK7 (15 μL, final concentration 0.2 μM) was dispensed with a Biotek MultiFlowFX liquid dispenser and incubated for 1 h, followed by dispensing the probe **JYQ-107** (5 μL, final concentration 20 nM). The FP signal was monitored with a BMG Labtech PHERAstar plate reader (λ_ex/em_ 480/520 nm) for 2 h. The relative loss of FP signal compared with reference controls was used to calculate the remaining enzyme activity. Inhibition percentage of these 40 compounds was plotted as a heatmap using GraphPad Prism 9.0.1 software. Color gradients indicate inhibition percentage, no inhibiton (white) and full inhibition (blue).

### Hit Validation by the DiFMUAc Assay

The validation was performed in the PBS buffer with a reaction volume of 20 μL per well in triplicate. The best 22 compounds from hit validation by the FP assay were tested at three different concentrations. The compounds were transferred from source plates into the empty 384-well plate (Corning 3820) to obtain 100, 37.5, and 12.5 μM final concentrations as described above. Next, PARK7 (15 μL, final concentration 0.1 μM) was dispensed with a Biotek MultiFlowFX liquid dispenser and incubated for 2 h, followed by dispensing the substrate DiFMUAc (5 μL, final concentration 300 μM). The FI signal was monitored with a BMG Labtech PHERAstar plate reader (λ_ex/em_ 350/450 nm) for 1 h. The increase of FI signal compared with reference controls was used to calculate the remaining enzyme activity. The inhibition percentage of these 22 compounds was plotted as a heatmap using GraphPad Prism 9.0.1 software. Color gradients indicate inhibition percentage, no inhibiton (white) and full inhibition (blue).

### Hit Validation in the Cell Lysate

The prepared HEK293T cell lysate was incubated with a dilution series of F4, F12, and F22 (0, 0.1, 1, 10, and 50 μM) for 2 h at 37 °C, followed by incubation with 1 μM final concentration of **JYQ-92** for 30 min at 37 °C. Cell lysate labeling was visualized as described above.

## Supporting Information Available

Supporting Information tables and figures, IC_50_ data, synthesis schemes of compound **STK793590** and the DiFMUAc assay reagent, and NMR and LC–MS spectral data (PDF)
High-throughput screening data (XLSX)
Molecular formula strings for all compounds (CSV)

## Accession Codes

Coordinates and structure factors have been deposited with the Protein Data Bank under accession numbers 7PA2 (PARK7–**8RK64**) and 7PA3 (PARK7–**JYQ-88**).

## Dedication

In memory of Huib Ovaa, who passed away too young on May 19th, 2020.

## Abbreviations

PARK7: Parkinson Disease Protein 7
DiFMUAc: 6,8-difluoro-4-methylumbelliferyl acetate
UCHL1: ubiquitin C-terminal hydrolase L1
SPR: surface plasmon resonance
FP: fluorescence polarization
SDS-PAGE: sodium dodecyl sulfate–polyacrylamide gel electrophoresis
HTS: high-throughput screening
HCTU: *O*-(1*H*-6-chlorobenzotriazole-1-yl)-1,1,3,3-tetramethyluronium hexafluorophosphate
DBU: 1,8-diazabicyclo[5.4.0]undec-7-ene
DiPEA: *N*,*N*-diisopropylethylamine
TFA: trifluoroacetic acid
