# Supplementary material for: Chemical Toolkit for PARK7: Potent, Selective, and High-Throughput
Source: J Med Chem. 2022 Sep 23;65(19):13288–304. doi: 10.1021/acs.jmedchem.2c01113 (PMC9574860; doi:10.1021/acs.jmedchem.2c01113)
Supplement: Supplementary file 1 — jm2c01113_si_001.pdf [file jm2c01113_si_001.pdf]

SUPPORTING INFORMATION

Chemical Toolkit for PARK7: Potent, Selective and High-Throughput

Yuqing Jia,[a] Robbert Q. Kim,[a] Raymond Kooij,[a] Huib Ovaa,[a] Aysegul Sapmaz,*[a] and Paul P. Geurink*[a]

[a] Oncode Institute & Department of Cell and Chemical Biology, Leiden University Medical Center, Einthovenweg 20, 2333 ZC, Leiden, The Netherlands

* Corresponding authors: p.p.geurink@lumc.nl, a.sapmaz@lumc.nl

Contents

Table S1. Data processing and refinement statistics for the PARK7-inhibitor cocrystal structures. 3

Table S2. Structures overview of fragments for hit validation. 4

Figure S1. Electron density maps of (A) PARK7-**8RK64** (PDB: 7PA2) and (B) PARK7-**JYQ-88** (PDB: 7PA3) co-crystal structures....8

Figure S2. Inhibitory activity of **8RK64** towards PARK7....9

Figure S3. IC_{50} determination of **STK793590** for PARK7 by DiFMUAc assay....10

Figure S4. IC_{50} determination of **JYQ-92**, **JYQ-93**, and **JYQ-107** for UCHL1 using Ub-AMC as the substrate. 11

Figure S5. Fluorescence labeling of DUBs to investigate their remaining activity by Rh-Ub-PA after the treatment with PARK7 inhibitor **JYQ-88** and probe **JYQ-92**. 12

Figure S6. The development of the FP assay. 13

Figure S7. Change in FP value over time at different concentrations of inhibitor **JYQ-55**.14

Figure S8. Heatmap displaying the validation of hits using the DiFMUAc assay....15

Figure S9. Uncropped and unprocessed gel images in Figure 4. 16

Figure S10. Uncropped and unprocessed gel images in Figure 6E....18

Synthesis of **STK793590** 19

Synthesis of **DiFMUAc** assay reagent....19

NMR spectra 20

LC-MS of synthesized compounds 35

Table S1. Data processing and refinement statistics for the PARK7-inhibitor cocrystal structures.

	**PARK7 +8RK64**	**PARK7 +JYQ-88**
Crystallization conditions	0.1 M Bicine pH 9.0<br>10% PEG 20000<br>2% w/v 1,4-Dioxane	0.1 M Ammonium sulfate<br>0.1 M Bis-Tris pH 5.5<br>25% PEG 3350
PDB accession code	*7PA2*	*7PA3*
Space group	P 65 2 2	P 65 2 2
Cell dimensions		
a (Å)	66.86	66.74
b (Å)	66.86	66.74
c (Å)	179.69	176.78
α (°)	90	90
β (°)	90	90
γ (°)	120	120
Processing statistics		
Resolution (Å)<br>Outer shell (Å)	59.90-1.21<br>1.23-1.21	58.93-1.42<br>1.44-1.42
Beamline	DLS i24	DLS i04
Wavelength (Å)	0.96871	0.97996
Observed reflections	1324702 (34068)	1462173 (19490)
Unique reflections	73359 (3558)	44987 (2131)
R_{pim}	0.028 (0.969)	0.033 (1.416)
CC(1/2)	0.999 (0.447)	1 (0.335)
Multiplicity	18.1 (9.6)	32.5 (9.1)
Completeness	100 (100)	100 (98.9)
Mean (I/σ(I))	16.1 (1.2)	17.5 (0.8)
Refinement statistics		
Monomers in ASU	1	1
No of protein atoms	1384	1380
R_{work}	0.132	0.157
R_{free}	0.155	0.194
RMSD from ideality		
Bond lengths (Å)	0.0171	0.0148
Bond angles (°)	1.9326	1.8544
Chiral volume (Å^3)	0.1216	0.1094
Ramachandran plot		
Favoured (%)	98.9	98.9
Disallowed (%)	0.5	0
Average B-values (Å^2)	21.0	30
	Values within parentheses are for the outer resolution shell	

Table S2. Structures overview of fragments for hit validation.

Compound	Enamine catalog ID	Structure
F1	Z1954803958	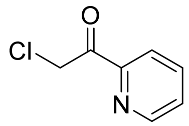
F2	Z802671642	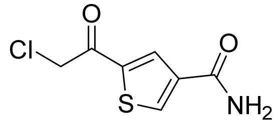
F3	Z3236282166	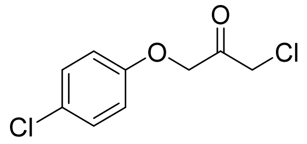
F4	Z763366298	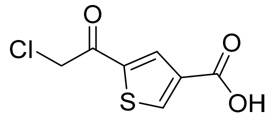
F5	Z2491497305	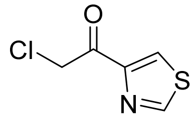
F6	Z2768504793	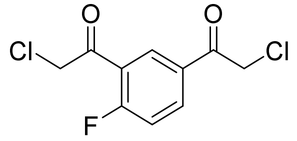
F7	Z2492395560	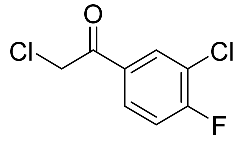
F8	Z2491498268	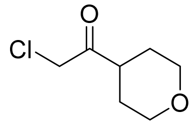
F9	Z235333089	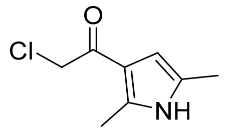
F10	Z85923410	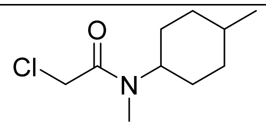

F11	Z56922153	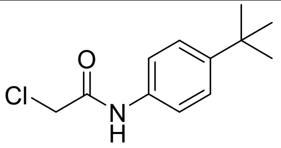
F12	Z56886389	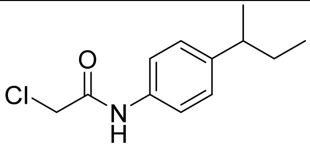
F13	Z86023354	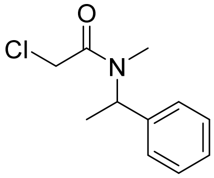
F14	Z56896308	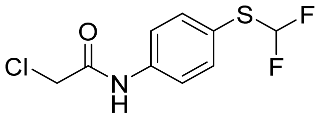
F15	Z190662892	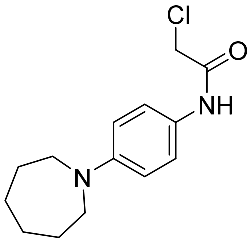
F16	Z220564178	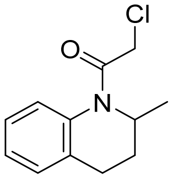
F17	Z56886400	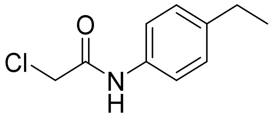
F18	Z56896170	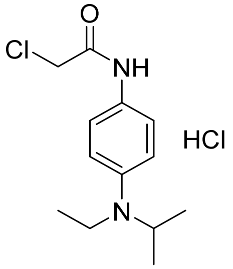
F19	Z57051055	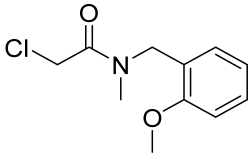

F20	Z1562123076	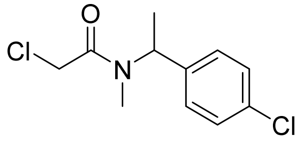
F21	Z1672273218	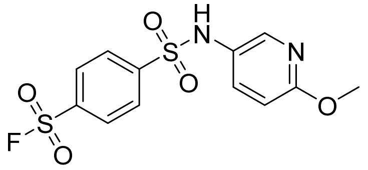
F22	Z1764361494	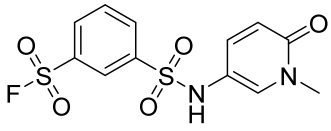
F23	Z3396482545	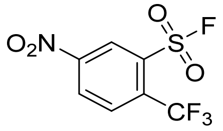
F24	Z2509451091	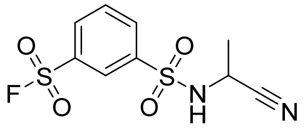
F25	Z1832812427	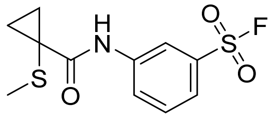
F26	Z1741956298	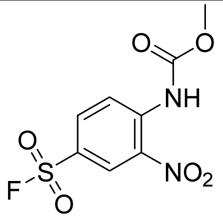
F27	Z1764398070	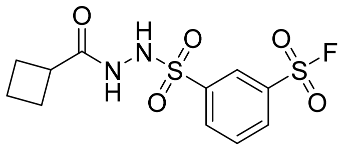
F28	Z1713569375	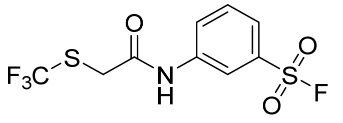
F29	Z1713569513	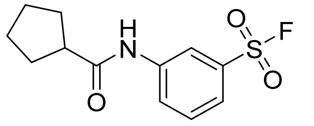

F30	Z1713568698	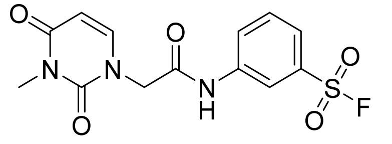
F31	Z1764375816	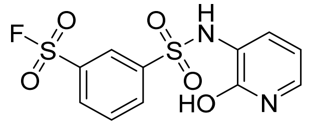
F32	Z3242849759	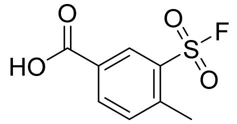
F33	Z3243697698	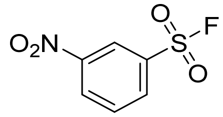
F34	Z3223579620	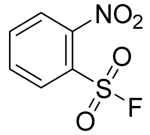
F35	Z2509462762	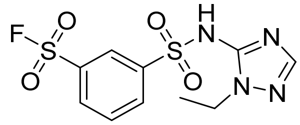
F36	Z2509450956	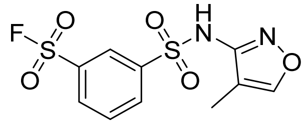
F37	Z3267389796	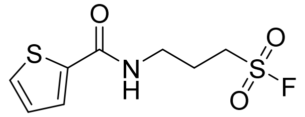
F38	Z2509455014	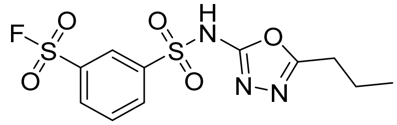
F39	Z1832812448	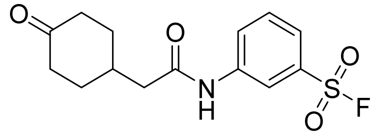
F40	Z1764309349	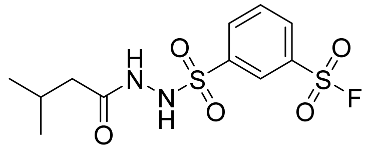

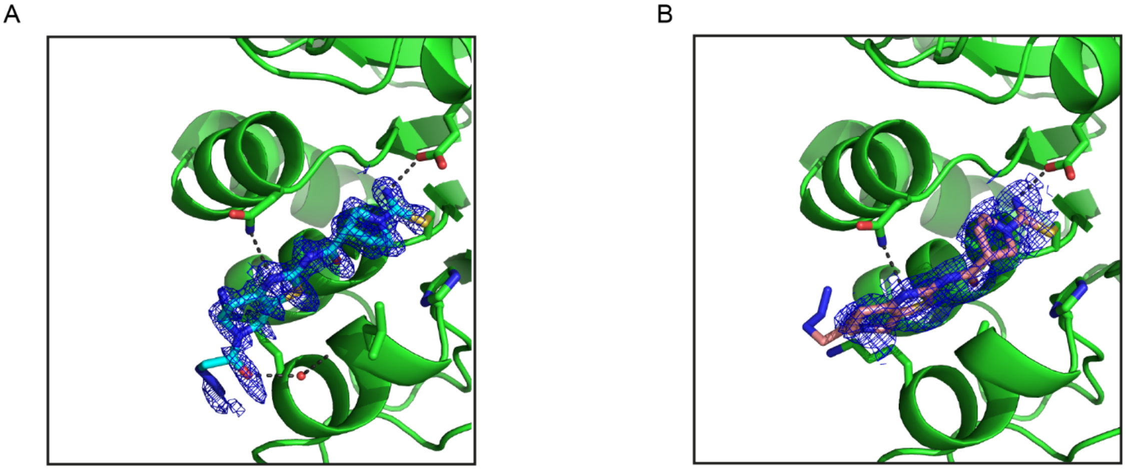

Figure S1. Electron density maps of (A) PARK7-**8RK64** (PDB: 7PA2) and (B) PARK7-**JYQ-88** (PDB: 7PA3) co-crystal structures.

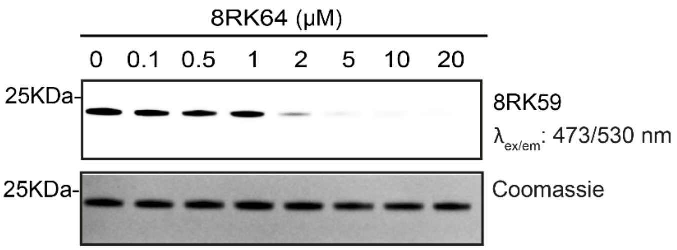

Figure S2. Inhibitory activity of **8RK64** towards PARK7. PARK7 (1 μM) was incubated with increasing concentrations of **8RK64**, followed by incubation with **8RK59** for 30 min. at 37 °C.

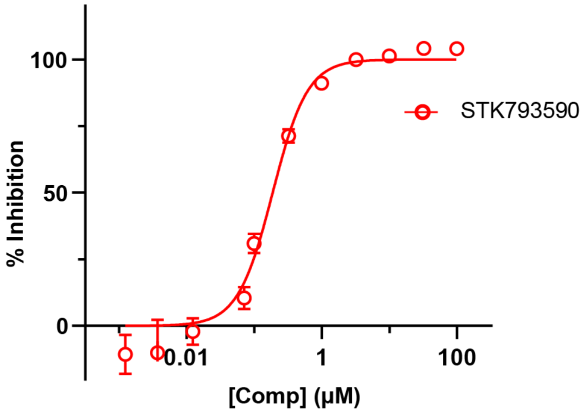

Figure S3. IC_{50} determination of **STK793590** for PARK7 by DiFMUAc assay.

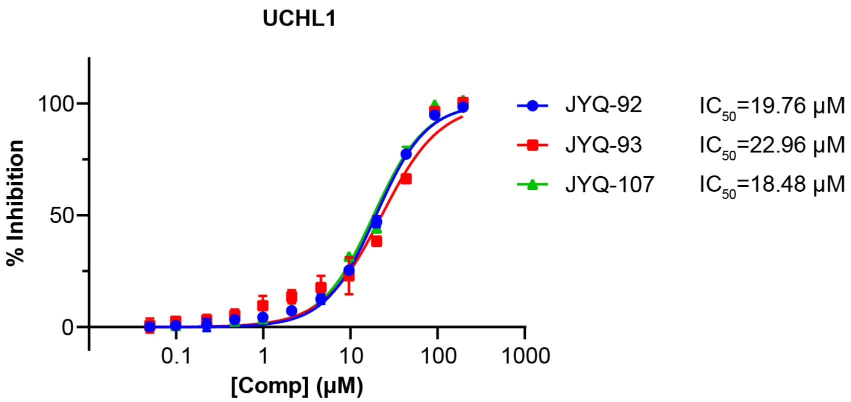

Figure S4. IC_{50} determination of **JYQ-92**, **JYQ-93**, and **JYQ-107** for UCHL1 using Ub-AMC as the substrate.

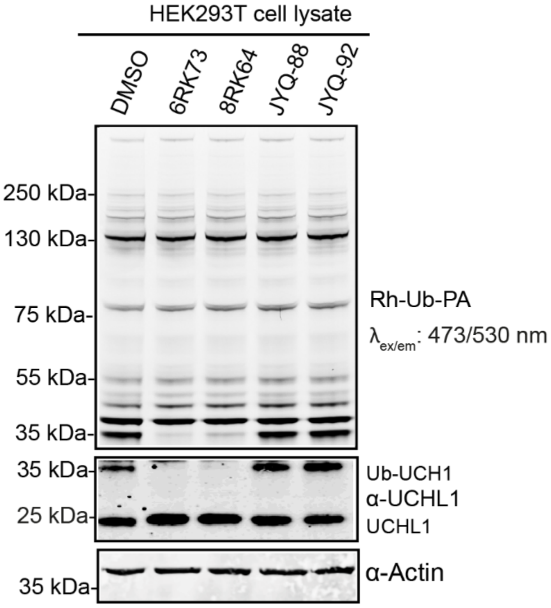

Figure S5. Fluorescence labeling of DUBs to investigate their remaining activity by Rh-Ub-PA after the treatment with PARK7 inhibitor **JYQ-88** and probe **JYQ-92**. UCHL1 inhibitors, **6RK73** and **8RK64**, were used as a control. HEK293T cell lysate was treated with 5 μM final concentration of the indicated compounds for 1 h at 37 °C, followed by incubating with Rho-Ub-PA for 30 min at 37 °C. The reactions were stopped by the addition of NuPAGE™ LDS sample buffer (4X). Samples were resolved by SDS-PAGE using a 4-12% Bis-Tris gel with MOPS SDS running buffer (Novex, NuPAGE) and visualized by fluorescence scanning on a Typhoon FLA 9500 (GE Healthcare Life Sciences) using a Rhodamine channel (λex/em 473/530 nm), followed by transferring to Nitrocellulose membranes and Western blot analysis.

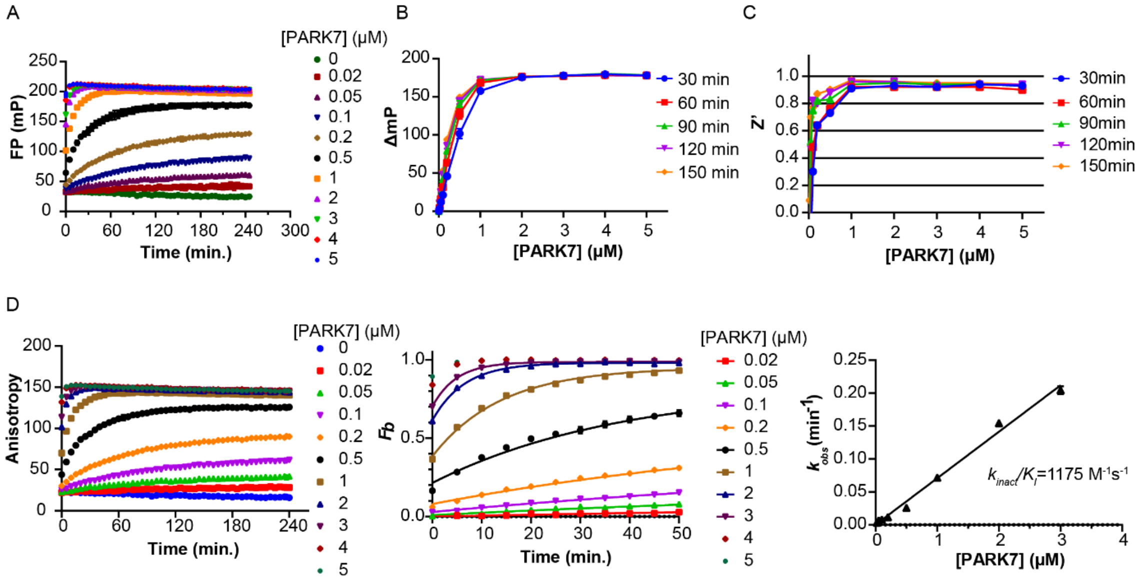

Figure S6. The development of the FP assay.(A) Relation between FP value and incubation times at different concentrations of PARK7. (B) Relation between the change in FP value and PARK7 concentration at different incubation times. (C) The corresponding Z' value. Panel B and C are full graphs of the graphs in Figure 5B in the main paper. (D) Determination of k_{inact}/K_I value for FP probe **JYQ-107**. Left panel: Relation between anisotropy and incubation times at different concentrations of PARK7. Middle panel: The probe bound fraction (F_b) was calculated for each datapoint by normalization of the anisotropy data to fully unbound (no PARK7) and fully bound (plateau) states followed by baseline correction. The pseudo-first order rate constant k_{obs} for each PARK7 concentration was determined by fitting the F_b data to the pseudo-first order association equation Y=Y0 + (Plateau-Y0)*(1-exp(-k_{obs}*t)), where Y=F_b. The data for the highest concentrations of PARK7 (4 and 5 µM) could not be fitted because the reaction was too fast. Right panel: k_{obs} values were plotted against PARK7 concentration and the k_{inact}/K_I value was calculated from the slope of a linear fit of the data.

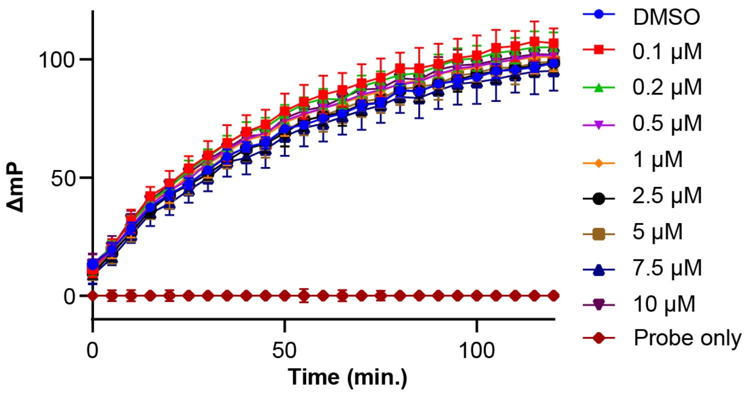

Figure S7. Change in FP value over time at different concentrations of inhibitor **JYQ-55**. PARK7 was incubated with increasing concentrations of inactive compound **JYQ-55** for 60 min. followed by the addition of FP probe **JYQ-107** and the change in FP was monitored over time.

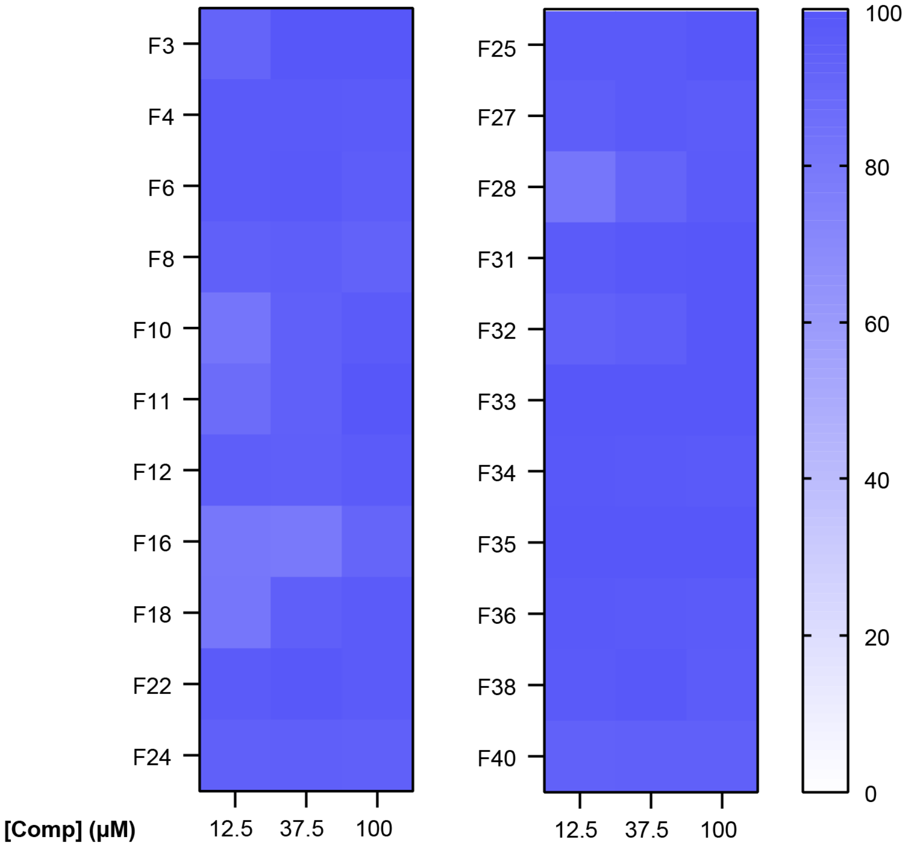

Figure S8. Heatmap displaying the validation of hits using the DiFMUAc assay. (White: 0% inhibition, blue: 100% inhibition.)

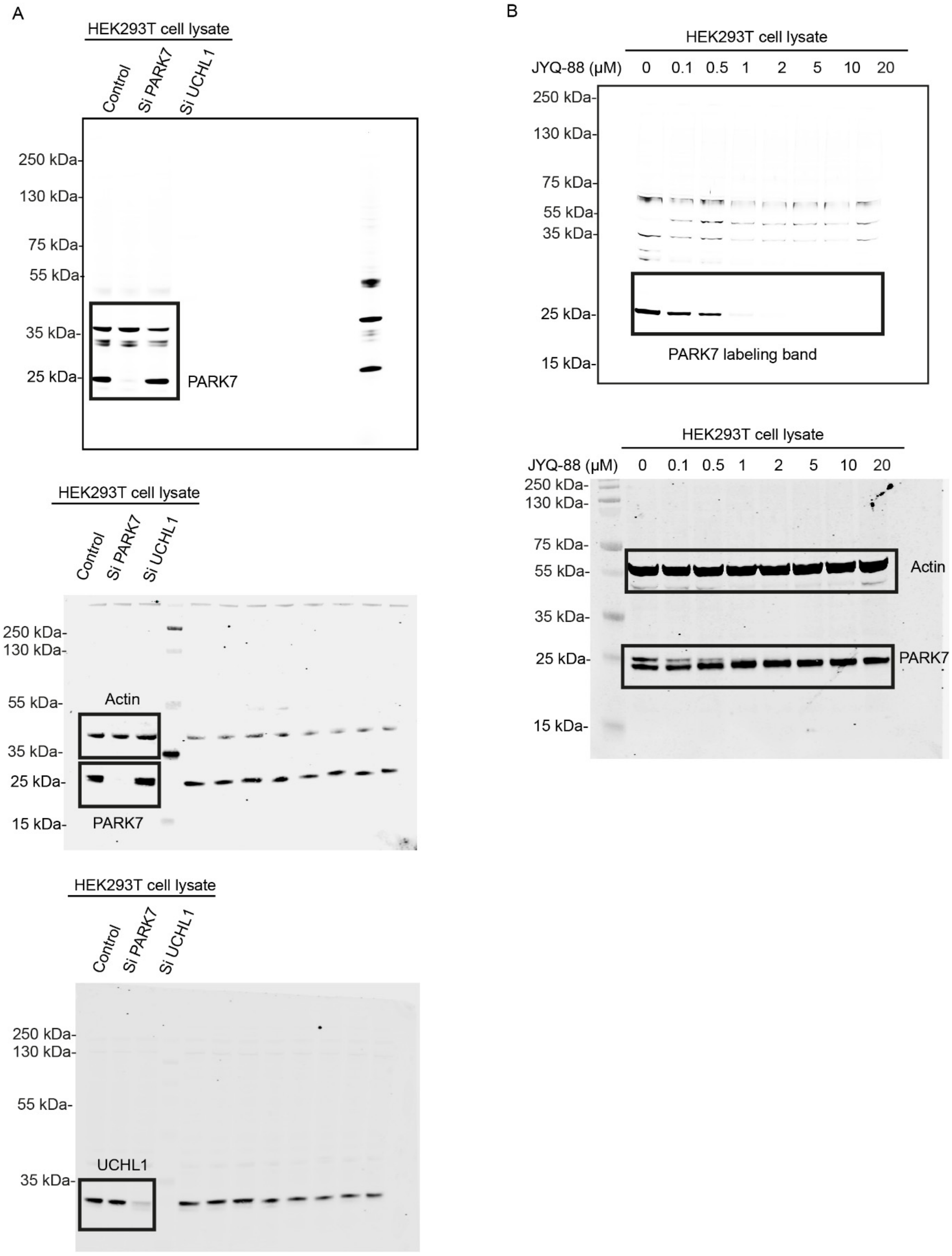

Figure S9. Uncropped and unprocessed gel images in Figure 4. (A) Related to Figure 4D: In-gel fluorescence scanning of PARK7 activity by **JYQ-92** in HEK293T cell lysate with/without depletion of PARK7 or UCHL1 (top panel) and immunoblot of PARK7 with actin as a loading control (middle panel), together with immunoblot of UCHL1 (bottom panel). (B) Related to

Figure 4E: In-gel fluorescence scanning of PARK7 activity by JYQ-92 in HEK293T cell lysate after treatment with inhibitor **JYQ-88** (top panel) and immunoblot of PARK7 with actin as a loading control (bottom panel).

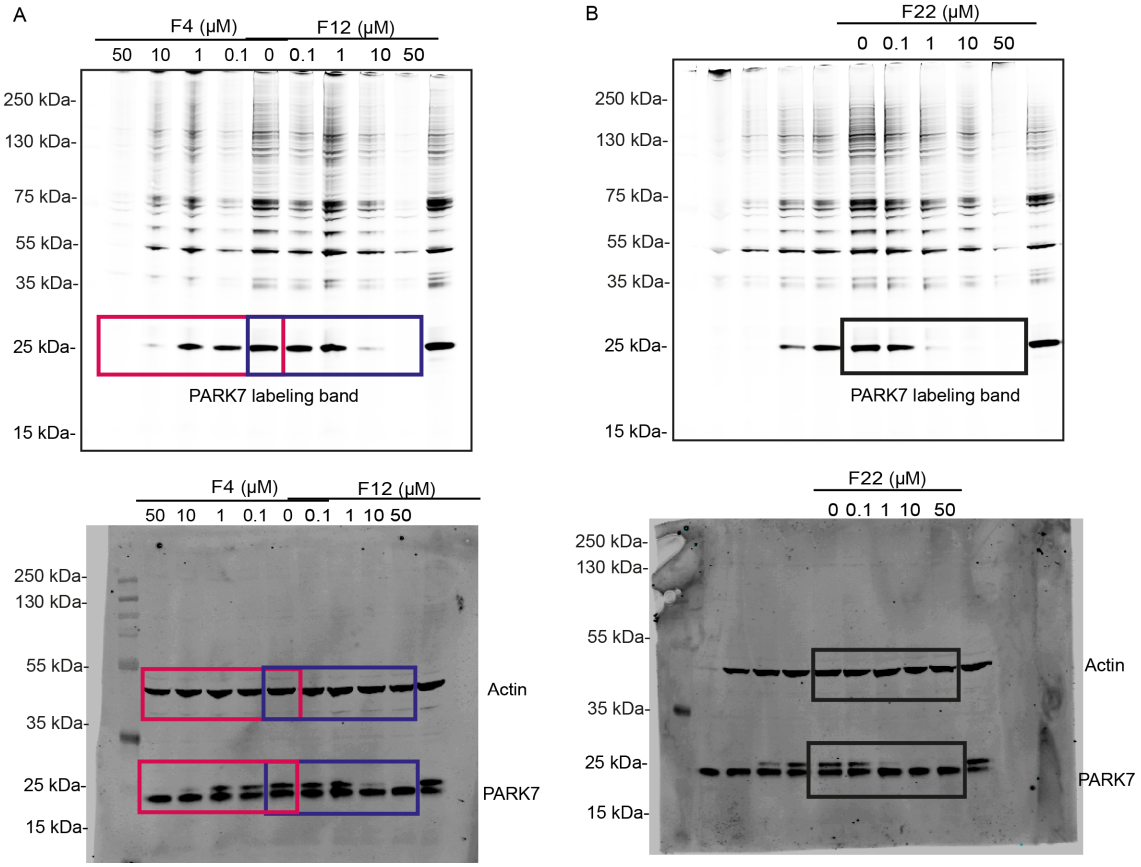

Figure S10. Uncropped and unprocessed gel images in Figure 6E. (A) In-gel fluorescence scanning of PARK7 activity by **JYQ-92** in HEK293T cell lysate after treatment with fragment F4 (left from the top panel) and F12 (right from the top panel), and immunoblot of PARK7 with actin as a loading control (bottom panel). (B) In-gel fluorescence scanning of PARK7 activity in HEK293T cell lysate by **JYQ-92** after treatment with fragment F22 (top panel) and immunoblot of PARK7 with actin as a loading control (bottom panel).

Synthesis of STK793590

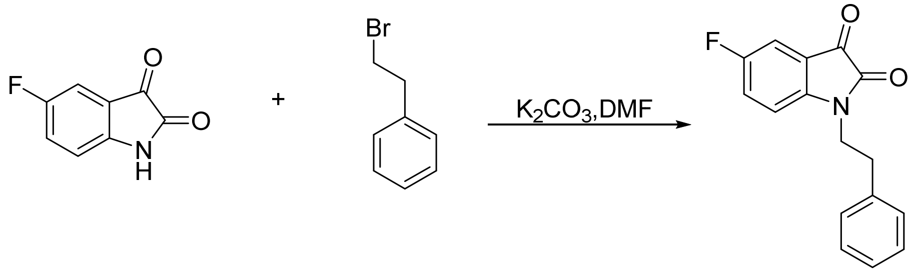

STK793590

Synthesis of DiFMUAc assay reagent

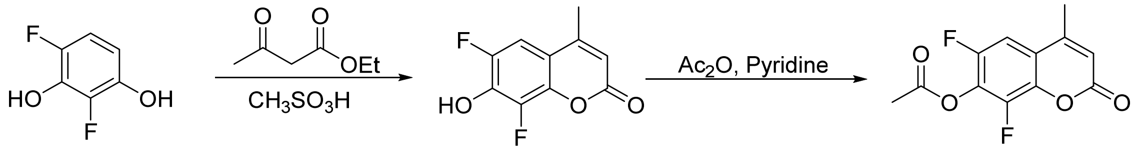

DiFMUAc

NMR spectra

^{1}H-NMR and ^{13}C-NMR of compound 2b in $CDCl_3$.

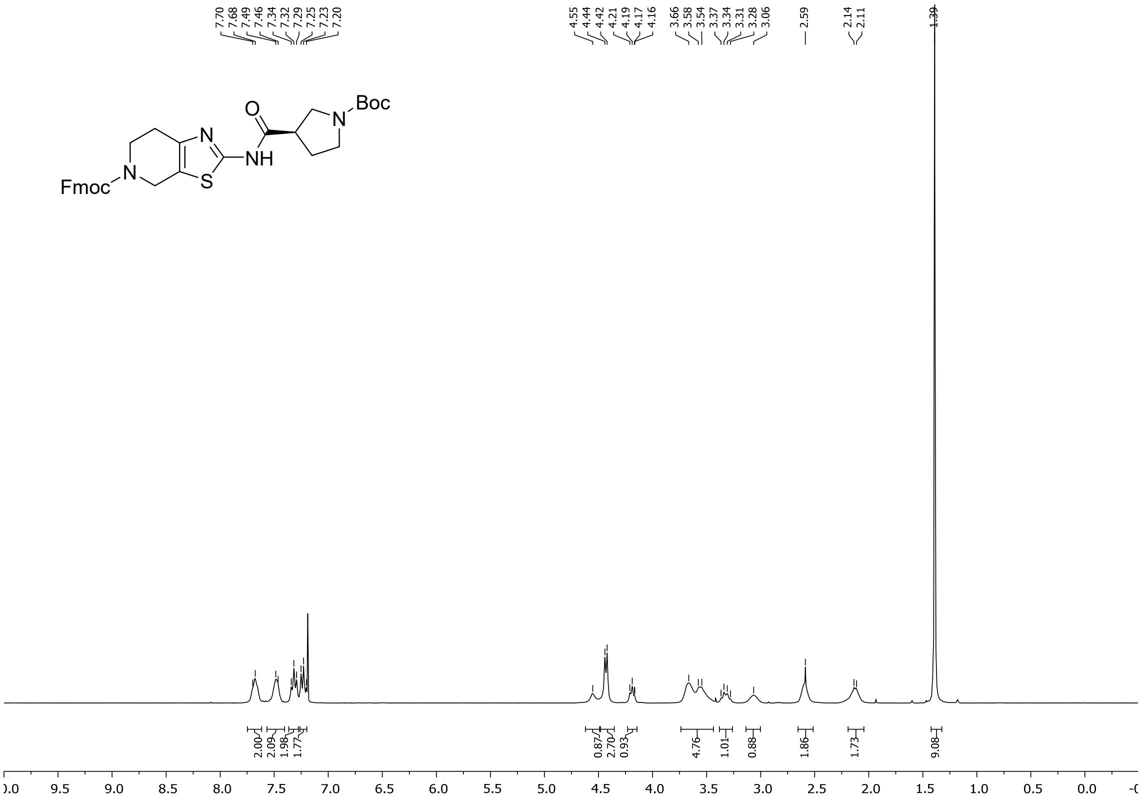

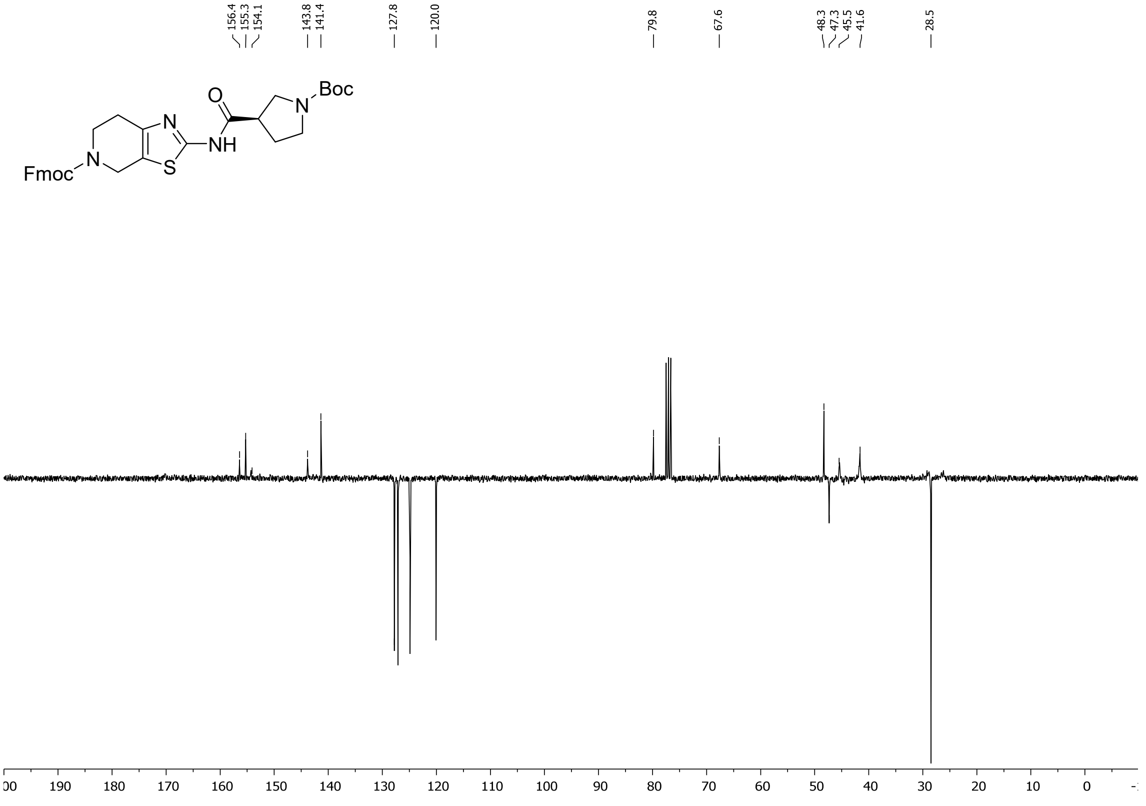

^{1}H-NMR and ^{13}C-NMR of compound 3a in $CDCl_3$.

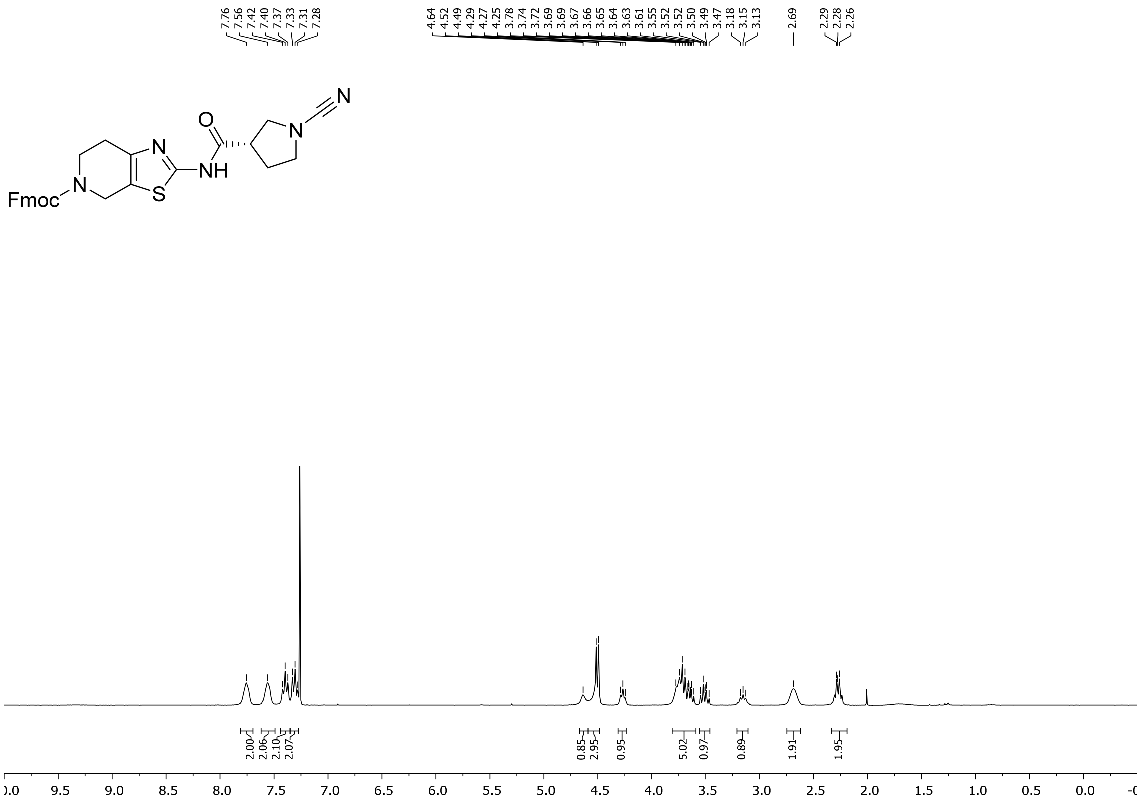

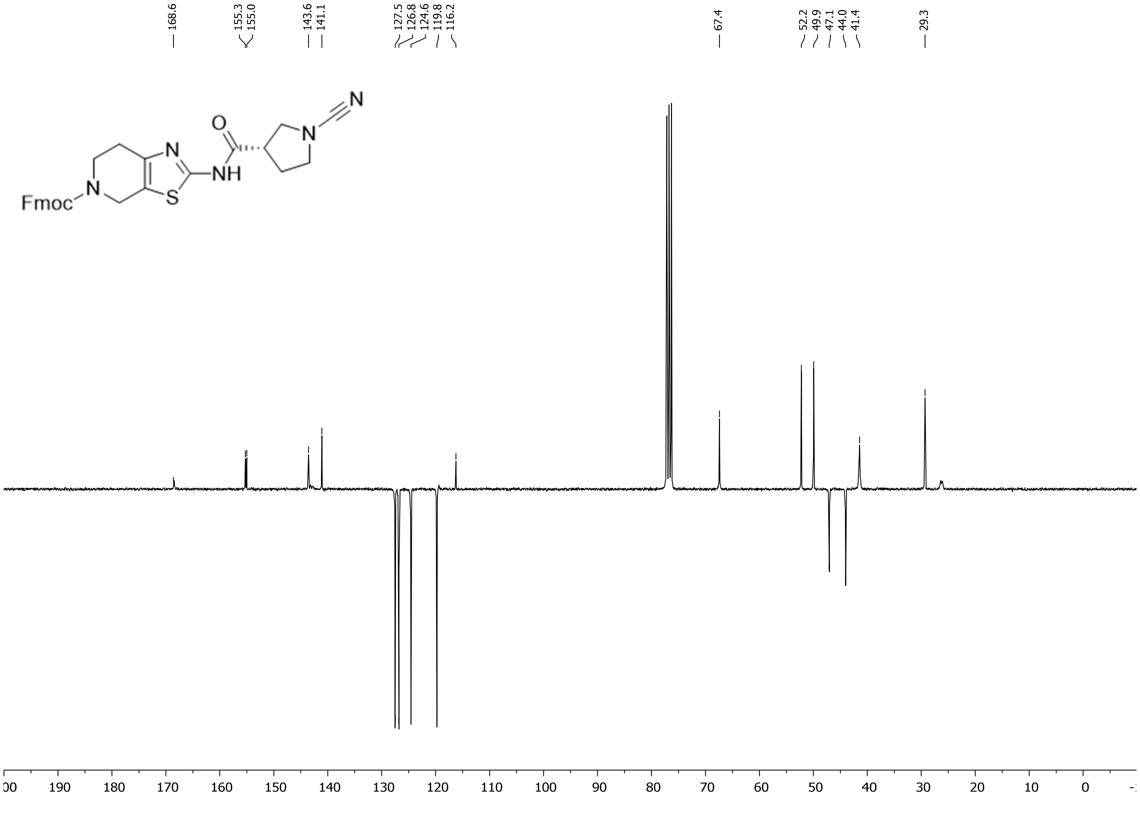

^{1}H-NMR and ^{13}C-NMR of compound 3b in $CDCl_3$.

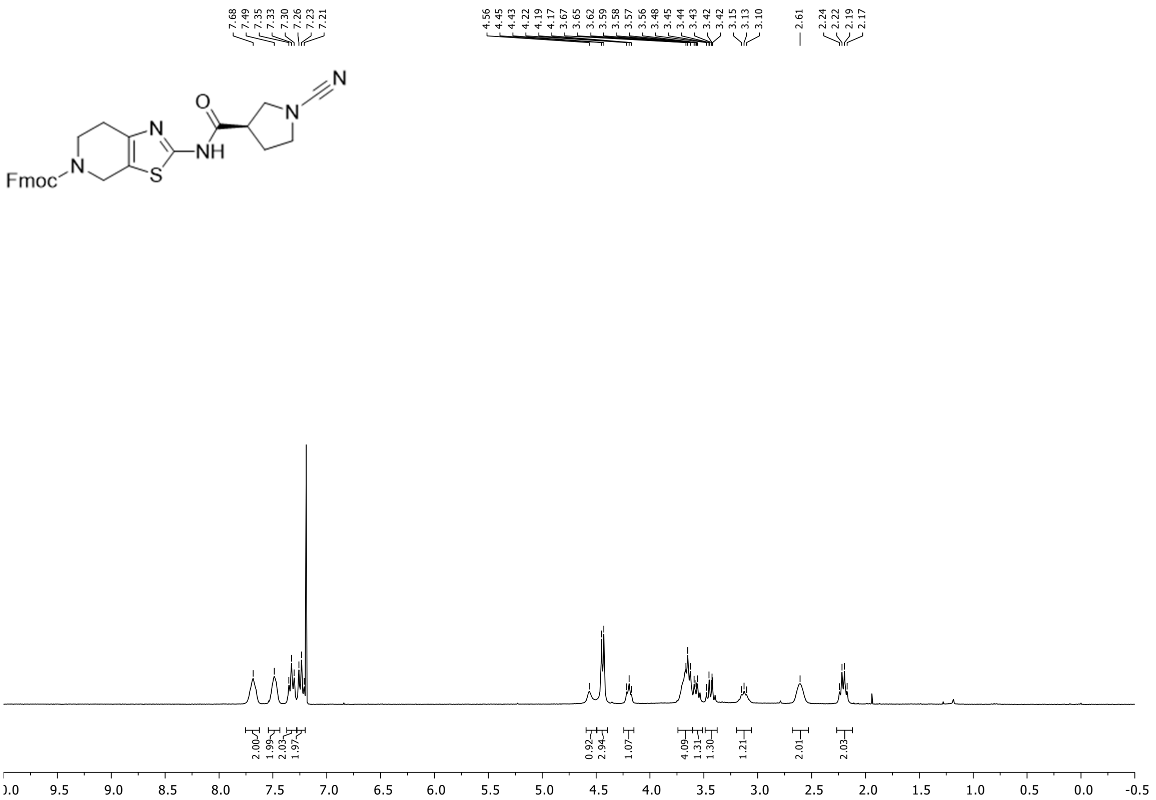

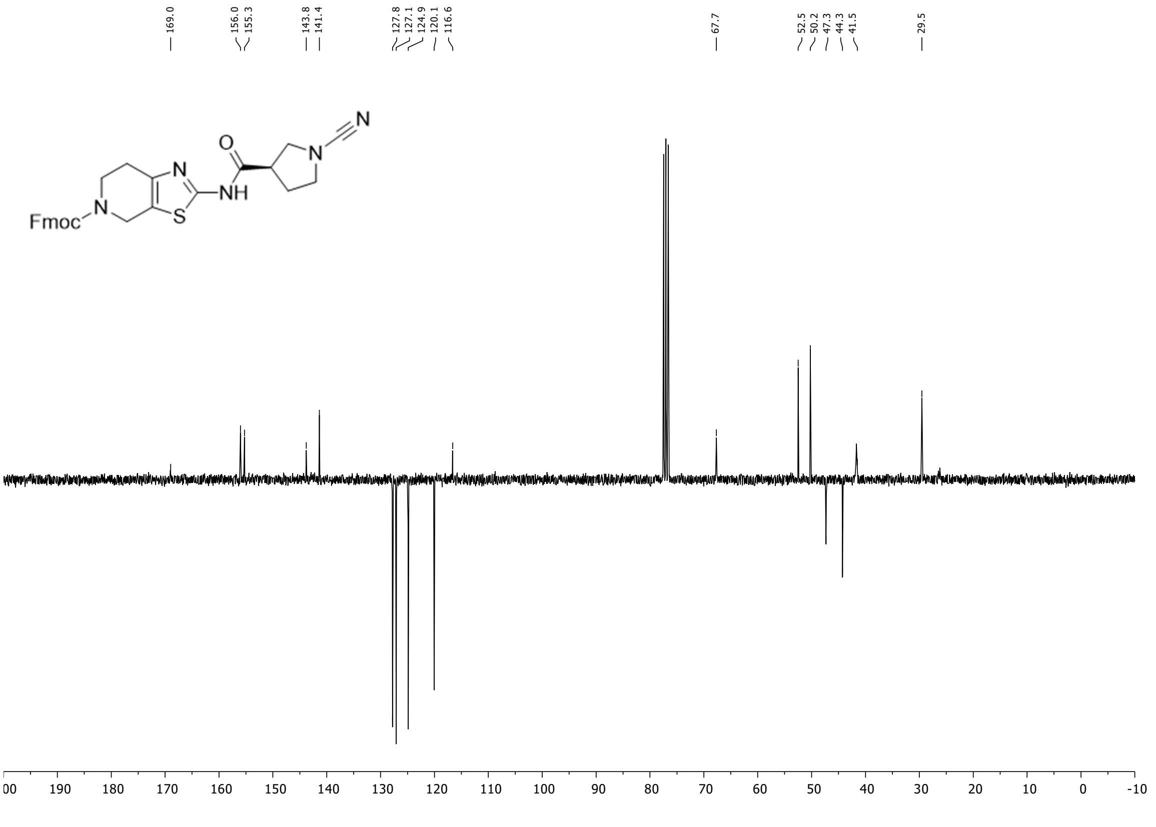

^{1}H-NMR and ^{13}C-NMR of compound JYQ-55 in DMSO.

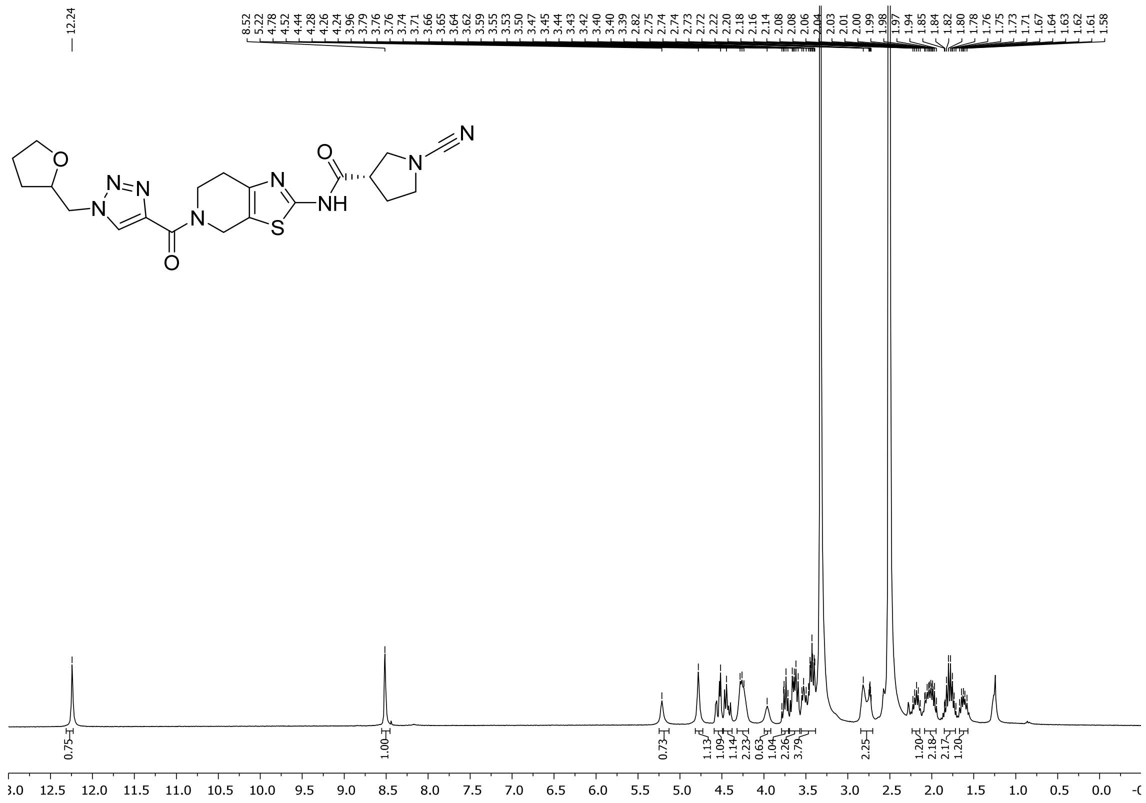

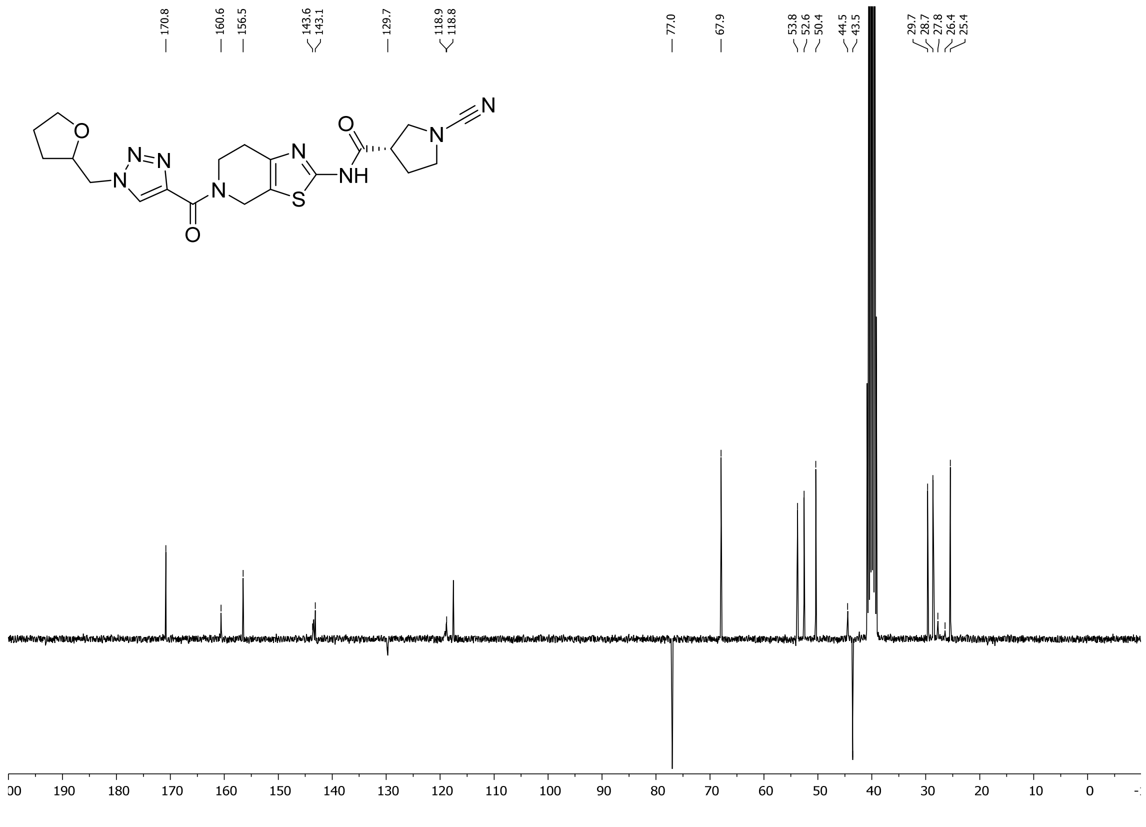

^{1}H-NMR and ^{13}C-NMR of compound JYQ-76 in DMSO.

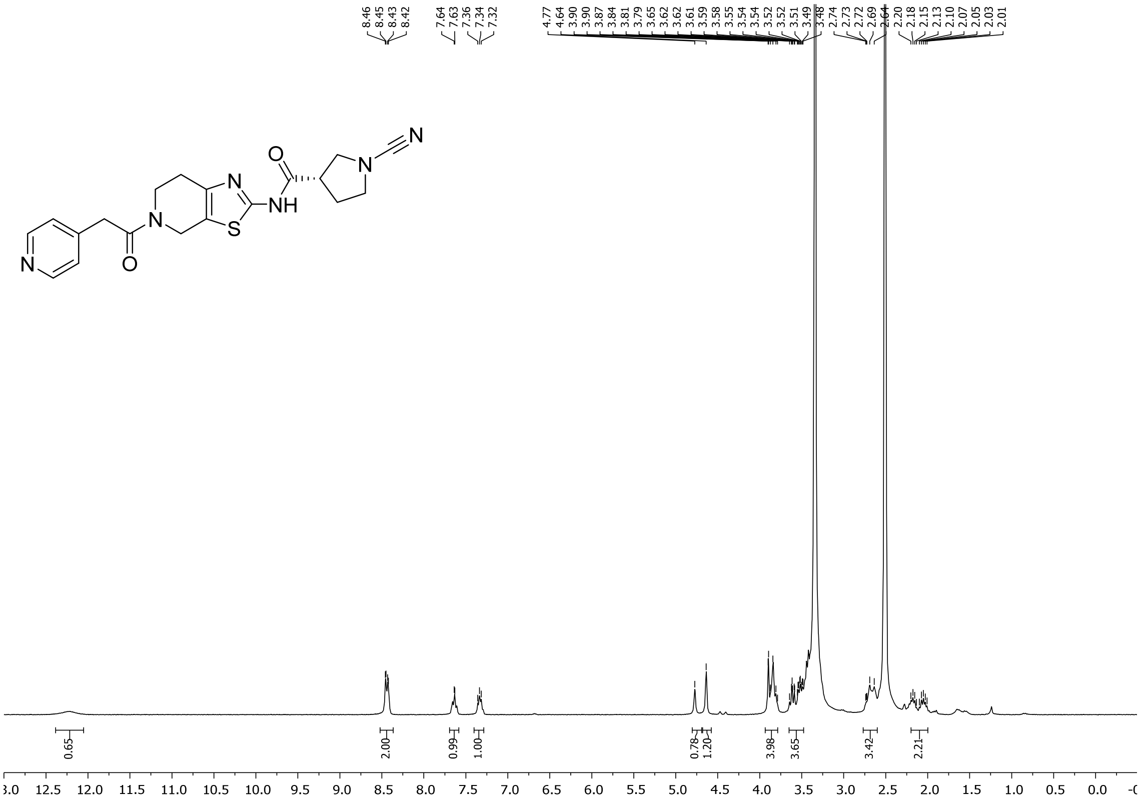

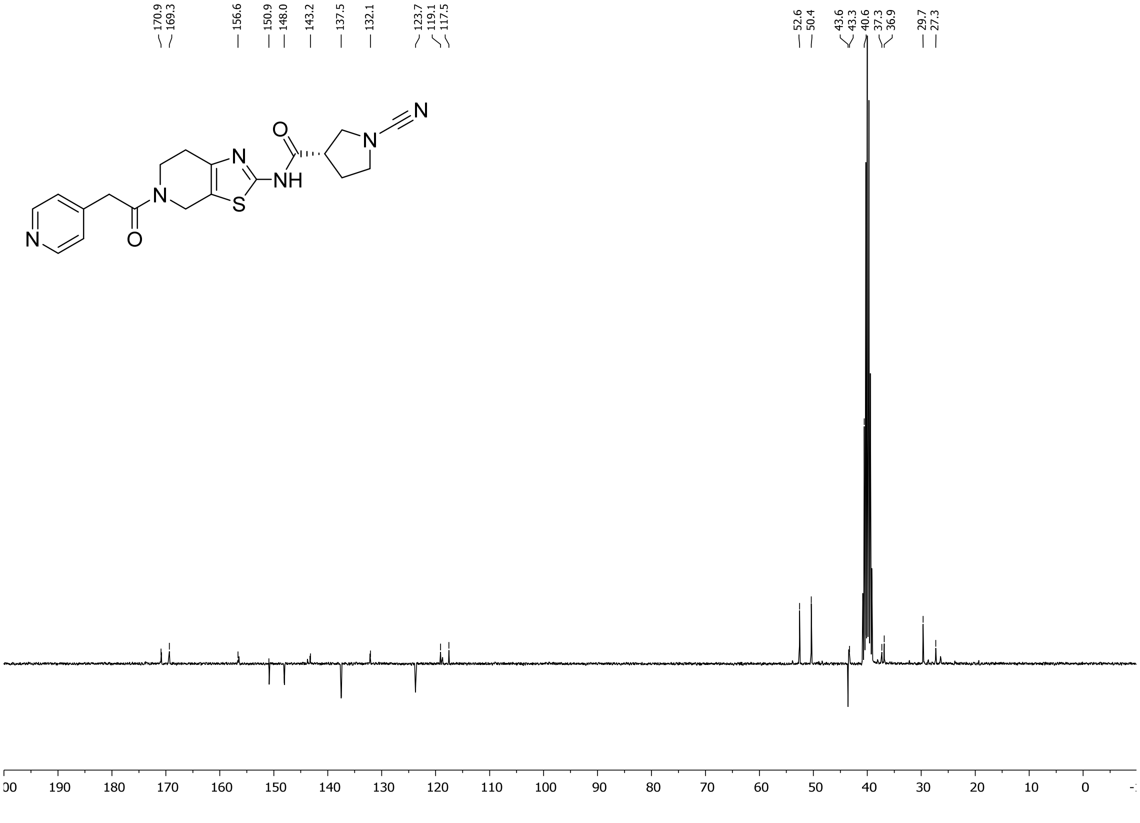

^{1}H-NMR and ^{13}C-NMR of compound JYQ-77 in DMSO.

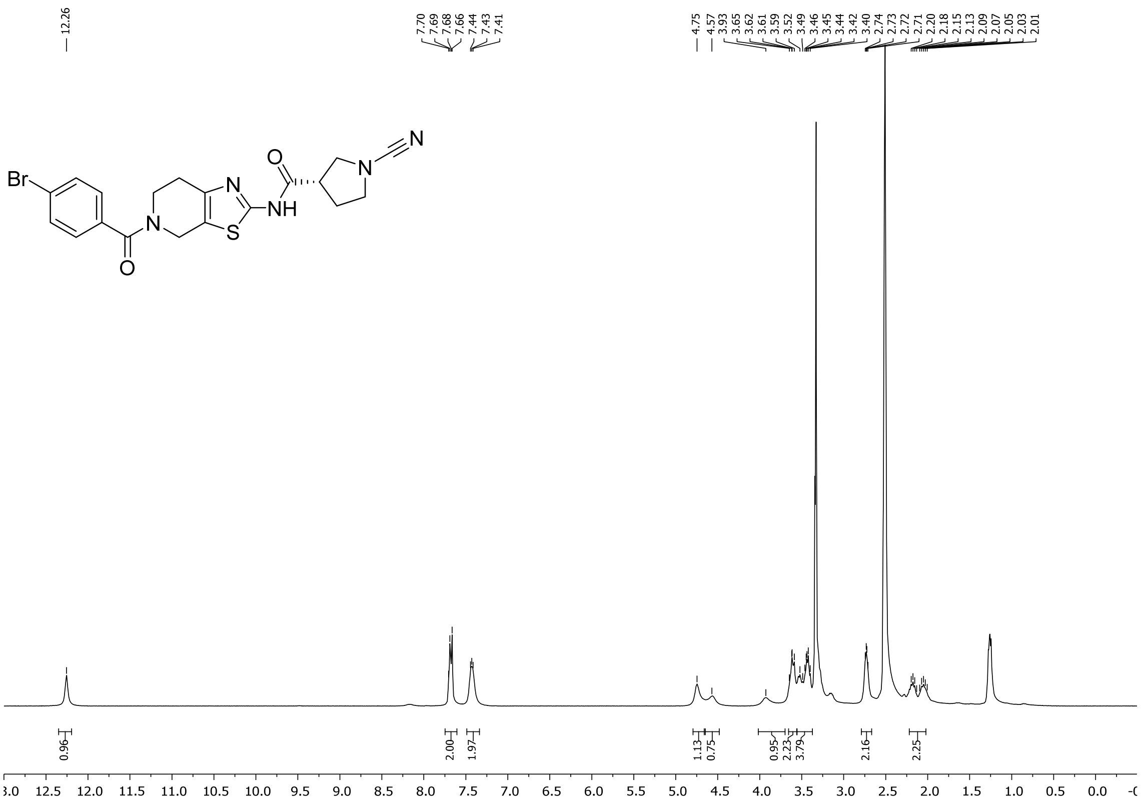

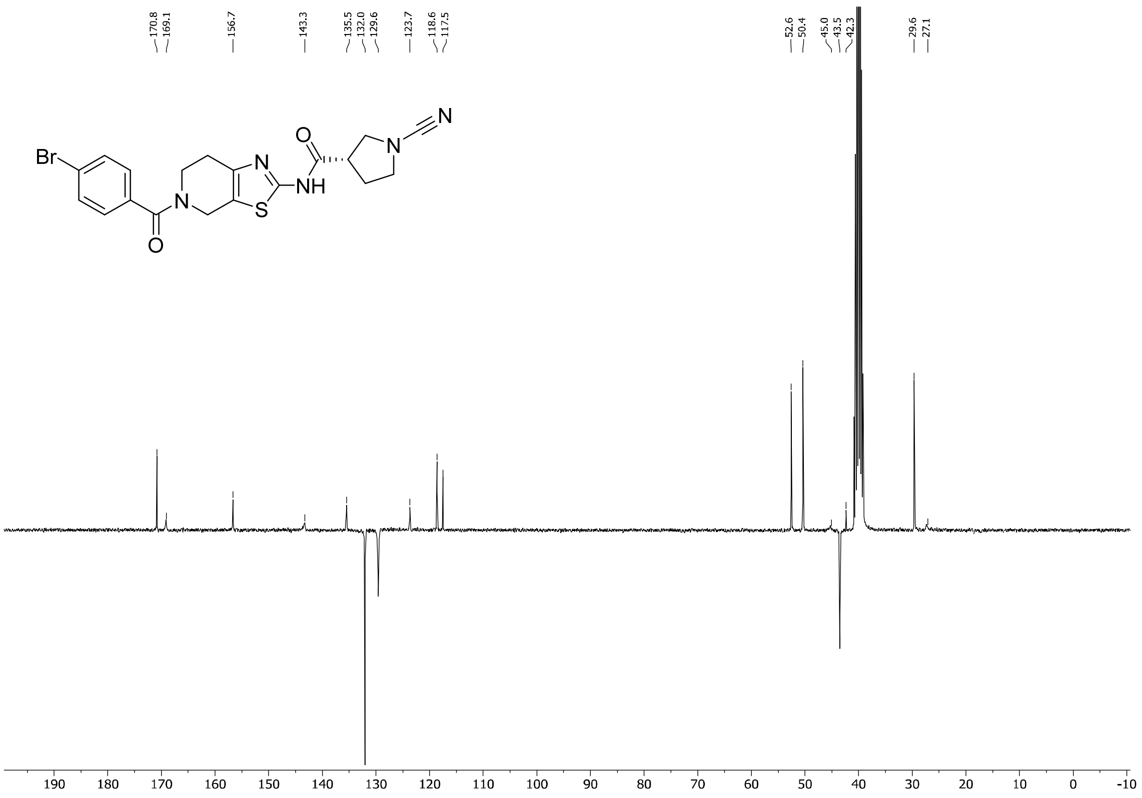

^{1}H-NMR and ^{13}C-NMR of compound JYQ-78 in DMSO.

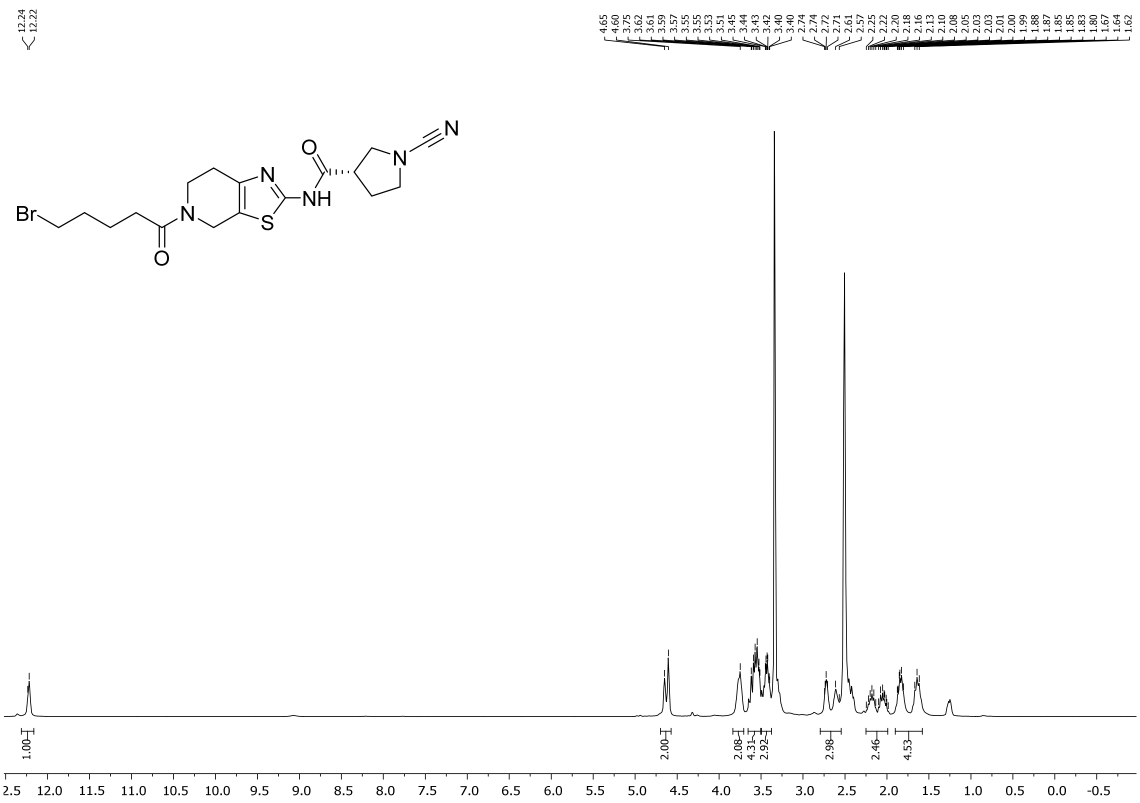

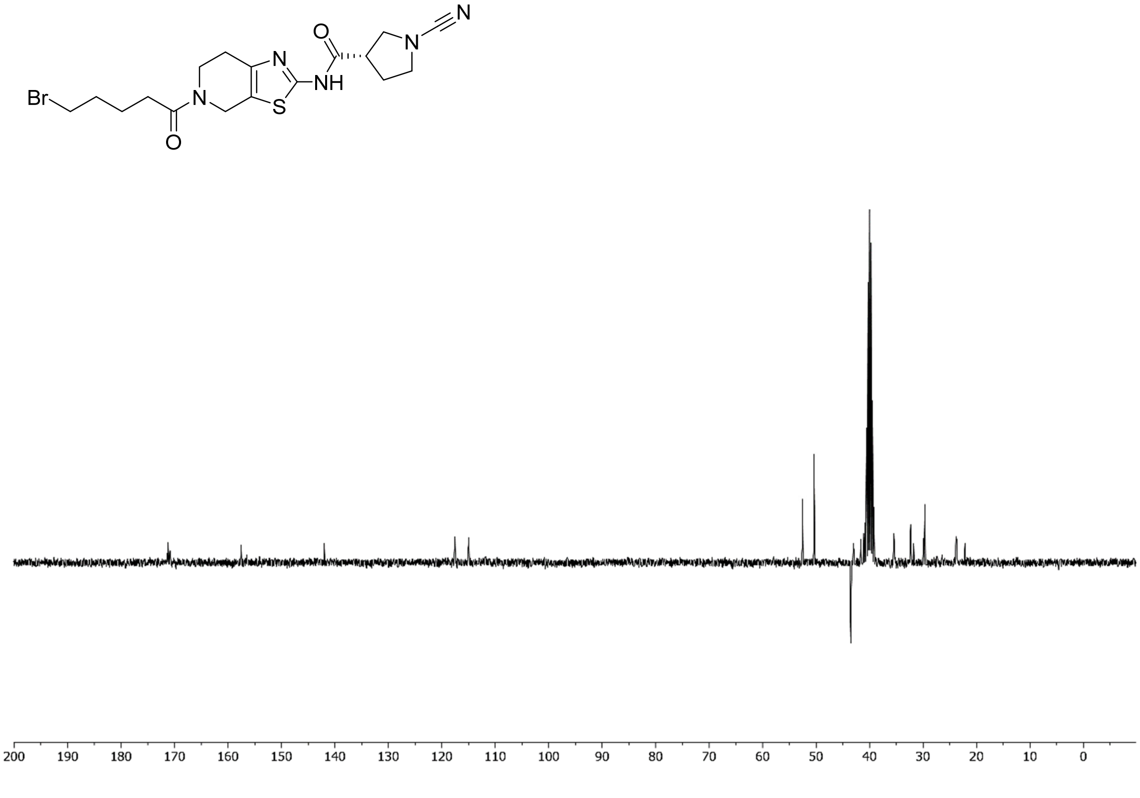

^{1}H-NMR and ^{13}C-NMR of compound JYQ-79 in DMSO.

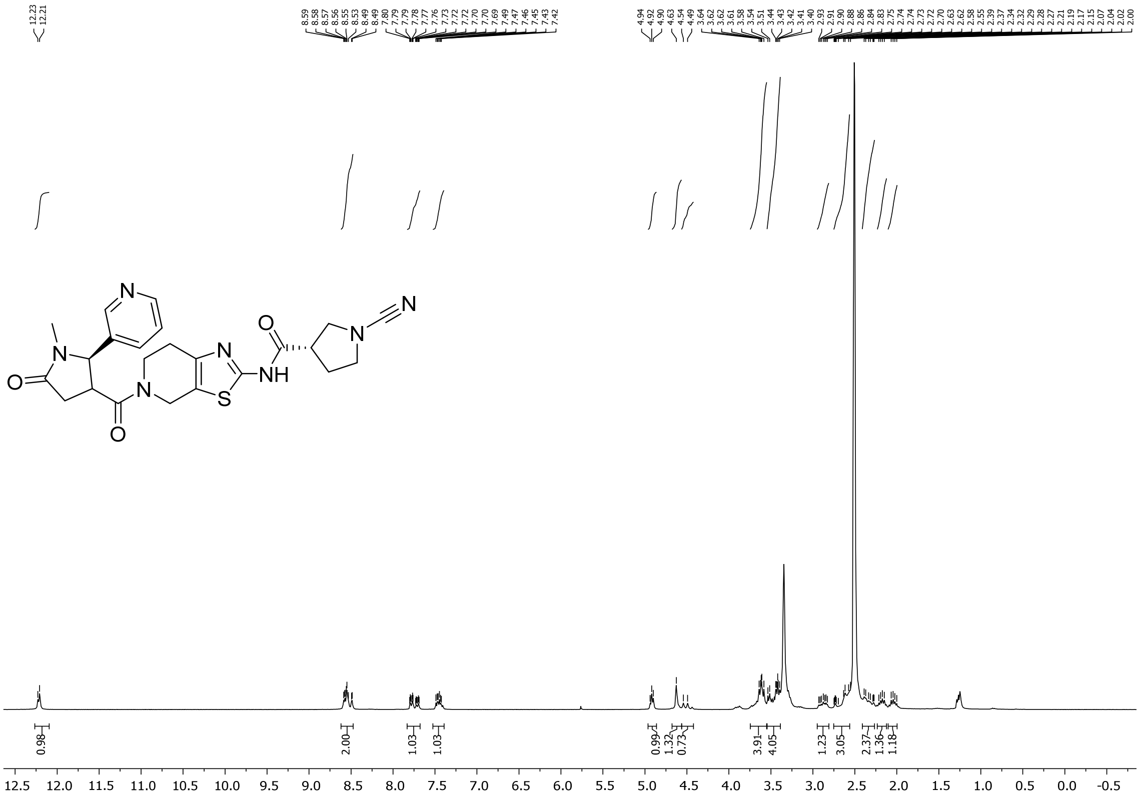

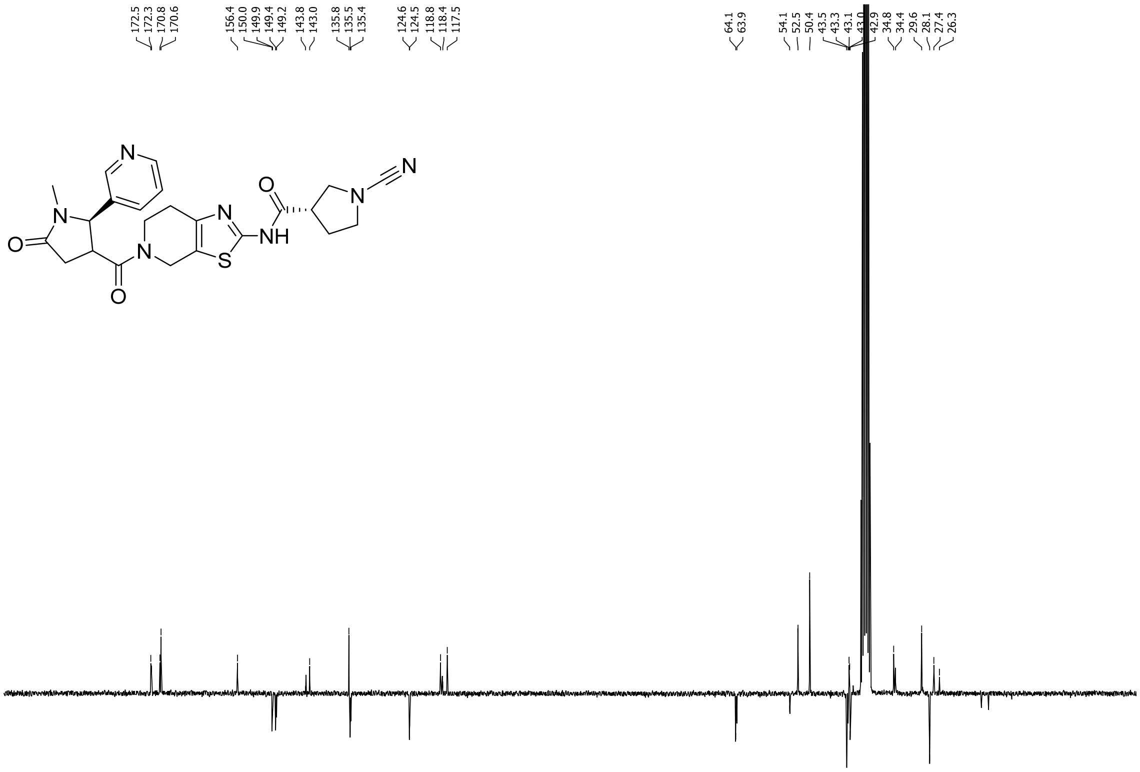

^{1}H-NMR and ^{13}C-NMR of compound JYQ-83 in DMSO.

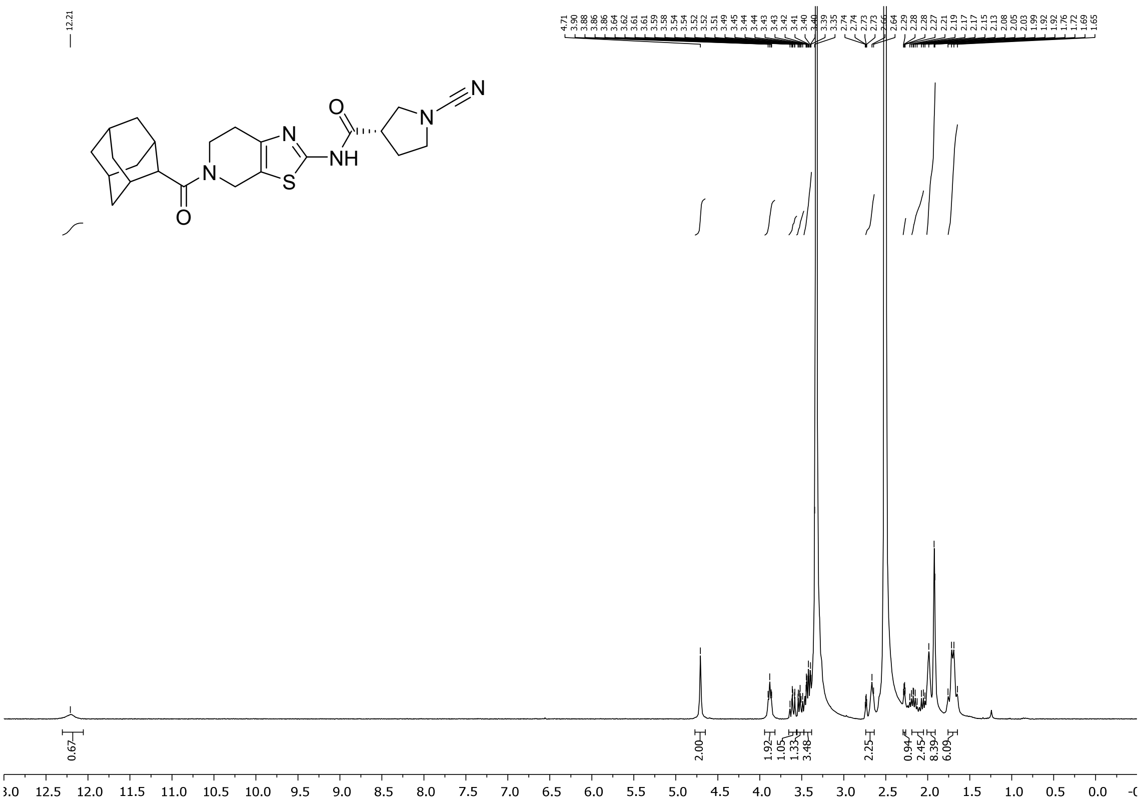

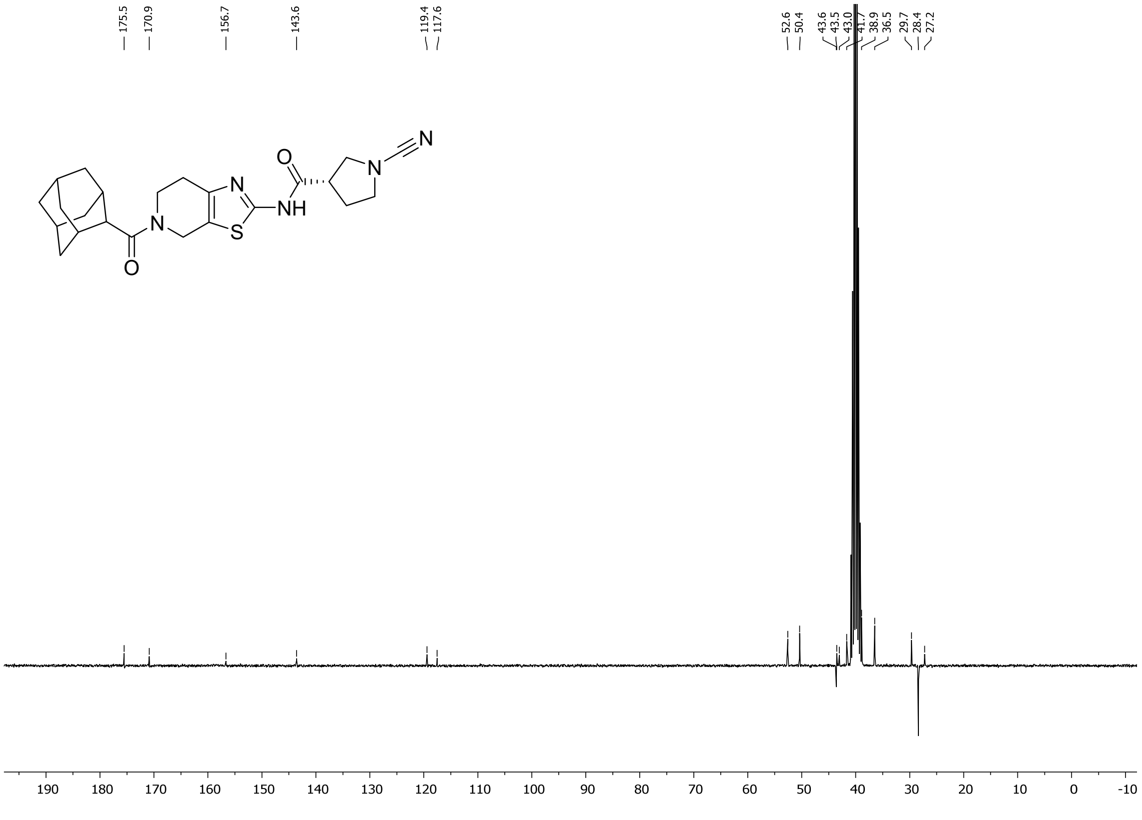

^{1}H-NMR and ^{13}C-NMR of compound JYQ-88 in $CDCl_3$.

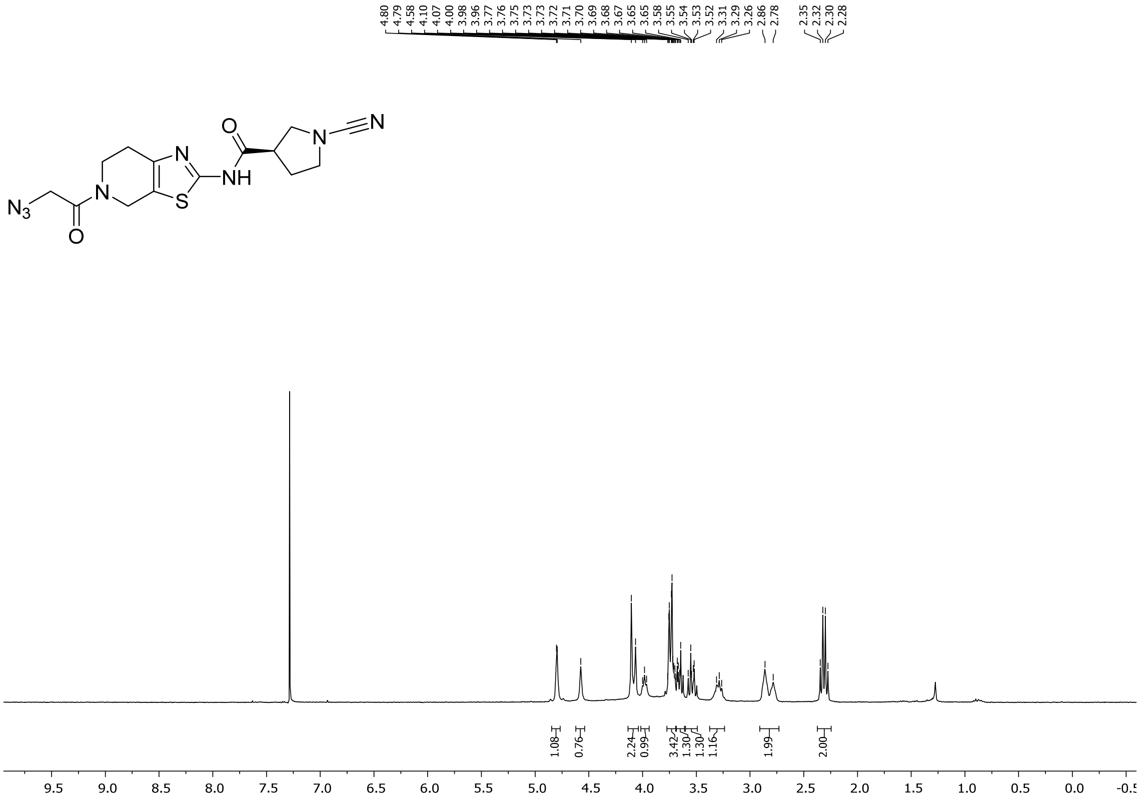

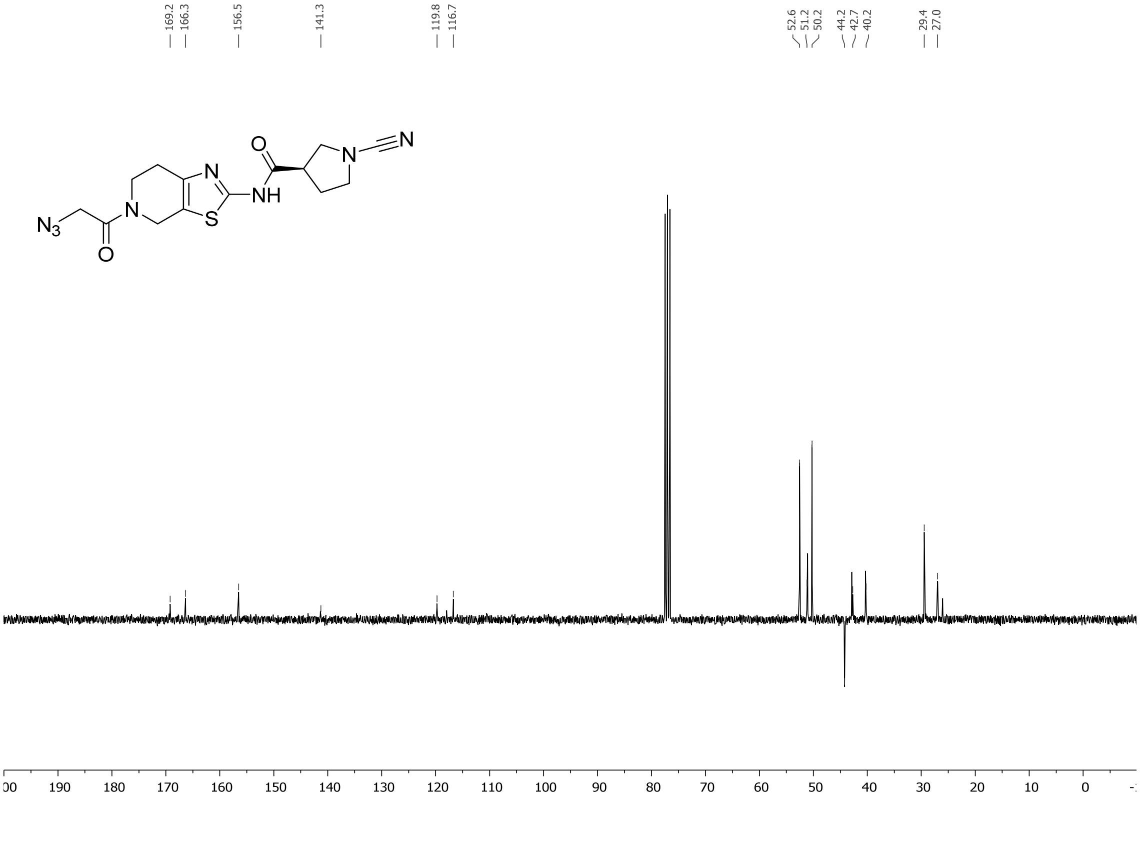

^{1}H-NMR and ^{13}C-NMR of compound JYQ-92 in DMSO.

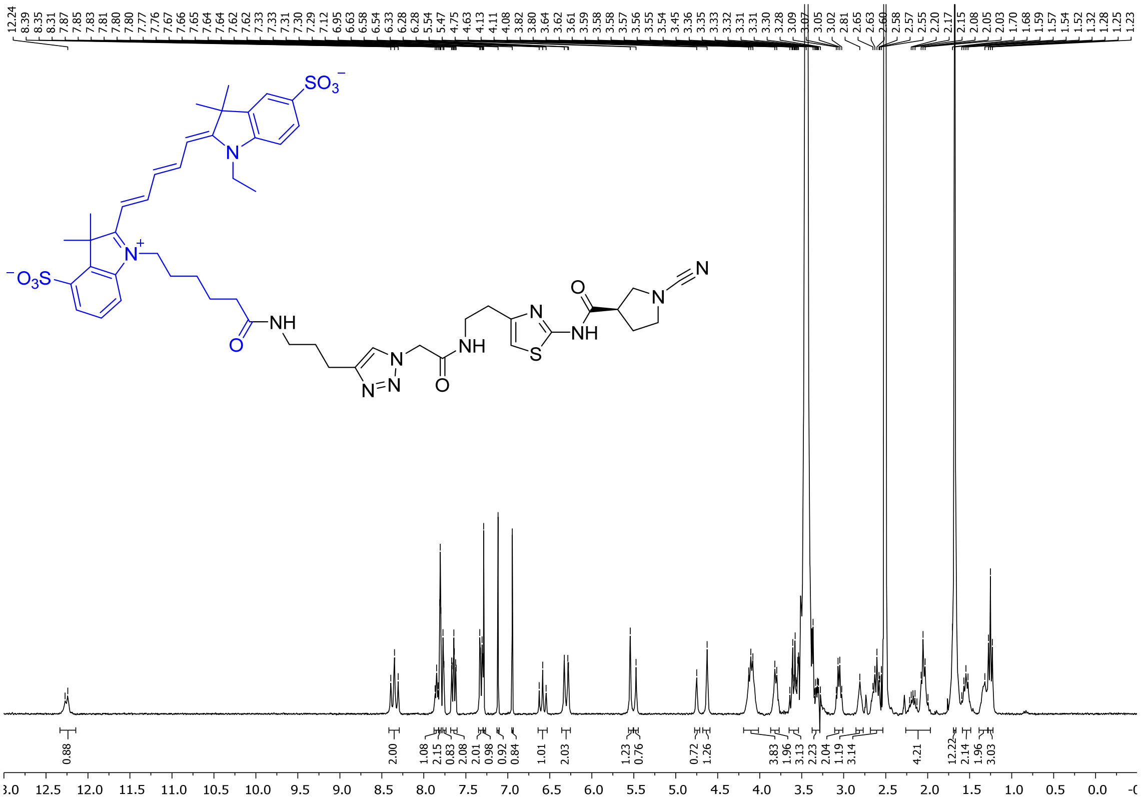

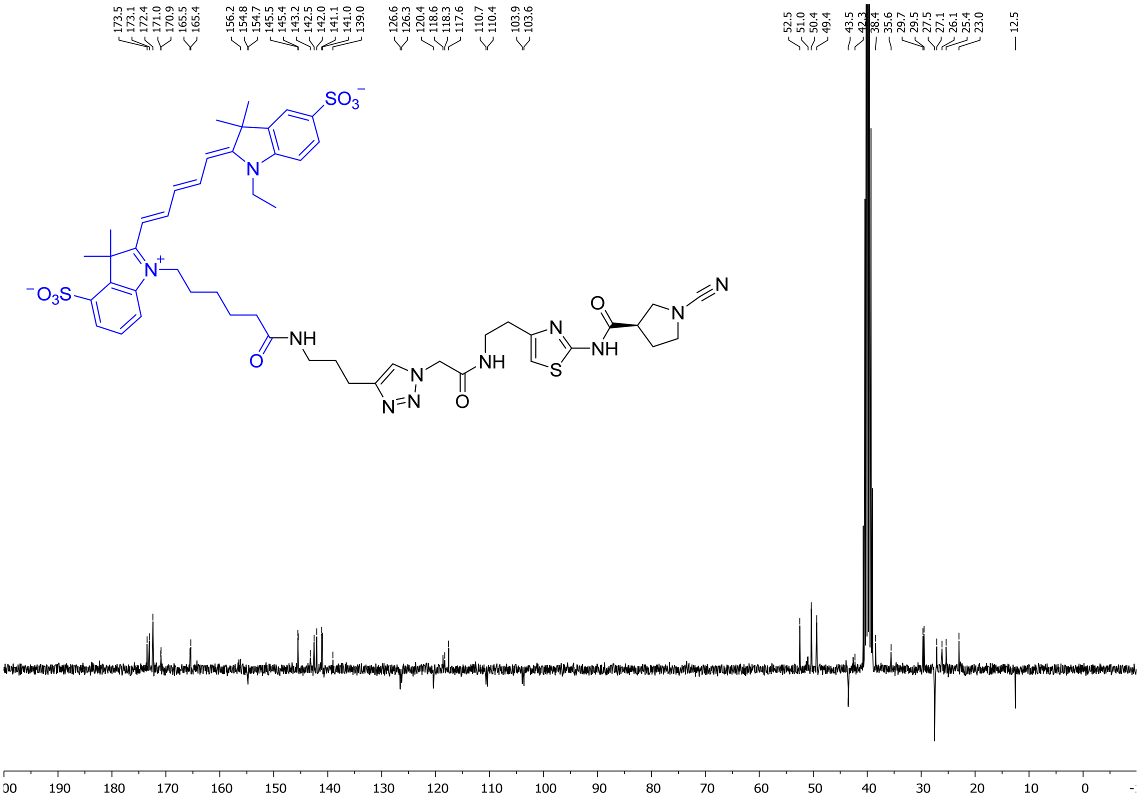

^{1}H-NMR and ^{13}C-NMR of compound JYQ-93 in DMSO.

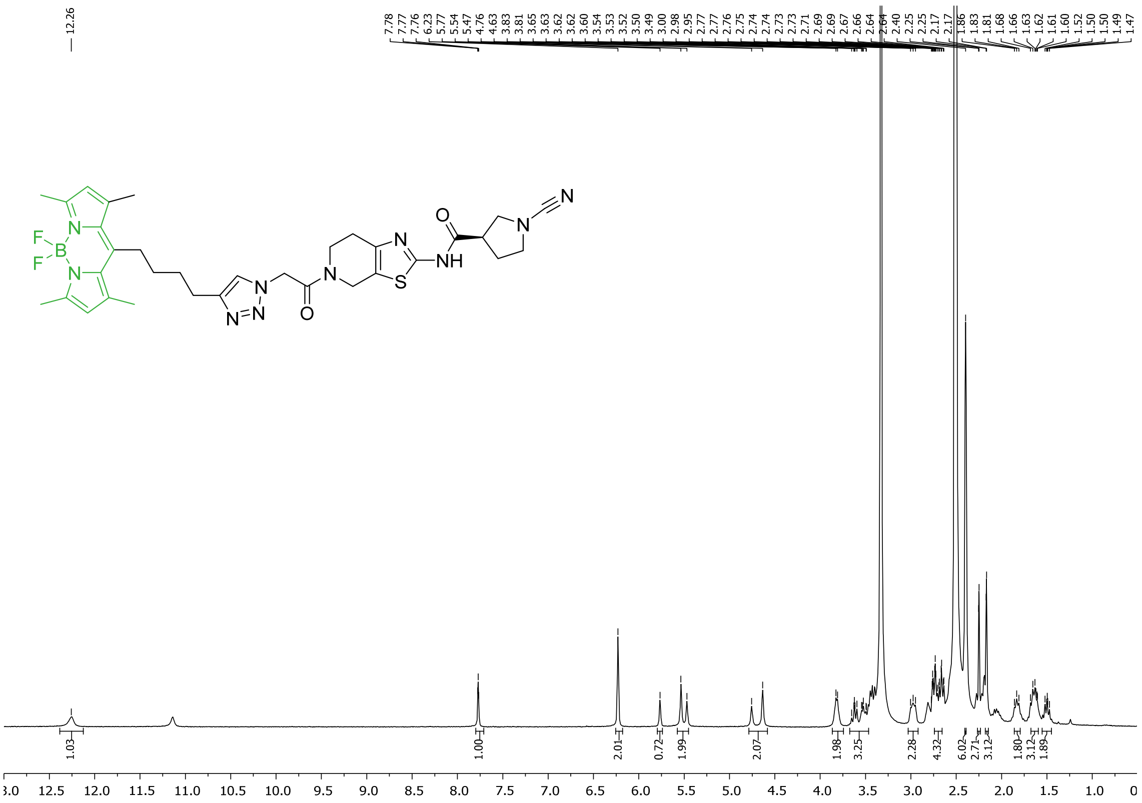

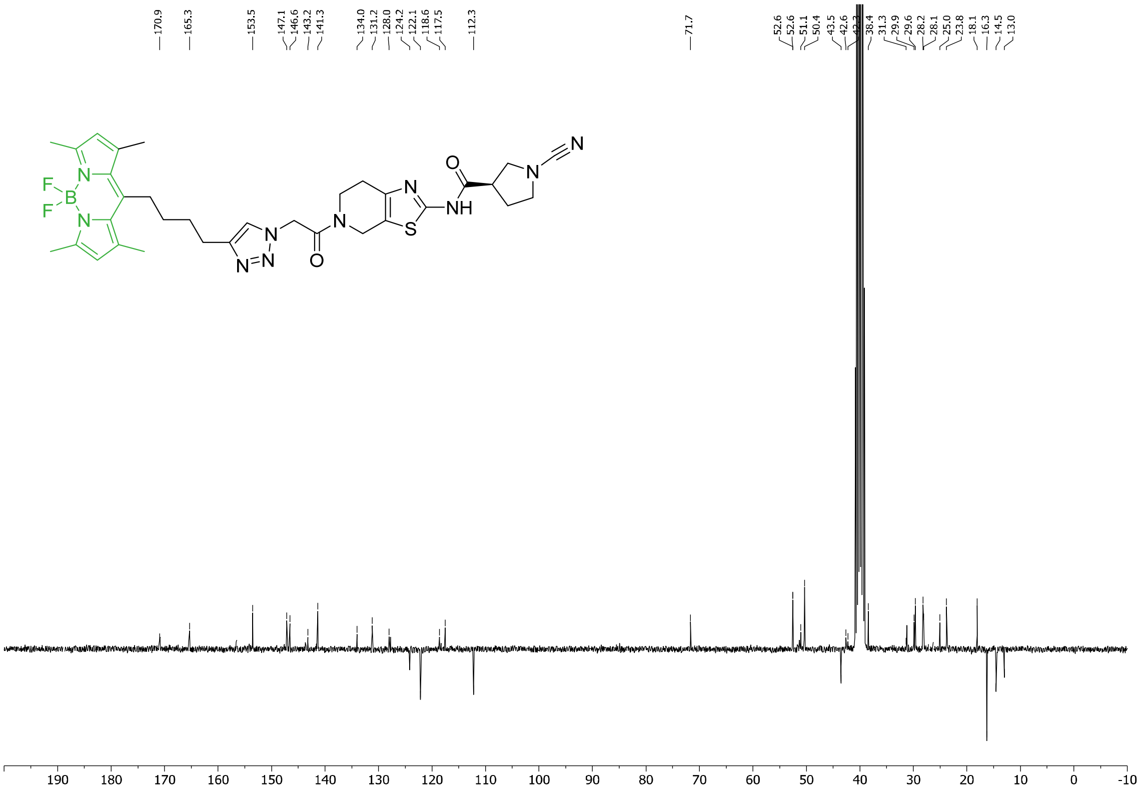

^{1}H-NMR of compound JYQ-107 in DMSO.

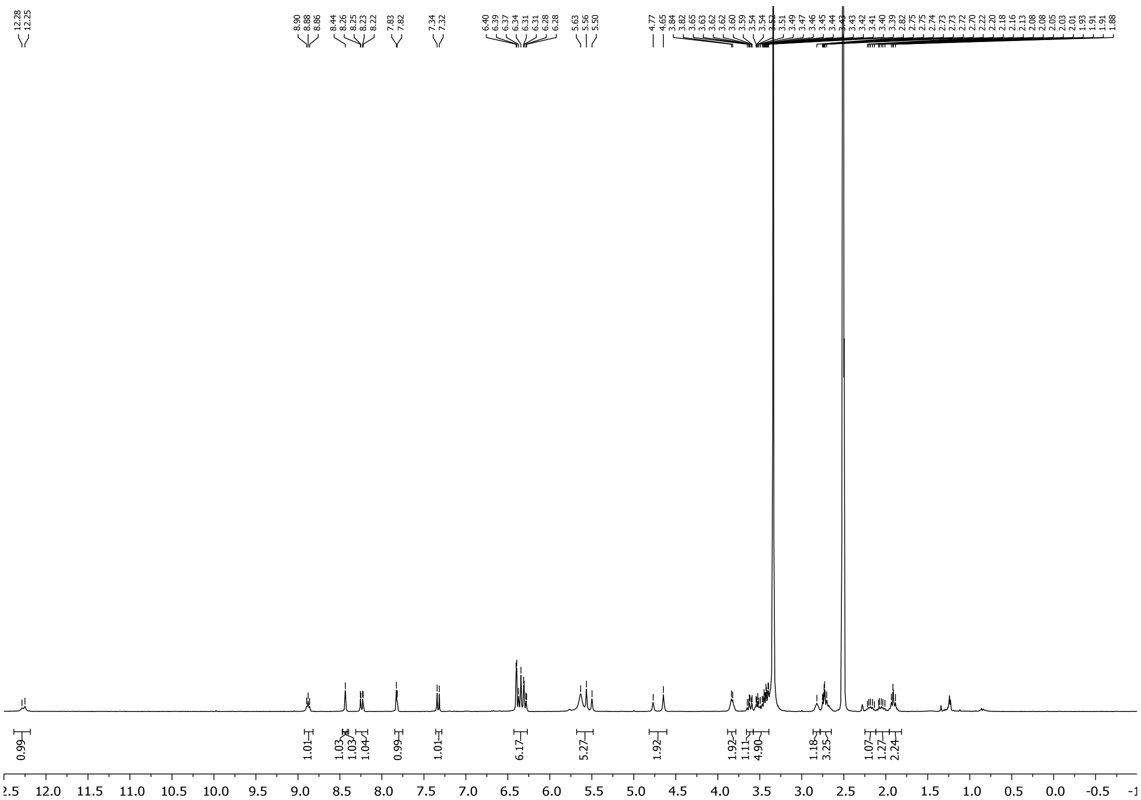

^{1}H-NMR and ^{13}C-NMR of compound STK793590 in DMSO.

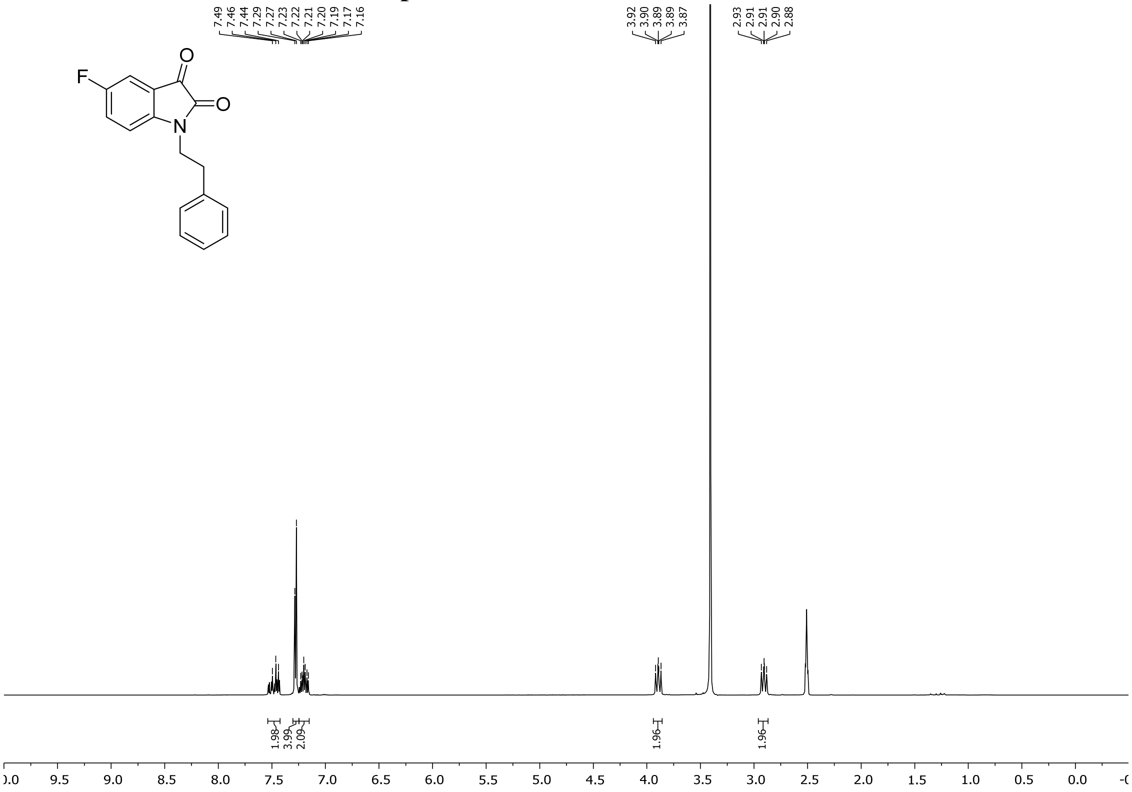

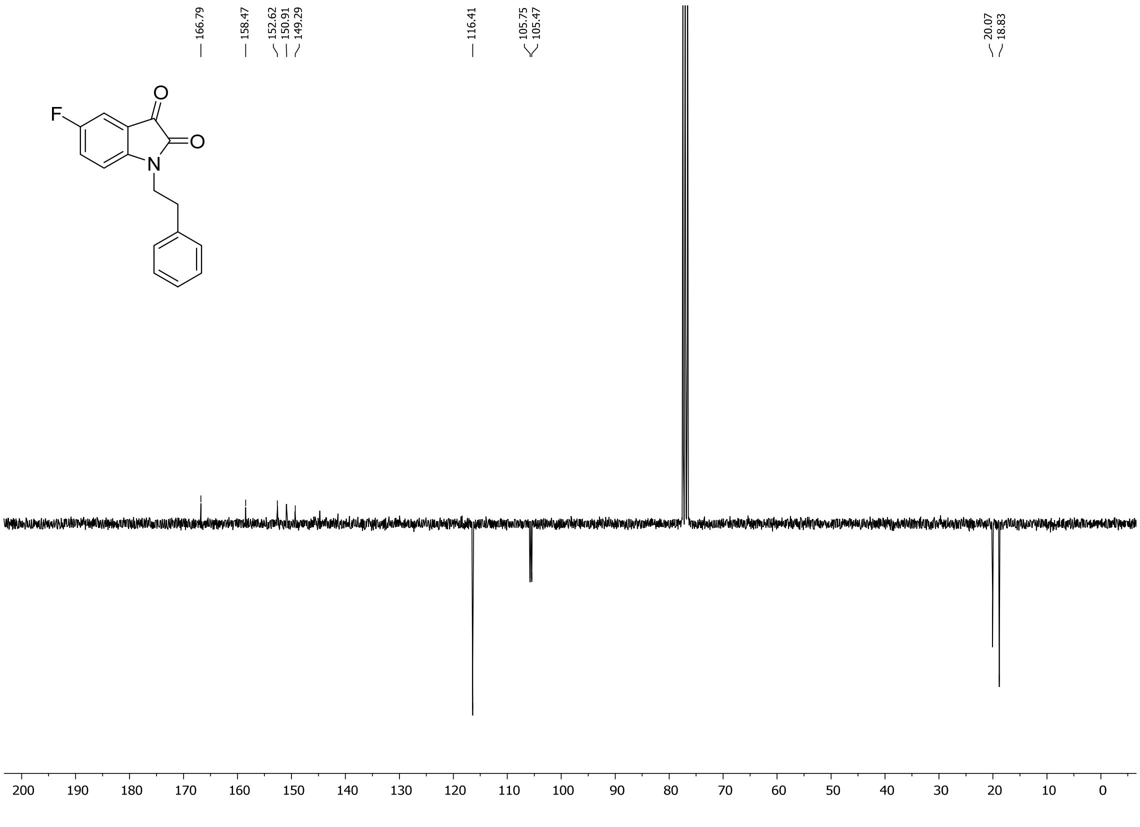

^{1}H-NMR and ^{13}C-NMR of compound DiFMuAc in DMSO.

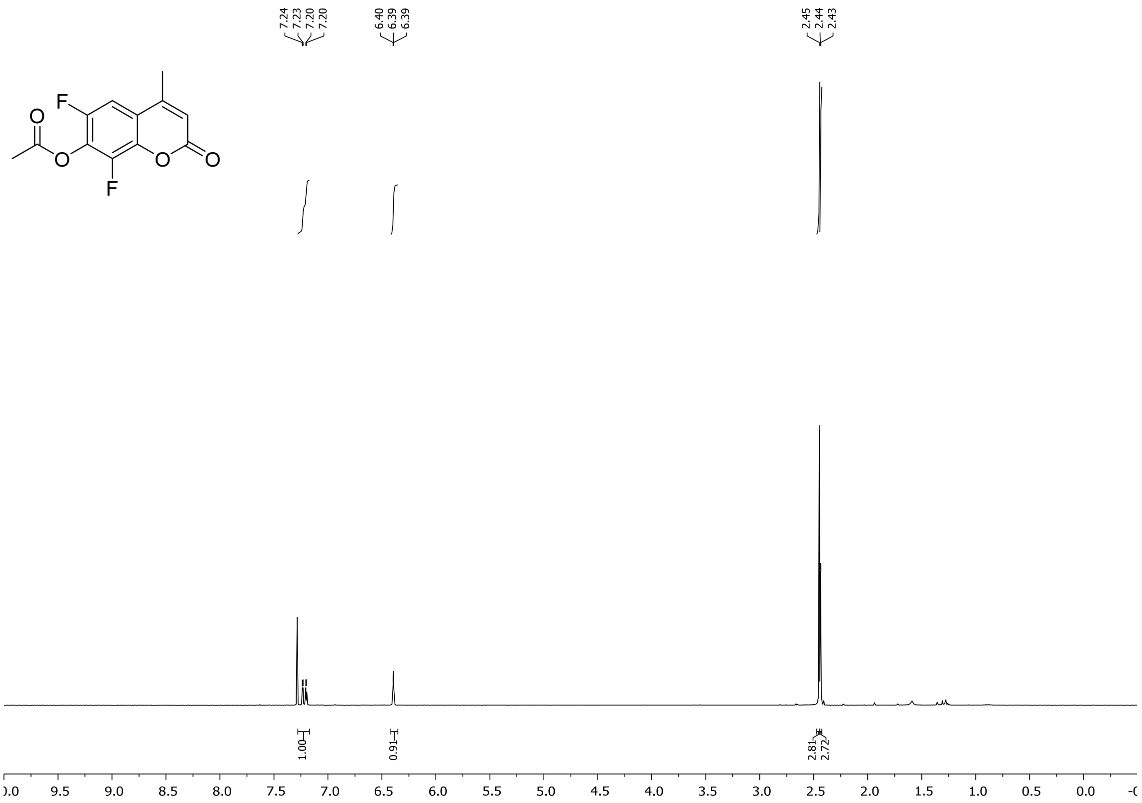

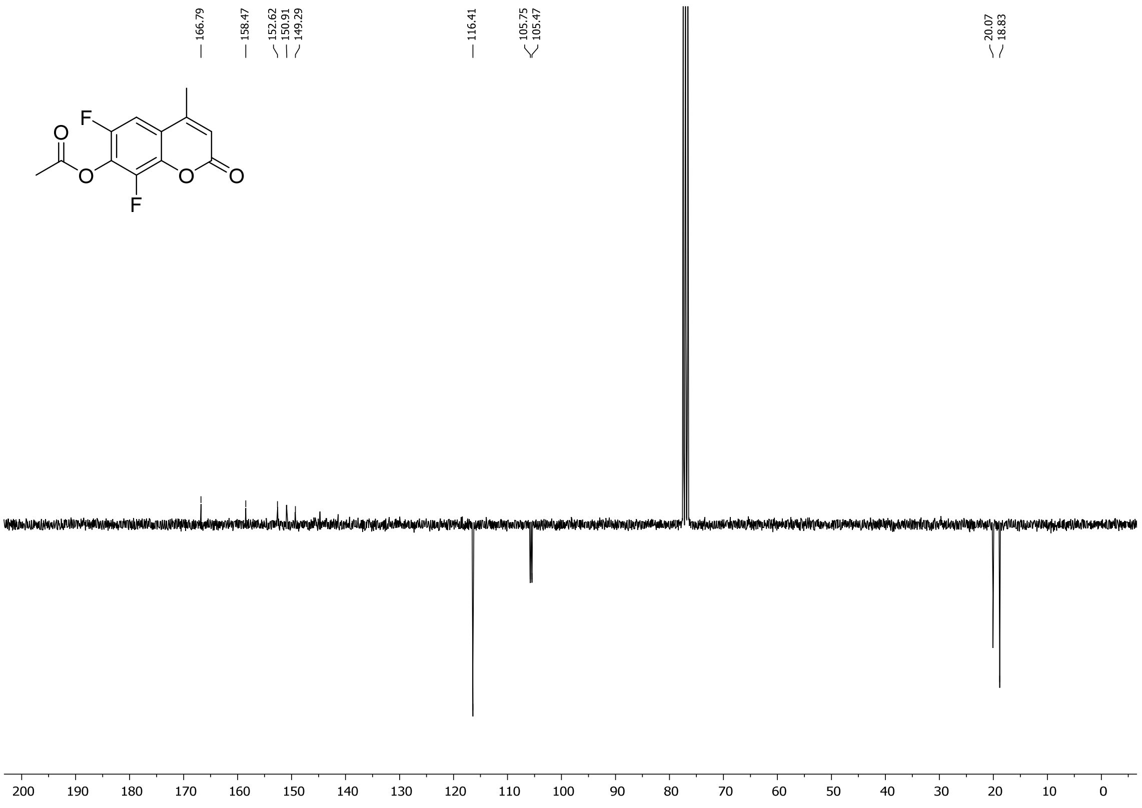

LC-MS of synthesized compounds

Compound JYQ-88: LC-MS

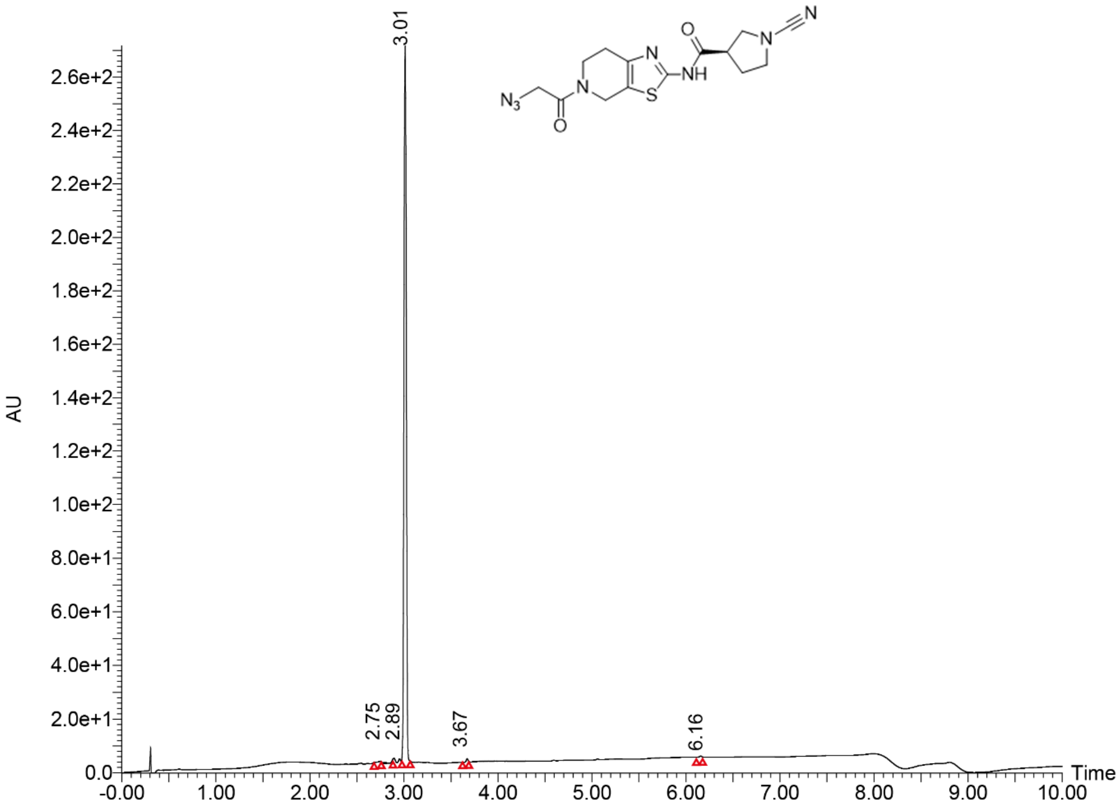

Peak	RT[min]	Height	Area	Area%
1	2.75	705504	50030.93	0.63
2	2.89	1641100	54265.17	0.68
3	3.01	266186032	7810046.00	98.25
4	3.67	11399782	24787.54	0.31
5	6.16	392171	9632.46	0.12

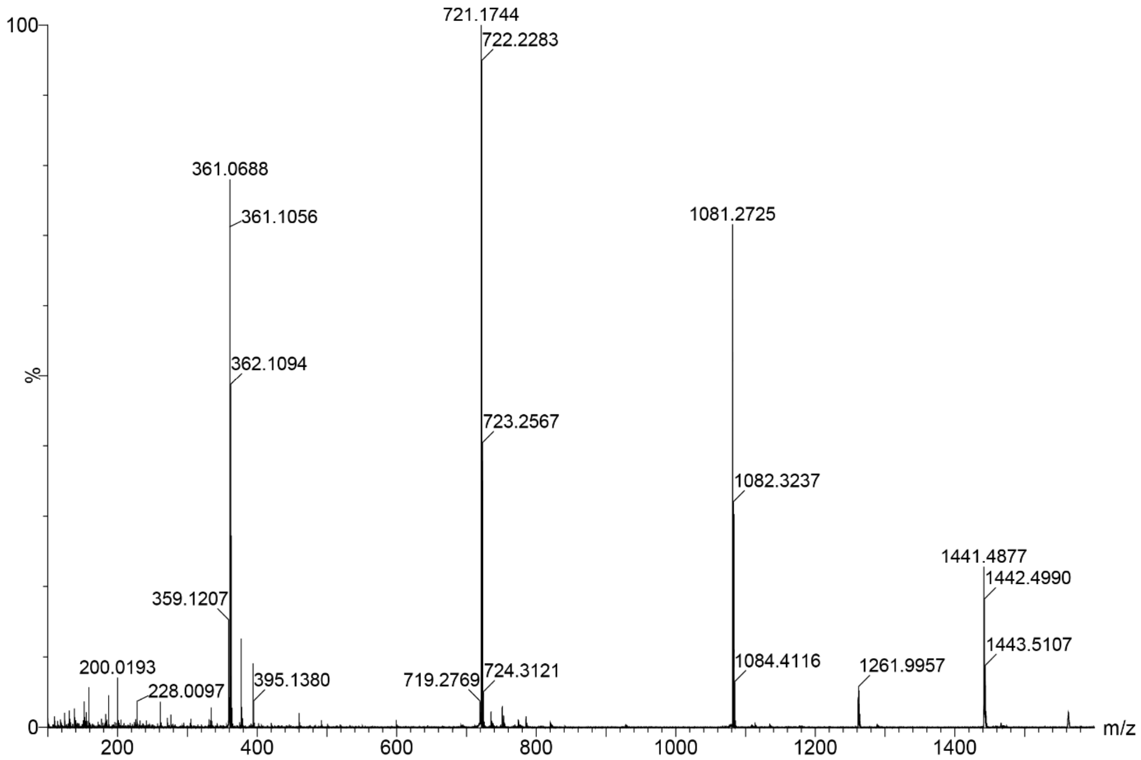

100
%
0
721.1744
722.2283
361.0688
361.1056
1081.2725
362.1094
723.2567
1082.3237
1441.4877
1442.4990
359.1207
1443.5107
200.0193
1084.4116
1261.9957
724.3121
719.2769
395.1380
228.0097
200
400
600
800
1000
1200
1400
m/z

Compound JYQ-92: LC-MS

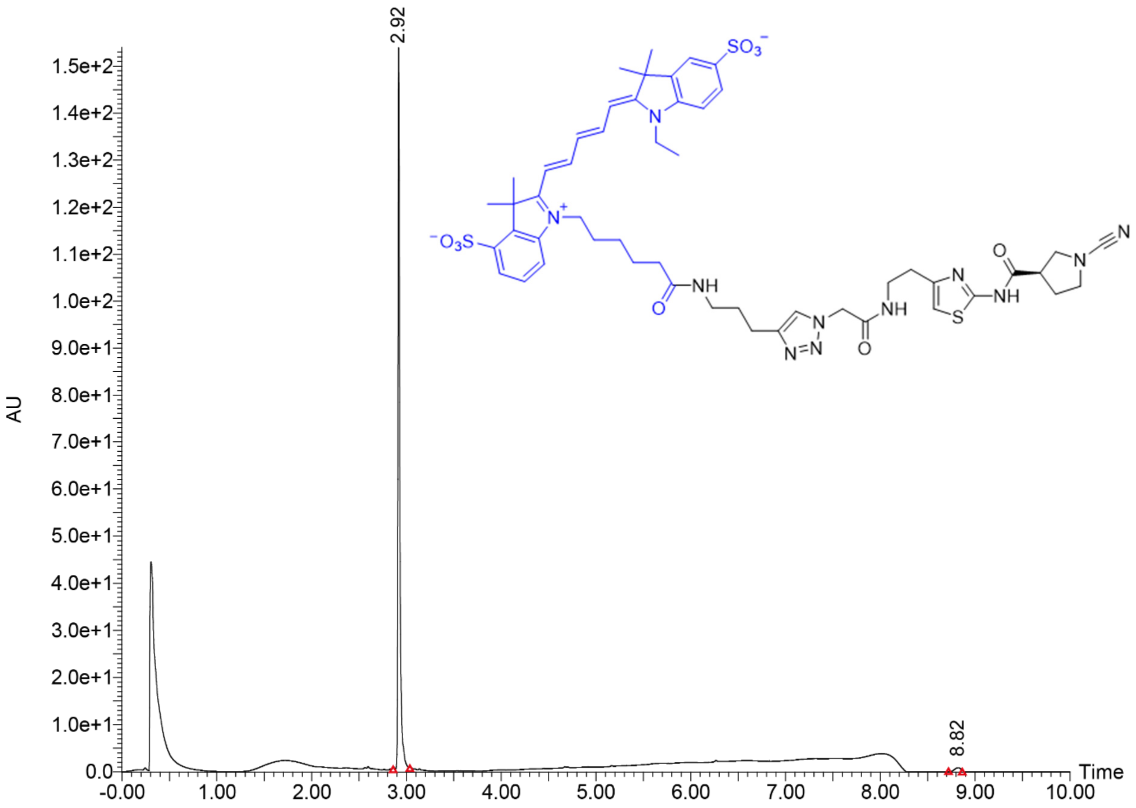

Peak	RT[min]	Height	Area	Area%
1	2.92	153148480	3473224.25	98.28
2	8.82	78243	60647.19	1.72

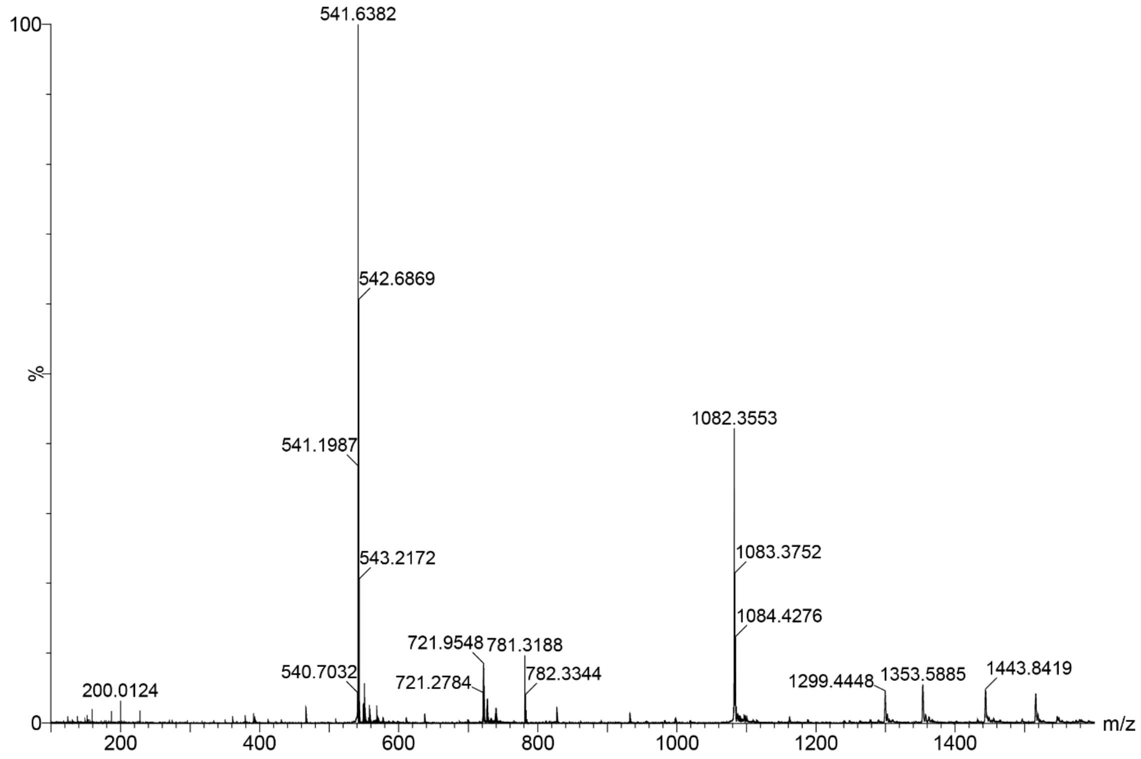

541.6382
542.6869
541.1987
1082.3553
543.2172
1083.3752
1084.4276
721.9548
781.3188
540.7032
782.3344
721.2784
1299.4448
1353.5885
1443.8419
200.0124
100
%
0
200
400
600
800
1000
1200
1400
m/z

Compound JYQ-93: LC-MS

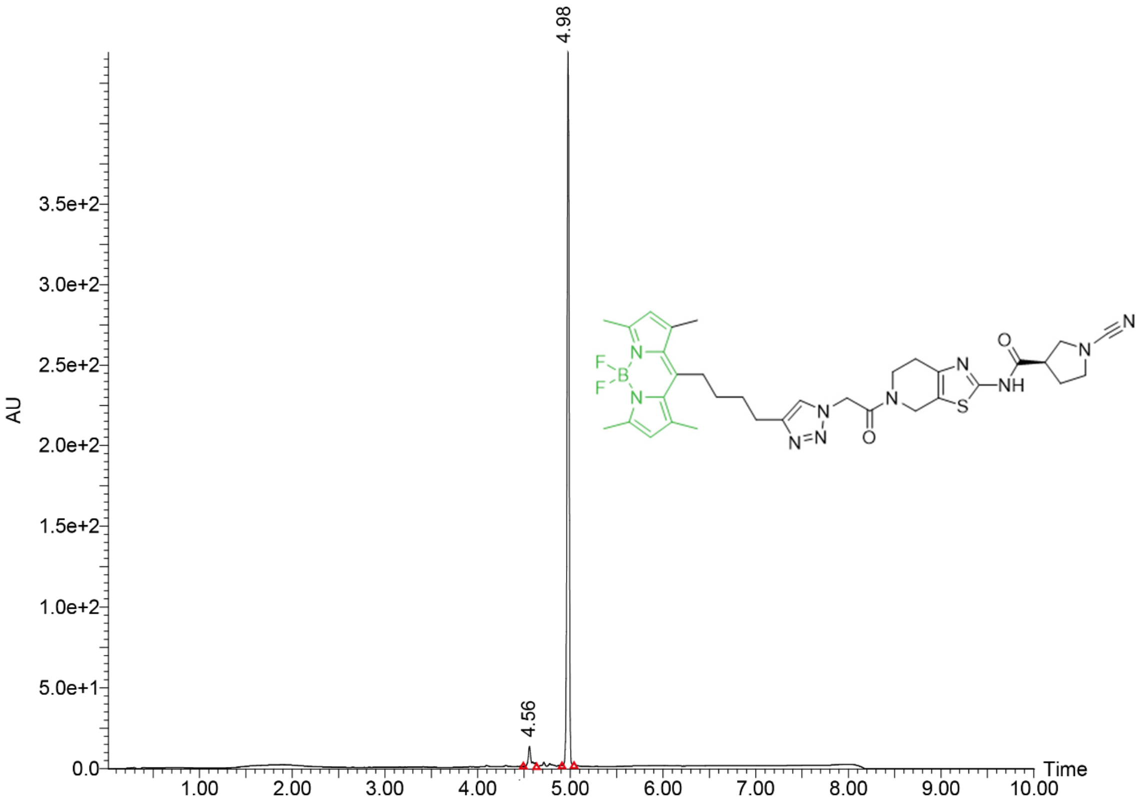

Peak	RT[min]	Height	Area	Area%
1	4.56	12353128	455077.31	3.72
2	4.71	2131677	44716.33	0.37
3	4.98	441885568	11721100.00	95.91

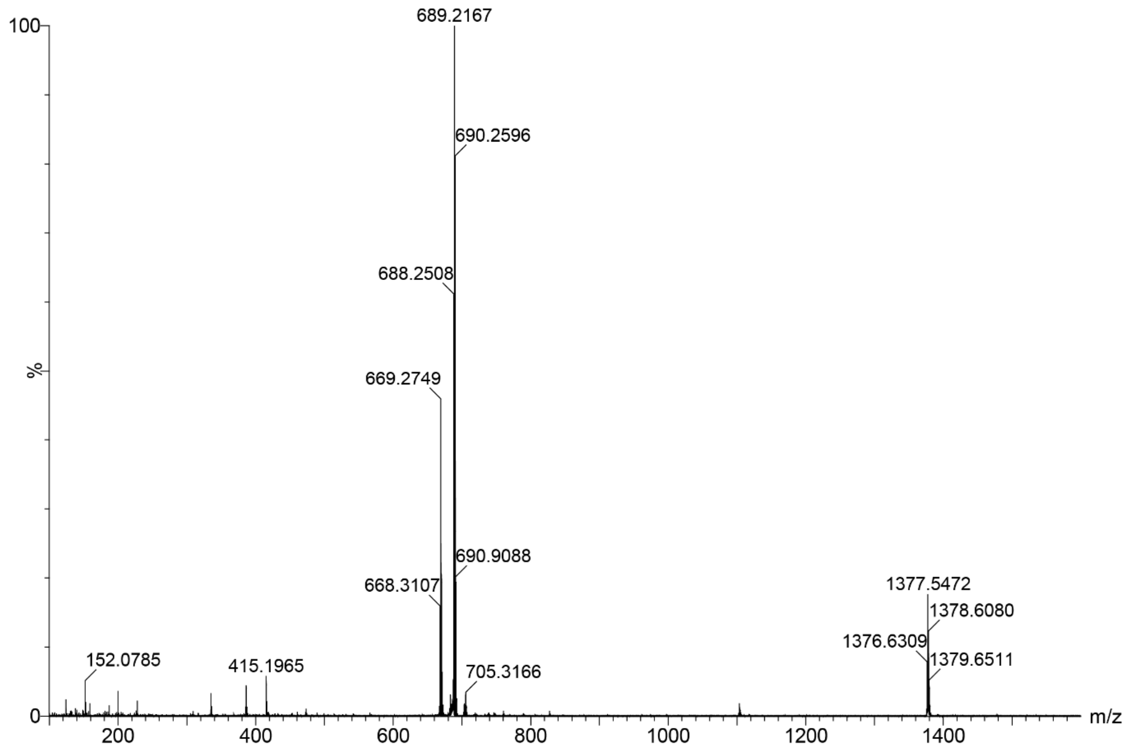
100
%
0
689.2167
690.2596
688.2508
669.2749
690.9088
668.3107
1377.5472
1378.6080
1376.6309
1379.6511
152.0785
415.1965
705.3166
200
400
600
800
1000
1200
1400
m/z

Compound JYQ-107: LC-MS

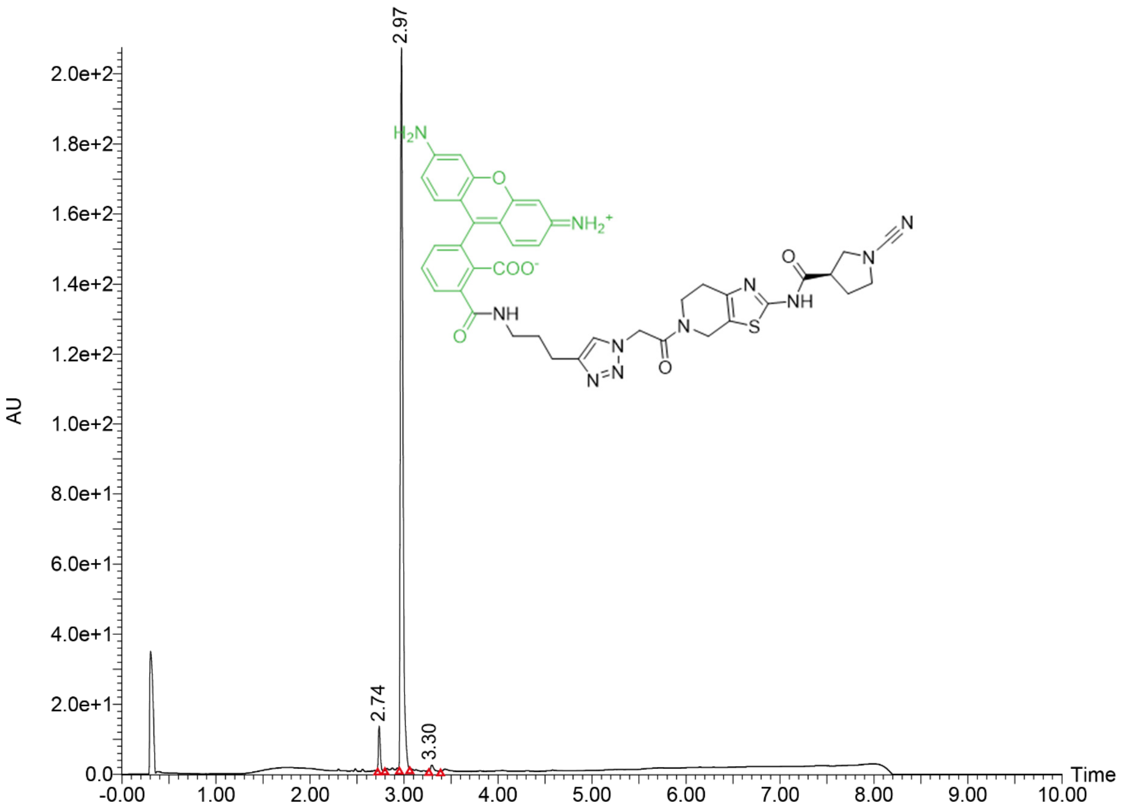

Peak	RT[min]	Height	Area	Area%
1	2.74	12701791	287764.28	4.15
2	2.97	205910896	6588283.00	95.06
3	3.30	1656870	54917.12	0.79

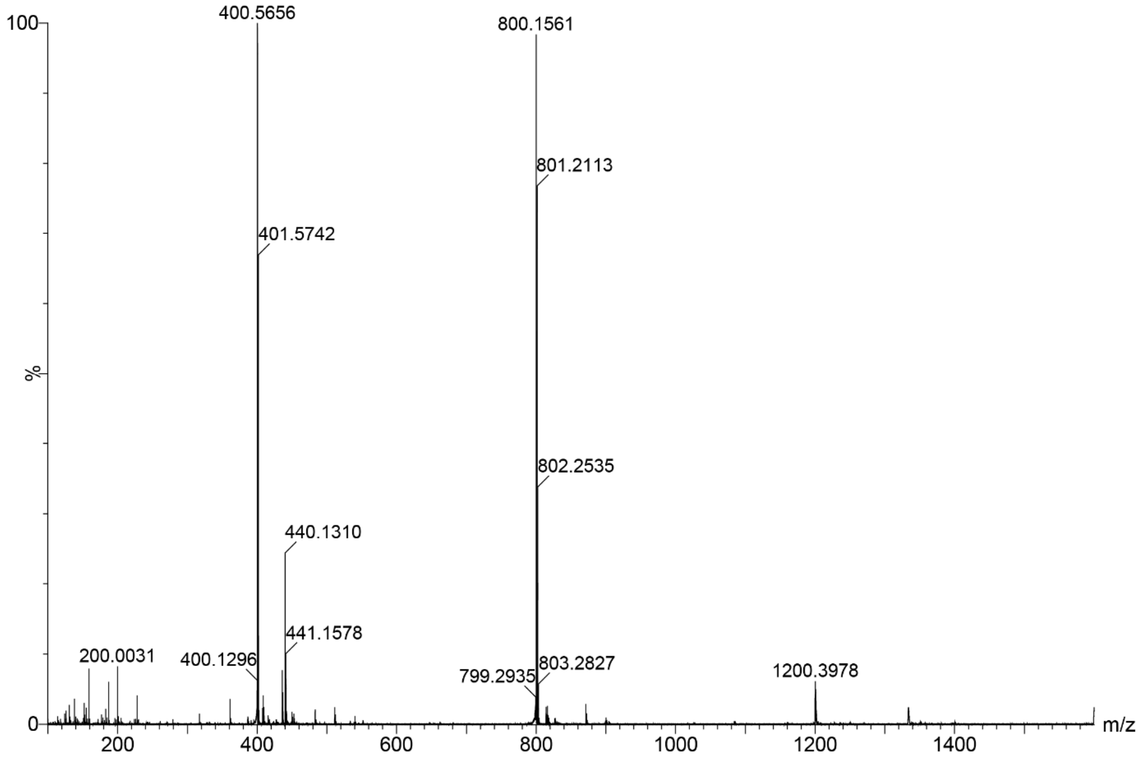
100
%
0
400.5656
800.1561
801.2113
401.5742
802.2535
440.1310
441.1578
200.0031
400.1296
803.2827
799.2935
1200.3978
200
400
600
800
1000
1200
1400
m/z
